# Research trends and geographical distribution of mammalian carnivores in Portugal (SW Europe)

**DOI:** 10.1371/journal.pone.0207866

**Published:** 2018-11-29

**Authors:** Joana Bencatel, Catarina C. Ferreira, A. Márcia Barbosa, Luís Miguel Rosalino, Francisco Álvares

**Affiliations:** 1 CIBIO/InBio, Centro de Investigação em Biodiversidade e Recursos Genéticos, Universidade do Porto, Vairão, Portugal; 2 UFZ—Helmholtz Centre for Environmental Research, Department of Conservation Biology, Leipzig, Germany; 3 Department of Biology, Trent University, Peterborough, ON, Canada; 4 CIBIO/InBIO, Universidade de Évora, Évora, Portugal; 5 Center for Environmental and Marine Studies–CESAM, Aveiro, Portugal; 6 Departamento de Biologia, Universidade de Aveiro, Aveiro, Portugal; Fred Hutchinson Cancer Research Center, UNITED STATES

## Abstract

Information regarding species’ status at a regional scale is instrumental for effective conservation planning. Some regions of southwestern Europe, such as Portugal, albeit included in the Mediterranean biodiversity hotspot, lack a detailed assessment of the distribution patterns of several taxonomic groups, such as carnivores. Moreover, information is scattered, often unreliable and biased towards some species or regions. This study aimed at reviewing the existing knowledge on mammalian terrestrial carnivores in Portugal, to analyse research trends, update the species checklist and assess their historical and current distribution patterns. We conducted a comprehensive review of 755 scientific studies to analyse several publication metrics and compiled 20,189 presence records of all mammalian terrestrial carnivores occurring in Portugal since historical times to evaluate their distribution patterns. Carnivore research in Portugal began in the 18th century, with a recent boost in the mid-1990s, and has been biased towards certain research topics and regionally threatened species. There are 15 extant species in Portugal, with nine occurring across the country, six showing a more limited range, as well as one additional species currently locally extinct (*Ursus arctos*). Over the last decades, the distribution ranges of seven species apparently remained stable, two expanded, two contracted, and three showed unclear trends. The presence of a new invasive carnivore, the raccoon (*Procyon lotor*), is also documented here. This study illustrates the relevance of a comprehensive analysis of non-systematic data to assess the historic and current status of mammalian terrestrial carnivores at a national level, and to identify knowledge gaps and research priorities.

## Introduction

Biodiversity is increasingly threatened by human-mediated habitat loss and climate change [1,2], raising concerns about the rate of decline of an escalating number of species [3]. Currently, at least a quarter of the world’s mammals are considered at risk of extinction [4]. The success of specific conservation actions implemented to revert population declines will rely heavily on the quantity and quality of data available on the species’ status and distribution [5]. These data are the baseline for a wide range of ecological, evolutionary and biogeographical studies [6,7,8,9], and hence updated information on species’ past and current distributions is paramount to prioritize conservation investment in a changing world [10].

Mammalian carnivores (Mammalia: Carnivora) are a charismatic group that illustrates well the relevance of collecting accurate species distribution data. They are important both at the species and the community level, having a strong effect on ecosystems both as functional regulators (e.g. seed dispersal, prey regulation; [11]) and providers of various ecosystem services (e.g. tourism attraction, intangible existence values; [12]). Furthermore, carnivores are often species of major concern, due to both conservation problems and the potential of conflict with humans [13,14]. They are thus the target of many management and/or conservation strategies, which need to be informed by parameters such as population trends and shifts in distribution ranges [15,16,17]. In this context, the combined use of historical and current distribution data provides an opportunity to investigate a wide array of ecological traits in carnivores, such as range expansions and/or contractions [18,19], timings and rates of spread of invasive species [20,21,22,23], environmental factors that shape past and contemporary presence [19,24,25], and how populations and communities respond to future environmental changes [26,27,28].

Efficient systematic monitoring schemes for carnivore species must encompass the integration of different, complementary methodologies, due to the elusiveness and ecological plasticity of this taxonomic group. However, in practice, implementing such schemes is challenging due to logistical and/or funding limitations [29,30,31,32]. Therefore, the use of non-systematic occurrence records scattered in the scientific literature or other available data sources can be useful to assess general distribution patterns. Yet, these data are often biased towards certain species, depending mostly on their biological traits, (e.g., body size, activity patterns, scent marking behaviour), as well as on conservation and management interests, research priorities and existing funding [33,34]. Moreover, when examining the information available for certain taxa, discrepancies emerge in relation to the reliability and spatio-temporal coverage of survey efforts [33,35]. These constraints highlight the importance of performing extensive reviews on carnivore research trends and geographical distributions based on available data, in order to identify knowledge gaps and define research priorities for each species [34].

The assessment of carnivore distribution patterns is particularly important in areas where human presence has a strong influence in the landscape, potentially shaping trends in species occurrence and promoting adaptation to disturbance [17,19,20,36]. This is the case of the Mediterranean region, a biodiversity hotspot [37] where anthropic disturbances have varied in frequency, scale and intensity over the past centuries [38]. Among the 38 carnivore species occurring in this region, two are endemic, almost a third is threatened, and at least two have become regionally extinct [39]. For successful conservation and management planning in this region, accurate and up-to-date species distribution data at a national level are essential [17,40,41].

Portugal, located at the southwestern tip of Europe, within the Mediterranean biodiversity hotspot, has a remarkable richness of mammalian carnivores considering its small size [15,42]. This is mostly due to its biogeographical and ecological features, as it is encompassed by both the Eurosiberian (northwest Portugal) and Mediterranean (central and southern Portugal) biogeographic regions [8]. Previous status assessments of mammalian carnivores in Portugal, dating from roughly 20 years ago [15,43], have considered 14 extant species, including two presumably introduced during historical times (the Egyptian mongoose, *Herpestes ichneumon*, and the common genet, *Genetta genetta;* [44]), and the more recently introduced American mink (*Neovison vison;* [21,22]). Detecting the occurrence of certain native species of mesocarnivores in Portugal, such as the pine marten (*Martes martes*) and the stoat (*Mustela erminea*), was only accomplished quite recently, with the first confirmed records dating from the late 20^th^ century [45,46]. The lack of systematic distribution data for these two species is the main reason why they are still classified as Data Deficient in the Portuguese Red Data Book [47], and this is a constraint that percolates across many taxa in this region. Indeed, data on the status and distribution of most Portuguese terrestrial mammals are outdated, scattered and scarce, and cartographic information detailing historical and recent distribution ranges is lacking for most species. This hampers the effective implementation of conservation and management strategies targeting mammalian carnivores in this region, which is mandatory due to the deep land use changes that have occurred in the country over the past decades [48]. Furthermore, the current lack of comprehensive distribution data for Portugal precludes complete and accurate mammal diversity assessments at wider scales, such as the Iberian Peninsula, Western Europe, or the Mediterranean biodiversity hotspot, as well as the national potential contribution to Biodiversity Observation Networks, like GEO BON (Group on Earth Observation Biodiversity Observation Network).

In this paper, we aim to review the existing body of knowledge on mammalian terrestrial carnivores in Portugal, in order to assess research trends, update the species checklist and investigate their distribution patterns. Specifically, we aimed to i) evaluate publication metrics related to carnivore research over time according to species, year of publication, publication type and research topic; ii) obtain an updated species checklist for the country, based on non-systematic data of extinct and extant native and non-native species; and iii) compile as many existing carnivore presence records as possible, considering their accuracy and extent, and produce distribution maps based on the current available knowledge. By using non-systematic data, this work provides the first attempt for a nation-wide assessment on carnivore distribution patterns in Portugal, at a relatively fine scale (10x10-km).

## Materials and methods

### Literature review and publication metrics

We conducted an extensive review of the scientific literature available on carnivores in Portugal. Relevant studies were identified using several search engines, including Google Scholar (http://scholar.google.com), ISI Web of Knowledge / Web of Science (WoS, www.wokinfo.com), Scientific Electronic Library Online (SciELO, www.scielo.org) and online archives of Portuguese universities (which can be accessed at http://www.uc.pt/fcdef/documentosbiblioteca/Bibliotecadigital/Repositorio). Grey literature consisted mainly of graduate and undergraduate theses that were obtained from all main Portuguese universities, such as the University of Lisbon, University of Porto, New University of Lisbon, University of Évora, University of Aveiro, University of Trás-os-Montes and Alto Douro, University of Minho and University of Algarve.

Several combinations of keywords were used to identify relevant publications for all carnivore species known to occur in Portugal: the scientific and common name (both in Portuguese and English), ‘carnivore’, ‘Portugal’ and ‘Iberian Peninsula’. Reference lists of publications were also used as bibliographic sources.

Publications were categorized by ‘Species’, ‘Publication Year’, ‘Type of publication’, and ‘Research Topic’. The ‘Type of publication’ category comprised: SCI articles (peer-reviewed), Non-SCI articles, Books (or book chapters), Conference Proceedings, Theses or dissertations (PhD and others) and Technical reports, following a rank in decreasing order of importance which was considered to remove duplicates, whenever the same study was published in different formats (e.g. SCI article and conference proceedings). ‘Research Topic’ refers to the publication’s main area of research and was defined as follows: ‘Conservation’ (studies related to human-wildlife conflicts; human perceptions and attitudes towards carnivores; illegal persecution; damages; habitat recovery; conservation action plans; impact of human activities), ‘General Ecology’ (trophic ecology; reproduction; habitat requirements and selection; home ranges; space use; activity; ecological modelling; scent-marking; behavioural responses; social ecology; abundance), ‘Genetics’ (phylogeography, population genetics, non-invasive genetics, hybridization, molecular markers), ‘Health Status’ (parasites, diseases, physiological parameters), ‘Population Status’ (past and present distribution patterns; population size; population trends and dynamics; population viability analysis; monitoring) and ‘Others’ (palaeontology; ethology; systematics; morphology; anatomy; methodological approaches; etc.). For simplicity, we specified that each publication could only fall within a single (main) Research Topic category (S1 Table).

The profile of research efforts on carnivores in Portugal was evaluated using three quantitative indicators: i) number of publications per year and per type of publication; ii) ratio between the number of publications focused on a single species vs. studies targeting multiple species; iii) number of publications per species and per research topic.

### Compilation of presence data

Presence data of all carnivore species were obtained from the aforementioned bibliographic references and complemented with records from other two main sources: i) online databases, such as the national Information System of Natural Patrimony (www.icnf.pt/portal/naturaclas/patrinatur/sipnat), the Global Biodiversity Information Facility (www.gbif.org) and Biodiversity4All (http://www.biodiversity4all.org); ii) unpublished data from individual expert researchers, universities, private companies and environmental associations in Portugal, through formal requests via e-mail. Records with errors or missing information related to species identification (e.g. details on identification criteria missing or inaccurate) and geographical location (e.g. imprecise locations or outliers from well-known ranges), were discarded. The majority of the reviewed literature and collected records came from sources and databases written in Portuguese and are therefore of limited accessibility to a non-Portuguese speaking audience.

All presence records were classified according to the date when the record was collected, as follows: ‘Historical Data’ included records prior to the year 2000; and ‘Current Data’ included records dated from 2000 to 2015. We considered the year 2000 as a threshold because data sharing dynamics underwent a huge development since that year (e.g. establishment of the Global Biodiversity Information Facility; [49]) and, as is demonstrated in our results, the amount of publications, particularly peer-reviewed ones, increased drastically from this year forward. For each record we also collected, whenever possible, the following information: geographical location (current data were georeferenced on UTM 10x10-km^2^ grid cells, while historical data were, mostly, only available at the Municipality level) and type of record (direct observation, photographic record, acoustic detection, questionnaire, scat, footprint). For current data, we additionally classified each record as ‘Confirmed’ or ‘Unconfirmed’ according to the level of accuracy and reliability of species identification assigned to the presence record. ‘Confirmed’ records included all unequivocal records, such as direct observation or capture of live animals, dead animals, specimen photos, and genetically identified samples. Records classified as ‘Unconfirmed’ involved reasonable uncertainty, such as presence signs without genetic confirmation, questionnaires, and records of unknown type. This allowed us to classify each cell in the UTM 10x10-km^2^ grid of Portugal (N = 1004) for current data into one of three categories: ‘No records’, ‘Unconfirmed’ or ‘Confirmed’ presence of each species. Distribution maps were built for each species containing both historical and current data, using the software QGIS 2.2.0 [50].

### Analysis of geographic range

For each species, we determined the Extent of Occurrence (EOO), for both the historical and current data, and the Area of Occupancy (AOO) based only on the current data, as described by Gaston [51] and following the guidelines for the IUCN Red List Categories and Criteria [52]. The EOO is described as the area contained within the shortest continuous imaginary boundary which can be drawn to encompass all the known, inferred or projected (e.g. from distribution models) sites of occurrence of a taxon. It corresponds here to the total area (within Portuguese administrative borders) measured using the Minimum Convex Polygon based on all geographical units with species presence, considering UTM 10x10-km^2^ cells for current records and Municipalities for historical records. The AOO, described as the area within the 'extent of occurrence' which is actually occupied by the taxon, corresponds to the sum of the area of UTM 10x10-km^2^ cells where the species has been recorded since 2000. To make these estimates comparable with other regions or countries, we converted them into percentages of the total area of mainland Portugal (88,742 km^2^). However, we stress that some care should be taken in interpreting the estimated AOO values, as species may not occupy all the areas included in each 10x10-km^2^, especially small size carnivores and habitat specialists (e.g. polecat, pine marten).

## Results and discussion

### Research trends and publication metrics on carnivores in Portugal

**Fig 1:**
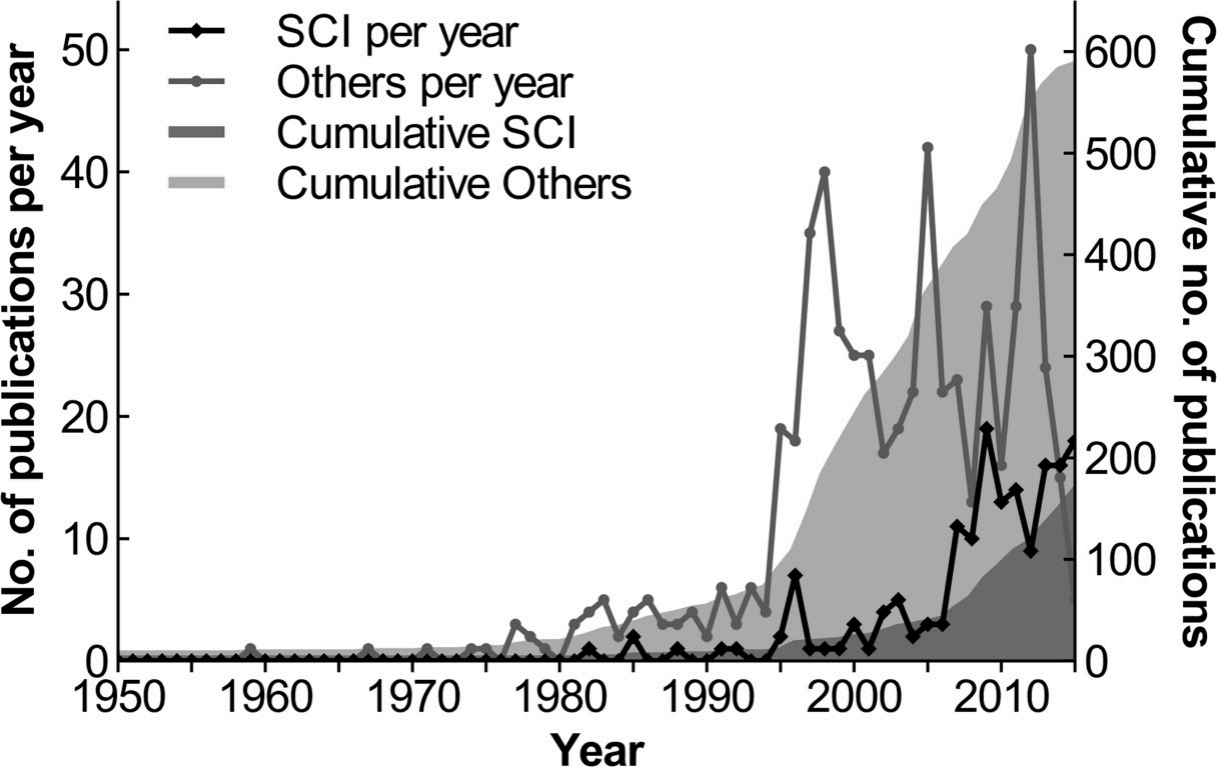
Annual total and cumulative number of publications (SCI and others) concerning mammalian carnivores in Portugal since the 1950s. Occasional publications dating from 1789, 1797, 1863, 1904 and 1910 were also found but are not included in the plot.

A total of 755 publications including data on carnivore populations from Portugal were used for the bibliometric analysis. The first scientific publications on Portuguese carnivores date as far back as 1789 and were concomitant with the foundation of the Academy of Sciences in Portugal [53,54]. The first SCI (peer-reviewed) article was published in 1982, but only in the mid-1990s did the number of SCI publications start to show a noticeable increase, which became more pronounced in the mid-2000s (Fig 1). This trend seems to be related to the creation, in the 1990s, of the first research groups within Portuguese universities that focused specifically on carnivore ecology (e.g. University of Lisbon), and is consistent with the worldwide overall pattern of increasing annual publication rate found for the order Carnivora [33].

**Fig 2:**
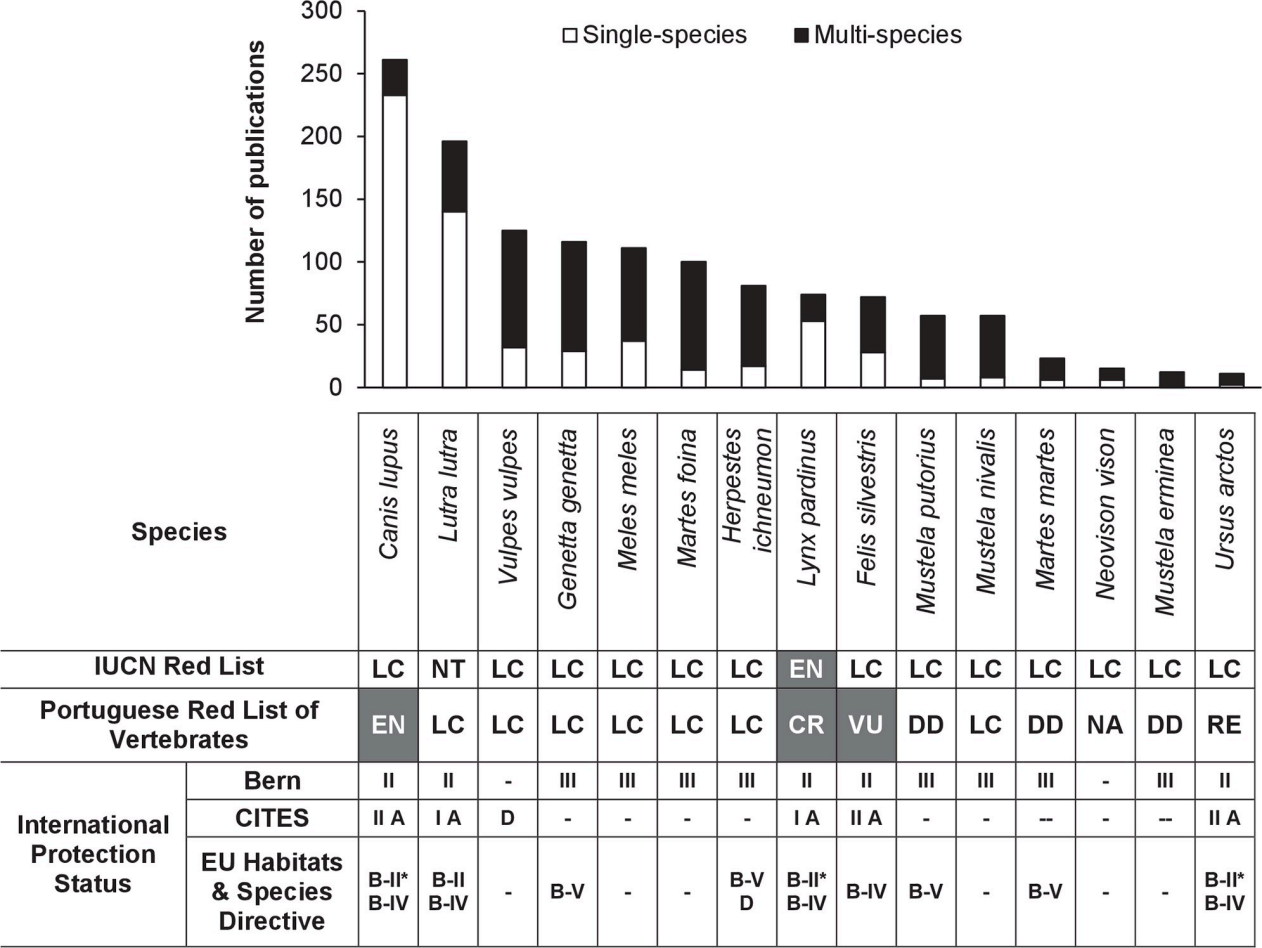
Number of carnivore publications in Portugal focusing on a single species vs on multiple species (N = 755), and the Red List Categories of each species according to the International Union for Conservation of Nature (IUCN) Red List of Threatened Species and the Portuguese Red List of Vertebrates. LC, Least Concern; DD, Data Deficient; NT, Near Threatened; VU, Vulnerable; EN, Endangered; CR, Critically Endangered; RE, Regionally Extinct; NA, Not Applicable (recently introduced). Species with threatened conservation status are marked in grey. The International legal status is represented by the inclusion in the Annexes of the Bern Convention (II, Appendix II; III, Appendix III), CITES (I A, Appendix I Annex A; II A, Appendix II Annex A; D, Annex D) and EU Habitats & Species Directives (B-II, Annex B-II; B-II*, Annex B-II*; B-IV, Annex B-IV; B-V, Annex B-V; D, Annex D).

Most publications (82%) were dedicated to a single species, and these were mainly focused on three carnivores [grey wolf *Canis lupus* (31%), Eurasian otter *Lutra lutra* (19%), and Iberian lynx *Lynx pardinus* (8%)], all of which are of conservation concern at a national and/or international level (Fig 2). This could be related to a general tendency for funding studies on iconic and/or threatened species, as suggested by previous reviews [33,34]. Also, these results are consistent with previous findings [35] that the carnivore species most studied in Europe during the last years are large in size, habitat generalists, or charismatic such as the grey wolf, the Eurasian otter and the European badger (*Meles meles*). In contrast, mesocarnivores that usually occur in sympatry and are surveyed with similar methodological approaches tend to be the target of multi-species studies focusing at the community level. The species with smallest numbers of publications included those currently extinct in Portugal (e.g. brown bear *Ursus arctos*) or with restricted distributions, low densities and/or elusive behaviour (e.g. pine marten, stoat, and American mink), which is also consistent with previous findings [33].

Regarding the type of publication, Conference Proceedings accounted for almost a third of the publications (32%), followed by SCI articles (22%), Theses (19%), Non-SCI articles (13%), Technical reports (9%) and Books or Book Chapters (6%). Compared to other regions where mammal publication metrics were investigated (e.g. [55]), Portugal has fewer scientifically peer-reviewed publications for carnivores. This might be due to the low stimulus given to researchers in Portugal to publish in SCI journals before 2000, probably associated with a disadvantage of non-native English speakers to publish their work in English-dominated journals [55]. However, the amount of Conference proceedings suggests that important efforts have been made to divulge in-house scientific work.

**Fig 3:**
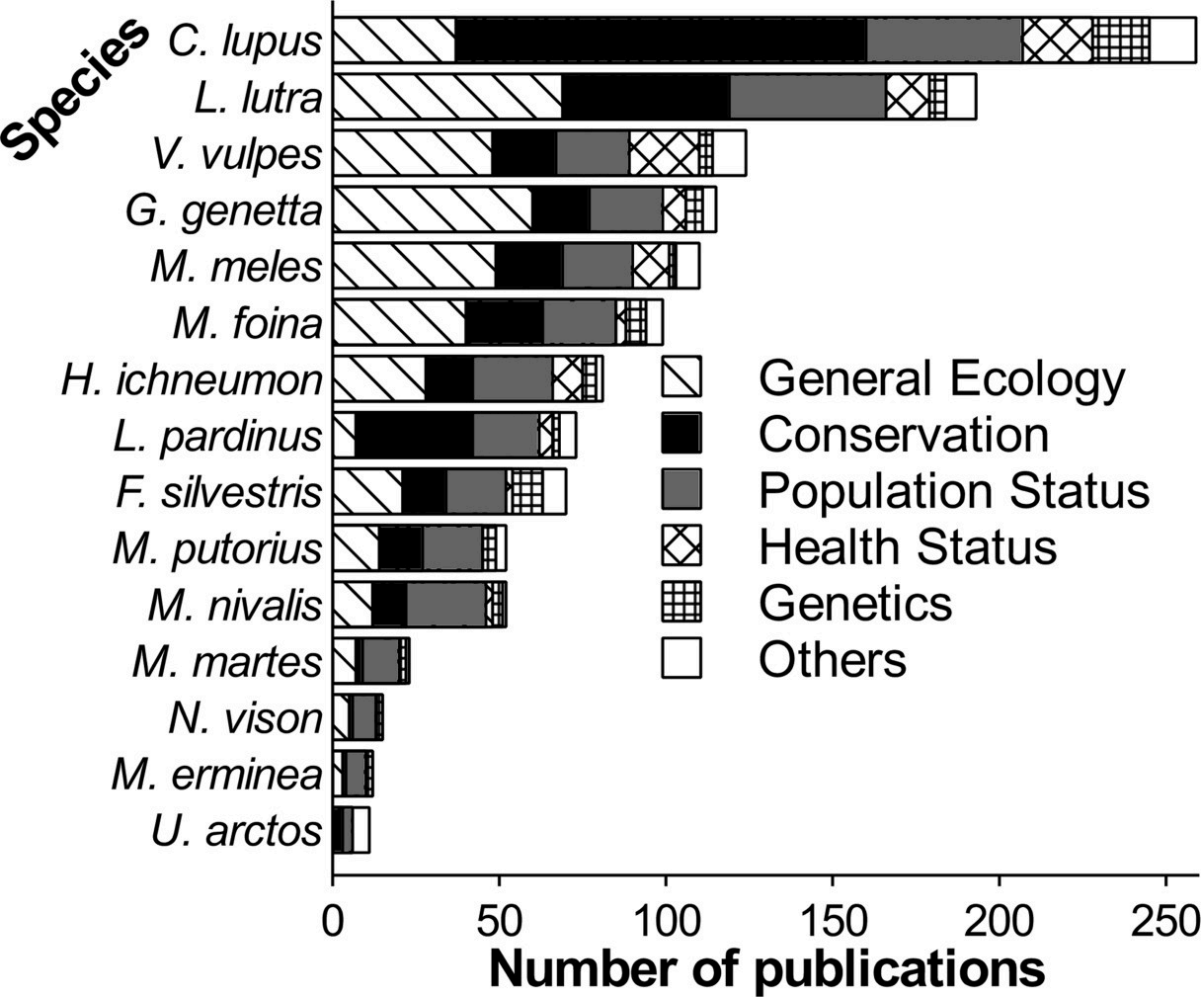
Number of publications per carnivore species in Portugal, grouped by research topic (N = 755).

The most common research topics investigated in carnivore studies were ‘General Ecology’ (32%), ‘Conservation’ (31%) and ‘Population status’ (18%), which is consistent with previous studies showing that at least one of these topics is among the top three research areas on other mammal species (Fig 3) [56,57,58]. In general, the patterns of research topics and carnivore species covered in publications from Portugal reflect the worldwide trends on carnivore research [35].

### Species checklist

In this study, we documented the presence in Portugal of 15 extant species of carnivores and one more that occurred in historical times, placing it among the European countries with highest carnivore species richness, especially considering its small size and marginal position in a pan-European scale [15,42]. According to the Portuguese Red Book of Vertebrates [47], one species is classified as Regionally Extinct, three are included in categories of threat (CR, EN and VU), three are classified as Data Deficient (DD), seven are classified as Least Concern (LC), and one recent invader (American mink) has the status of Not Applicable (NA; Fig 2). An additional exotic species, the raccoon (*Procyon lotor*), was now recorded for the first time in Portugal based on unpublished recent sightings of single individuals in four distinct locations and years (more details below) suggesting that it might be related to different introduction events and apparently with no current breeding populations.

The mammalian terrestrial carnivore community in Portugal is composed of species with different biogeographic origins: most species (N = 10) are native to Portugal and widespread in the Palearctic (e.g. grey wolf, red fox *Vulpes vulpes*, wildcat *Felis silvestris*) or Eurasia (e.g. Eurasian otter, European badger, stone marten *Martes foina*, pine marten, stoat, least weasel *Mustela nivalis;* western polecat *Mustela putorius*; [15]). All these species are or were widespread across mainland Portugal, except for the stoat and pine marten, which always occurred only marginally in northern Portugal due to their affinity to the Eurosiberian biogeographic region [15]. One additional species, the Iberian Lynx, is endemic and restricted to the Iberian Peninsula, i.e., mainland Portugal and Spain [59]. Two species with origin and core distributions in Africa, the common genet and the Egyptian mongoose, seem to have distinct biogeographical histories. While the genet was historically introduced in Europe, and naturalised populations now occur in the Iberian Peninsula [44], the mongoose, which until recently was also considered historically introduced, should be now classified as native due to recent genetic evidences suggesting its occurrence in Iberia during the late Pleistocene [44,60].

The invading American mink and raccoon, both native to North America, are currently spreading throughout the Iberian Peninsula [22,23]. For Portugal, the occurrence of the American mink was first documented in the late 1980s [21], while the raccoon is documented for the first time in this study. In fact, we gathered two confirmed observations of racoons in different sites from North of Portugal (a photographed live specimen in 2008 near Vila Nova de Famalicão and a captured live specimen in 2014 near Esposende), as well as two additional unconfirmed records in Central Portugal (reported sightings attributed to this species in 2012 near Santarém and in 2013 near Cascais). Apart from these two recent invaders, this study confirmed that all remaining species have an established occurrence in Portugal since historical times. This includes the pine marten and stoat, although their occurrence in Portugal was only scientifically recognized in the 1990s [43]. As for the brown bear, previous assessments consider the time of extinction in Portugal to be the mid-17^th^ century [43,47,61]. However, the present study compiled evidence of brown bear occurrence in Portugal until the late 19^th^ century, most probably as a result of occasional incursions of dispersing individuals from relict breeding populations in NW Spain, which at the time occurred near the Portuguese border [62].

The Iberian lynx, despite having a wide historical range at a national level, was considered on the verge of extinction at the onset of the 21^st^ century as its presence was not confirmed in Portugal during a national survey in 2002–2003 [63]. However, this species has recently recovered in Portugal as a result of natural dispersal movements from captive-bred individuals released under reintroduction programmes, both in Spain and Portugal [64,65,66]. The present study has also compiled several historical records of lynx in NW Portugal up to the 18^th^ century. However, these records are within the Eurosiberian biogeographic region and may thus suggest the former presence of another species in Portugal: the Eurasian lynx (*Lynx lynx*), which would be currently extinct. Indeed, recent genetic and historical evidence suggests that the Eurasian lynx occurred in the Atlantic-Alpine climate area of the northern Iberian Peninsula (including NW Portugal) from the Pleistocene until the early-19^th^ century [20,67], as opposed to the Iberian Lynx, which occurred further south, within the Mediterranean biogeographic region.

### Distribution patterns of Portuguese carnivores

We compiled a total of 20,189 presence records, 5,217 of which were historical records (since the 12^th^ century up to the 20^th^ century) and 14,972 were current records (from 2000 to 2015). Among current records, almost 75% came from previously unpublished data, 11% were obtained from online databases (not all of which are publicly available), and 14% came from the published literature.

The red fox and Eurasian otter were the species with the highest number of current presence records (Table 1), likely due to the conspicuousness of their presence signs, their widespread distributions [59] and relatively high abundance [68,69]. There was a high number of historical records for the Eurasian otter and the grey wolf, due to the existence of previous systematic and detailed assessments of their past distributions in Portugal [70,71].

**Table 1:** Common and scientific names of the 16 carnivore species recorded in mainland Portugal since historical times, total number of historical (pre-1999) and current (post-2000) records compiled for each species, and their current Area of Occupancy (AOO) and historical and current Extent of Occurrence (EOO).

		Historical Data			Current Data						
					Number of records	AOO				EOO	
English Common Name	Scientific Name	Number of records	All Records (km2)	EOO/Total country (%)		All Records (km2)	AOO/Total country (%)	Confirmed Records (km2)	Confirmed AOO/ Total AOO (%)	All Records (km2)	EOO/Total country (%)
**Grey wolf**	***Canis lupus***	1687	88527	100	567	18327	20,7	7973	44	32000	36,1
**Red fox**	***Vulpes vulpes***	89	87212	98	3377	32886	37,1	21471	65	87976	99,2
**Stoat**	***Mustela erminea***	46	11498	13	16	1122	1,3	596	53	10260	11,6
**Least weasel**	***Mustela nivalis***	56	61541	69	281	6210	7,0	2789	45	84097	94,8
**Western polecat**	***Mustela putorius***	81	68960	78	177	5848	6,6	2493	43	78247	88,2
**American mink**	***Neovison vison***	49	13508	15	84	3355	3,8	1655	49	13808	15,6
**Stone marten**	***Martes foina***	44	52419	59	1515	14127	15,9	8764	62	84172	94,9
**Pine marten**	***Martes martes***	71	27339	31	105	9215	10,4	404	4	34870	39,3
**European badger**	***Meles meles***	96	63640	72	1992	15035	17,0	7336	49	86465	97,5
**Eurasian otter**	***Lutra lutra***	1400	88727	100	3061	82231	92,7	5070	6	88677	100
**Common genet**	***Genetta genetta***	58	78335	88	1330	15343	17,3	10144	66	86696	97,8
**Egyptian mongoose**	***Herpestes ichneumon***	675	66118	75	2340	76800	86,6	51680	67	83470	94,1
**Wildcat**	***Felis silvestris***	490	86963	98	113	5586	6,3	3389	61	78461	88,5
**Iberian lynx**	***Lynx pardinus***	326	82030	92	5	471	0,5	471	100	9868	11,1
**Raccoon**	***Procyon lotor***	-	-	-	4	288	0,3	122	42	9116	10,3
**Brown bear**	***Ursus arctos***	49	64382	73	0	-	-	-	-	-	-

Current AOO and EOO were based on the total number of UTM 10x10-km^2^ cells where each species was recorded. Historical EOO was based on the total number of municipalities where each species was recorded. All measures are presented as percentage of the total area of mainland Portugal. All Records pertains to the total area covered by both confirmed and unconfirmed records in each time period for each species. Species are listed by phylogenetic order.

**Fig 4:**
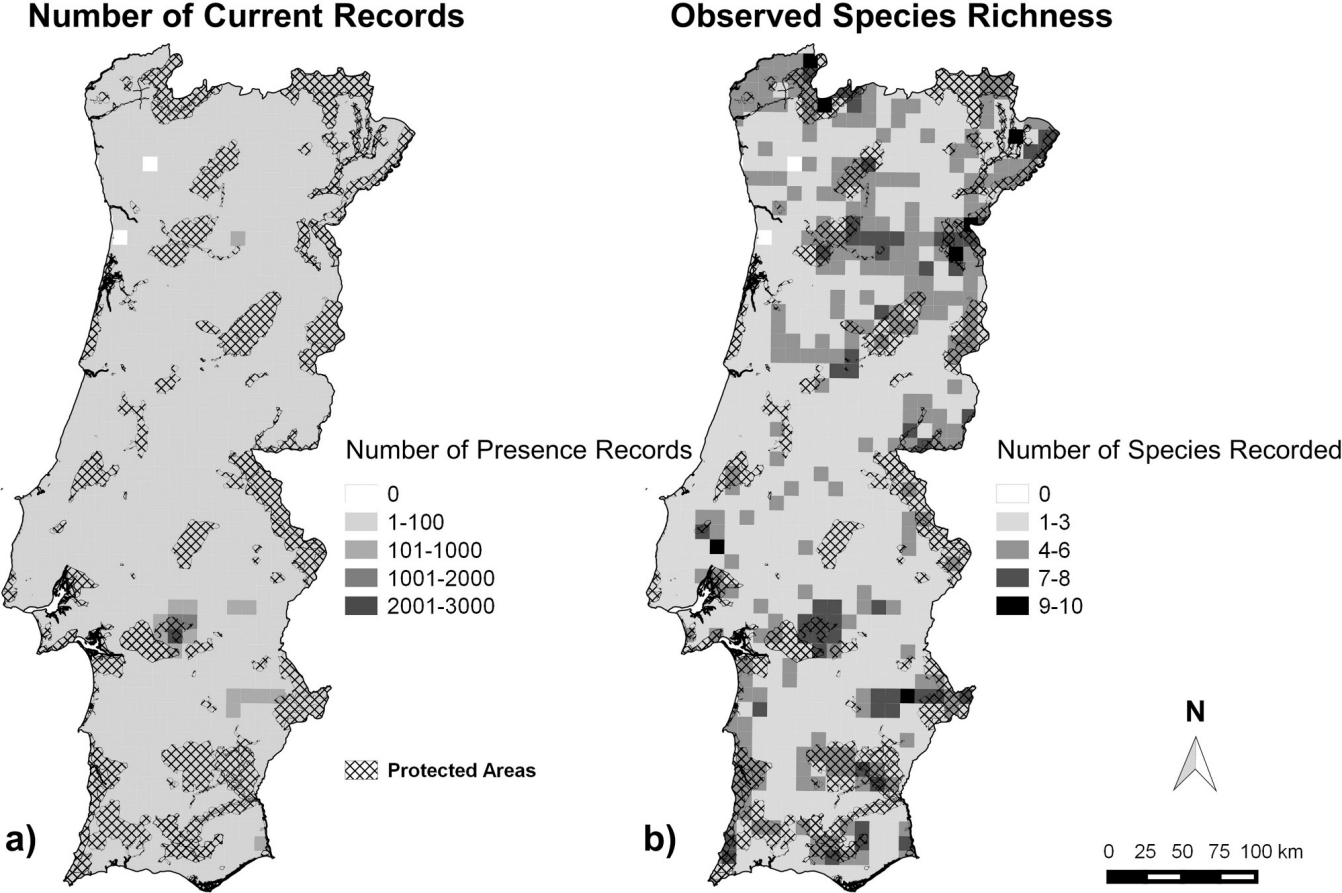
**Number of presence records (a) and Observed Species Richness (b) since 2000 in UTM 10x10-km^2^ cells in mainland Portugal, and location of the Protected Areas for reference**.

Sixty-eight percent of the historical records were from the 1980s and the 1990s. Only three species had historical data covering a much wider temporal period: the grey wolf, with records evenly distributed since the beginning of the 20^th^ century; the Iberian Lynx, with records available since the 18^th^ century, although mostly from the 1980s and 1990s; and the brown bear, with presence records from the 12^th^ to the 19^th^ century. For current records, only 18% (N = 2,707) were classified as ‘Confirmed’. The geographic distribution of the current data (Fig 4A) is fairly homogenous across mainland Portugal, except for an area in central-south Portugal, where a disproportionally large amount of data is available due to a particularly important mammal sampling effort made over several years (Monfurado Natura 2000 site).

**Fig 5:**
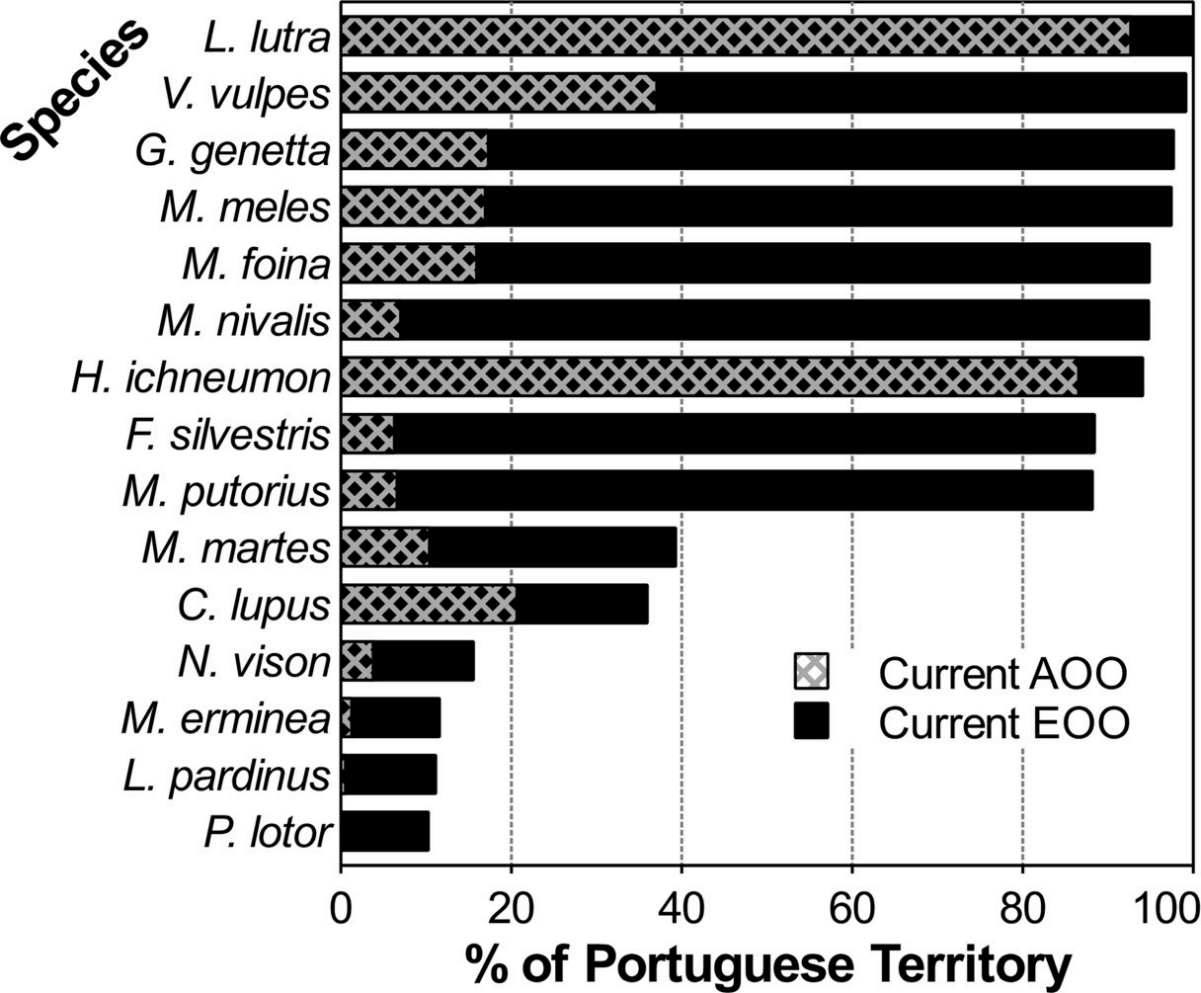
Comparison of the current Area of Occupancy (AOO) and Extent of Occurrence (EOO) for each species, given as percentages of the mainland Portuguese territory (see Methods section for details).

**Fig 6:**
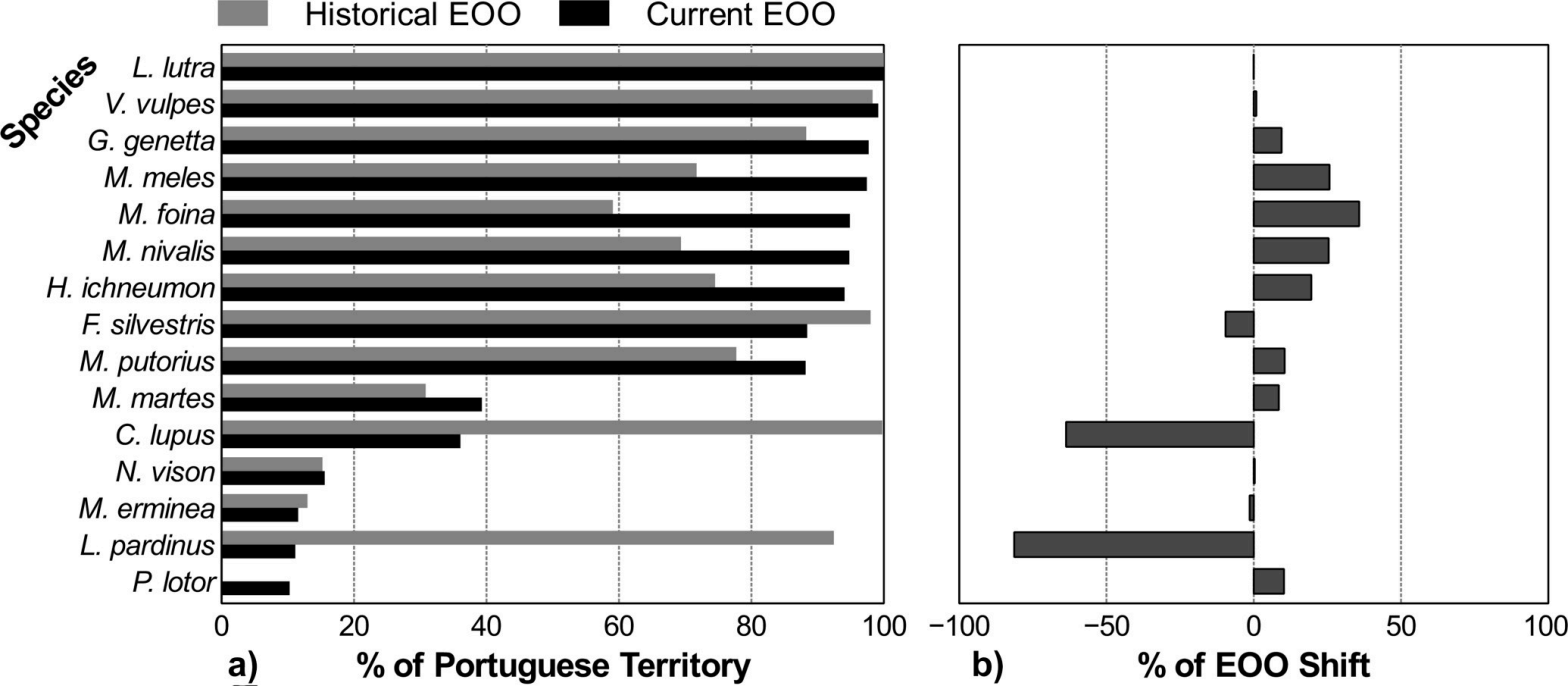
**Comparison of historical and current Extent of Occurrence (EOO) for each species, as percentages of the mainland Portuguese territory (a), and the percentage of change in EOO from historical to current times, with negative values suggesting range regression and positive values suggesting range expansion (b)**.

**Fig 7:**
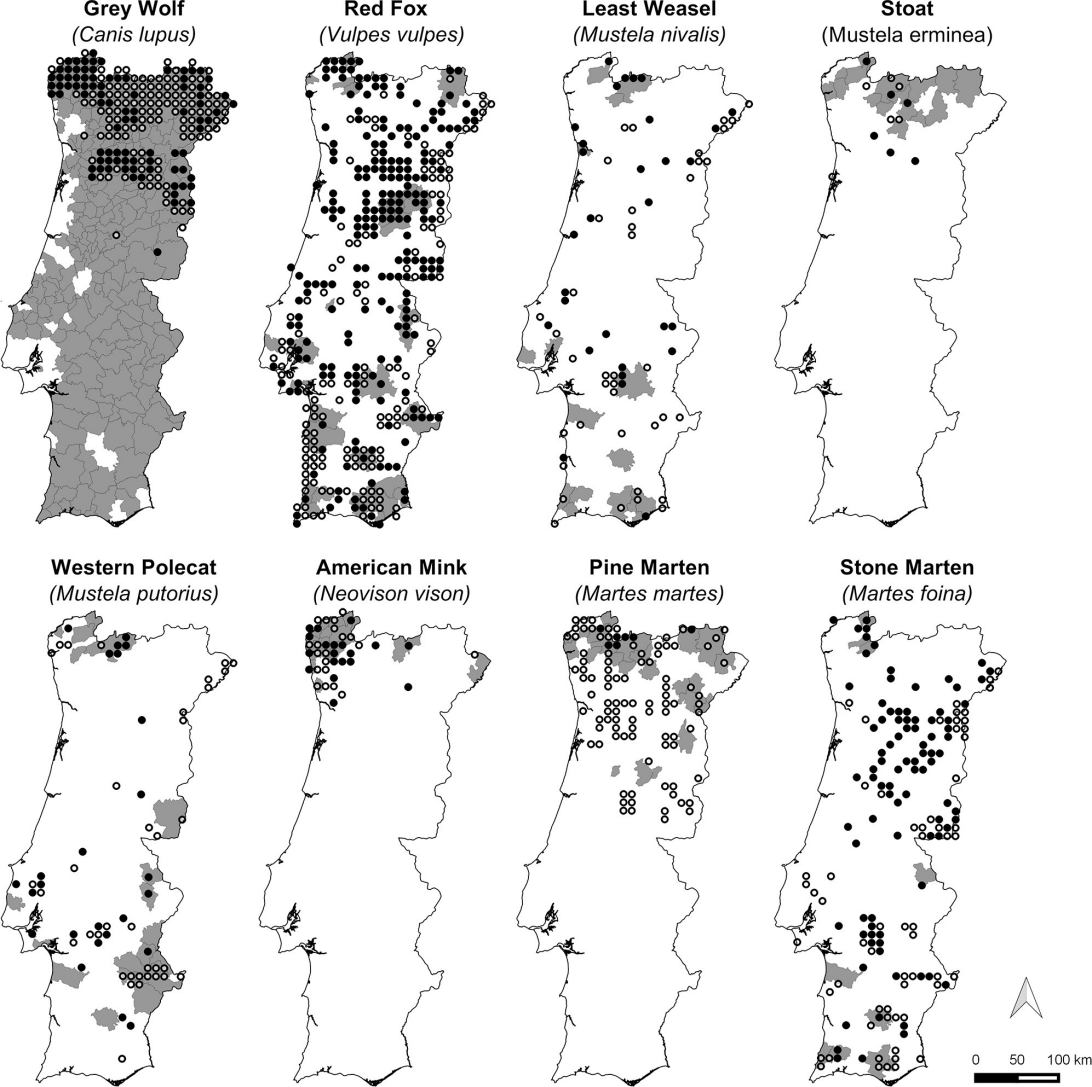
Historical (pre-1999) and current (post-2000) presence records of the grey wolf, red fox, least weasel, stoat, western polecat, American mink, pine marten and stone marten in mainland Portugal. Historical records are represented at the municipality level (Grey areas–Presence recorded; White areas–Presence not recorded); Current records are represented on UTM 10x10-km^2^ cells (Black circles–Confirmed; White circles–Unconfirmed). See Methods section for further details.

**Fig 8:**
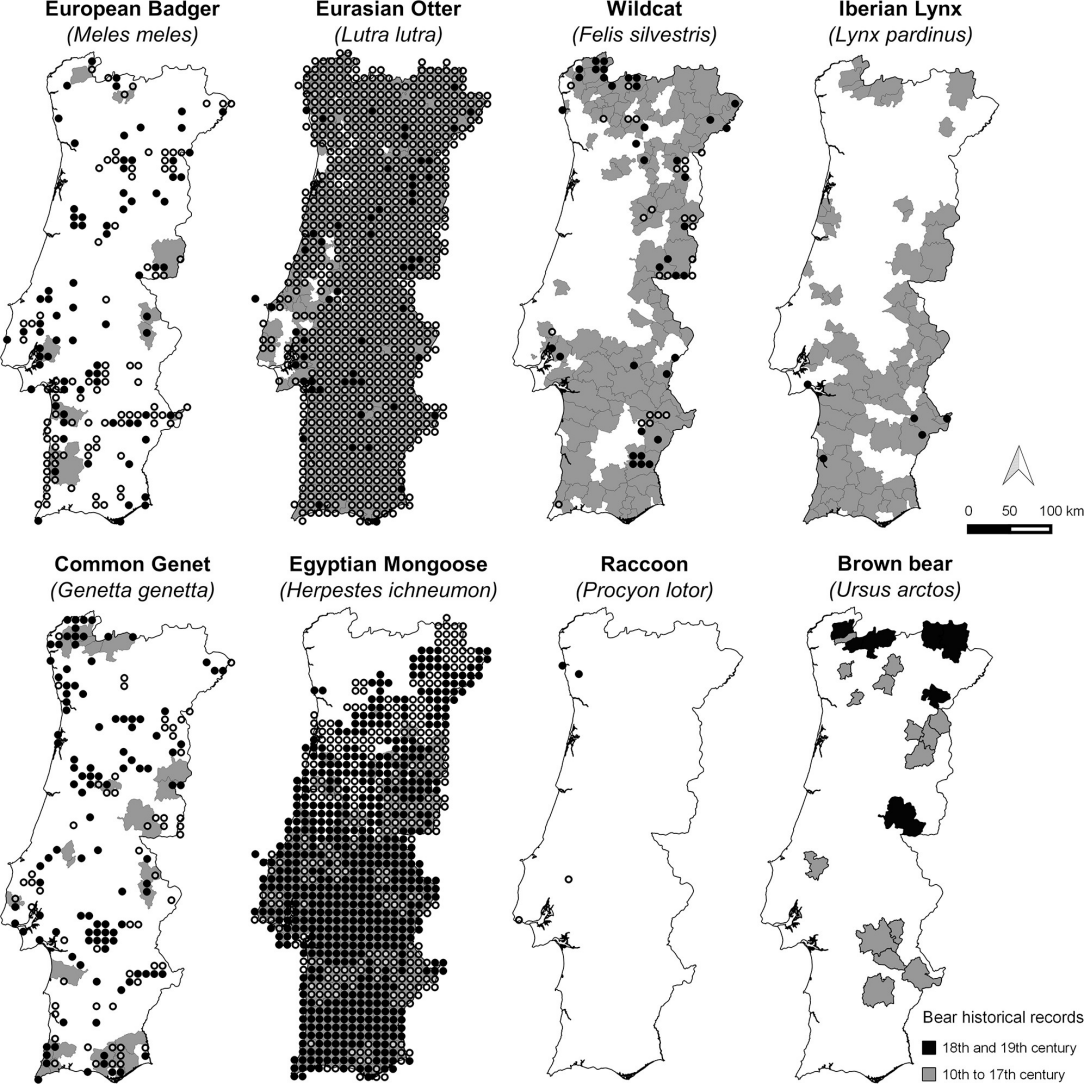
Historical (pre-1999) and current (post-2000) presence records of the European badger, Eurasian otter, wildcat, Iberian lynx, common genet, Egyptian mongoose, racoon in mainland Portugal, plus brown bear’s historical distribution during two different time periods. Historical records are represented at the municipality level (Grey areas–Presence recorded; White areas–Presence not recorded); Current records are represented on UTM 10x10-km^2^ cells (Black circles–Confirmed; White circles–Unconfirmed). See Material and methods for further details.

Regarding the current distribution range of Portuguese carnivores (Fig 5), over half the species (N = 9) is widespread in Portugal, with current EOOs covering over 85% of the country’s continental territory, whereas six species have restricted ranges, occupying less than 40% of the mainland. However, some care should be taken in interpreting these results as some EOOs may be overestimated. EOO corresponds to the area within an imaginary boundary that encompasses all known species presence data [51,52]. Thus, due to species degree of ecological specialization and landscape composition, there will be areas within EOO that will not be occupied by a specific species, since they will not meet the necessary ecological requirements that allow survival. When comparing historical and current distributions, and despite the shortcomings of this comparison (i.e. different geographical units and sampling efforts), there are differences among species in the general trends of their distributions (Figs 6–8 and Table 1).

Some carnivores that are fairly common and widely distributed in Europe (e.g. red fox, otter, badger, stone marten, weasel and polecat; [42,47]) are also so across Portugal, both in historical and current times. The two species of African origin (e.g. common genet and Egyptian mongoose) are also widespread and seem to be well adapted to the Portuguese landscapes and climate [42,47,72,73]. However, while the genet seems to have maintained its range during the last decades, the Egyptian mongoose has been undergoing an extensive, rapid expansion towards the north of the country, and is currently absent only in the north-western portion within the Eurosiberian biogeographic region [74].

For the six species that currently have more restricted distributions, the observed patterns seem to result from different factors. For the wolf and Iberian lynx, it is a result of a massive range contraction caused largely by anthropogenic factors during early 20^th^ century [47]. For the stoat and pine marten, which seem to be restricted to northern Portugal since historical times, this is probably due to biogeographical constraints, since this area is at the edge of the Eurosiberian region [75]. Finally, recent invaders such as the American mink and the raccoon have so far colonized a relatively small area in mainland Portugal [22], with the former currently expanding its range southwards and eastwards and already occupying most hydrographic basins of north Portugal.

Three species–stoat, pine marten and wildcat—show unclear trends in distribution range. This ambiguity may be related to uncertainties regarding presence data and species identification (e.g. presence signs of pine marten and wildcat may be easily confounded with those of stone marten and domestic/feral cat *Felis catus*, respectively; [76]), or to the fact that their southern range limit is not yet clear (e.g. stoat). Besides, for the pine marten, most current records came from an assessment based on questionnaires [77] which means that the actual occurrence area may be much more restricted, as suggested by the few confirmed records, given the tendency for misidentification of this species.

All carnivore species, apart from the otter and the Egyptian mongoose, present a visibly smaller AOO than EOO in Portugal (Fig 5). The small difference between the AOO and EOO for the otter and mongoose may reflect the fact that these are the only widespread species whose distributions have been thoroughly assessed in Portugal [70,74]. For the remaining species, the discrepancy between AOO and EOO seem to be either due to insufficient survey coverage for generally common and widespread species (e.g. red fox, stone marten, weasel, badger and common genet), or because certain species actually are less common and/or occur in fragmented and localized populations (e.g. polecat and wildcat; [47]).

### Insights from non-systematic data: Management and methodological implications

This study, based on the compilation of non-systematic data, allowed for an updated species checklist and the first thorough assessment of the distribution patterns of all 16 carnivores occurring in Portugal since historical times. These include 12 native species, one naturalized species, two recently introduced exotic species and one extinct species, providing baseline information essential for wildlife management both in this country and at a wider scale. The most recent invasive carnivore in Portugal, the raccoon, is becoming an important conservation problem in Europe, including in the neighbouring country, Spain, as it affects many native species through predation and/or competition [20,23]. Yet the distant location of the records collected in Portugal in relation to the known Spanish populations [23] suggests that individuals have been intentionally released or escaped from captivity, as opposed to being a part of a breeding or naturally expanding population. Thus, a targeted management program in Portugal is crucial to accurately assess this species’ current distribution, identify the origin of these individuals and ensure their timely control or eradication.

The distribution ranges suggested by non-systematic data may reflect the actual ranges for some well-studied species (e.g. grey wolf, otter, Iberian lynx, American mink and Egyptian mongoose), but for others it is apparent that the spatial coverage of surveys has been insufficient. Consequently, these species (e.g. least weasel, stoat, western polecat, pine marten, stone marten, European badger, common genet, wildcat) should be the focus of more research in the future, to allow a better grasp of their distributions at a national level and the design of adequate conservation and management strategies.

With this work, it became clear that many data exist on carnivores at a national level, but these are often scattered and frequently inaccessible to the general scientific community (and the wider public), due to a lack of compilation and centralization of available information. The fact that most of the sources identified are in Portuguese further complicates the ample access and dissemination of these data to an international audience. Online databases were an important source of species occurrence data in this study, as they do some of the work of centralizing information and making it publicly available. The downside of using online public databases is the uncertainty associated with data collected from heterogeneous sources, using different methodologies and by observers with varying levels of expertise. Thus, these data need to be critically inspected to remove dubious records, as we did in this study. Data obtained from the scientific literature are, therefore, great complementary sources, as they normally come with a description of how the records were collected, providing more thorough information. However, underlying details such as georeferenced records are not always easily obtainable directly from the literature.

Although some species, such as the badger, genet and otter, produce fairly unequivocal presence signs (e.g., distinctive footprints, scats or latrines [76], we used a conservative approach by considering them “Unconfirmed’ records, for consistency with the remaining species. For several Portuguese carnivores, most records that are classified as ‘Unconfirmed’ came from questionnaires or from presence signs without genetic validation, which may lead to an overestimation of their AOO (see Table 1). Thus, there is an urgent need for improving the accuracy of record collection for most species, by standardizing monitoring methodologies (e.g. camera-trapping or genetic confirmation of scats; [31]). Ideally, monitoring goals and techniques should align with concomitant efforts in Spain, for comparison and establishment of a cohesive Iberian network for carnivore conservation and management. This is particularly relevant for species able to cover wide ranges, such as large carnivores, where comparable information at a country level is crucial to assure an efficient transboundary management at the population level [78]. With this goal in mind, we provide as a supplement (S1 Fig) updated maps of carnivore distribution in the Iberian Peninsula, combining the data now gathered in this study with those from the latest atlas of Spanish mammals [59].

### Study relevance and future perspectives

Portugal is a good example of a highly humanized country with a marginal geographic and economic position in continental Europe, which has only recently witnessed a boost in scientific production. The country harbours a remarkably high diversity of carnivore species (among others) from different biogeographic origins, but the lack of detailed distribution and abundance information for most of them hinders adequate regional or global biodiversity planning. Thus, the assessment of carnivore research trends and species distributions in Portugal represents a model that can be transferable to future similar investigations in regions with high biodiversity, but where deficient available data, peripheral geographies, and scientific investment has been traditionally low.

Moreover, the Portuguese carnivore community is diverse in eco-morphological traits and encompasses all taxonomic families found in the Palearctic, which provides a good model to study research trends and investigate the relative scientific attention given to each species. The latter is crucial as it ultimately influences the available body of knowledge and a species conservation status, which in turn determines the amount of conservation investment made at a national and international level.

The volume, resolution and geographic extent of the data gathered in this study provide valuable information for the future assessment, conservation and management of mammalian terrestrial carnivores in Portugal, but also at a wider scale. Our study revealed some important knowledge gaps, especially concerning species classified as Data Deficient and recent invaders. Thus, it is imperative that future research should focus on the most neglected species and on less investigated topics, such as disease and genetics, to enable the design of robust species management and conservation plans. Moreover, the present compilation of non-systematic species distribution data may prompt further initiatives to gather species occurrence data (e.g. citizen-science based) and support future assessments regarding methodological standardization, as well as prioritization of sampling areas and species. Our results also provide an opportunity to expand our knowledge regarding carnivore ecology and distribution at a regional scale, namely through the development of species distribution models, to assess potential areas of occurrence and identify variables (biogeographical, environmental, topographic and anthropogenic) that shape these species’ distributions. Thus, future studies targeting a more precise evaluation of carnivore distribution patterns should focus on: i) investing in more accurate sampling methods to increase the reliability of collected data, ii) increasing the sampling effort in regions where information is lacking and, iii) ensuring a wider compilation of historical records, considering both geographic range and time periods.

## Supporting information

S1 Fig Updated Iberian distributions of the carnivore species occurring (currently or historically) in Portugal. Dots represent UTM 10x10-km cells with confirmed (black) or unconfirmed (dark red) presence records in Portugal compiled in this study (see Methods for further details), together with presence records in Spain (light blue) from the latest mammal atlas in this neighbouring country (Palomo L.J., Gisbert J. & Blanco J.C. 2007, Atlas y Libro Rojo De Los Mamíferos Terrestres De España. Dirección General para la Biodiversidad-SECEM-SECEMU, Madrid: 588 pp.
(DOCX)

S1 Table Scientific literature on mammalian terrestrial carnivores in Portugal from 1789 until November 2015 (n = 755 studies). Note: Relevant studies were identified using several search engines, including Google Scholar (http://scholar.google.com), ISI Web of Knowledge / Web of Science (WoS, www.wokinfo.com), Scientific Electronic Library Online (SciELO, www.scielo.org) and online archives of all Portuguese universities. In each database, several combinations of keywords were used to identify relevant publications for all carnivore species known to occur in Portugal: the scientific and common name (both in Portuguese and English), ‘carnivore’, ‘Portugal’ and ‘Iberian Peninsula’. Reference lists of publications were also used as bibliographic sources. ‘Research Topic’ refers to the publication’s main area of research and was defined as follows: ‘Conservation’ (studies related to human-wildlife conflicts; human perceptions and attitudes towards carnivores; illegal persecution; damages; habitat recovery; conservation action plans; impact of human activities), ‘General Ecology’ (trophic ecology; reproduction; habitat requirements and selection; home ranges; space use; activity; ecological modelling; scent-marking; behavioural responses; social ecology; abundance), ‘Genetics’ (phylogeography, population genetics, non-invasive genetics, hybridization, molecular markers), ‘Health Status’ (parasites, diseases, physiological parameters), ‘Population Status’ (past and present distribution patterns; population size; population trends and dynamics; Population Viability Analysis (PVA); monitoring) and ‘Others’ (palaeontology; ethology; systematics; morphology; anatomy; methodological approaches; etc.). Publications marked in light grey were used to obtain presence records.
(DOCX)

S1 Dataset Classification of Portuguese Municipalities for each carnivore species according to two possible categories for historical presence records (after 2000): No records (0) and with records (1). (XLSX)

S2 Dataset Classification of each UTM 10x10-km cell for each species into the three possible categories according to current presence records (after 2000): No records (0), Confirmed (C) and Unconfirmed (U) presence. (XLSX)
