# Supplementary material for: Research trends and geographical distribution of mammalian carnivores in Portugal (SW Europe)
Source: PLoS One. 2018 Nov 29;13(11):e0207866. doi: 10.1371/journal.pone.0207866 (PMC6264823; doi:10.1371/journal.pone.0207866)

**S1 Fig. Updated Iberian distributions of the carnivore species occurring (currently or historically) in Portugal**. Dots represent UTM 10x10-km cells with confirmed (black) or unconfirmed (dark red) presence records in Portugal compiled in this study (see Methods for further details), together with presence records in Spain (light blue) from the latest mammal atlas in this neighbouring country (Palomo L.J., Gisbert J. & Blanco J.C. 2007, Atlas y Libro Rojo De Los Mamíferos Terrestres De España. Dirección General para la Biodiversidad-SECEM-SECEMU, Madrid: 588 pp.).


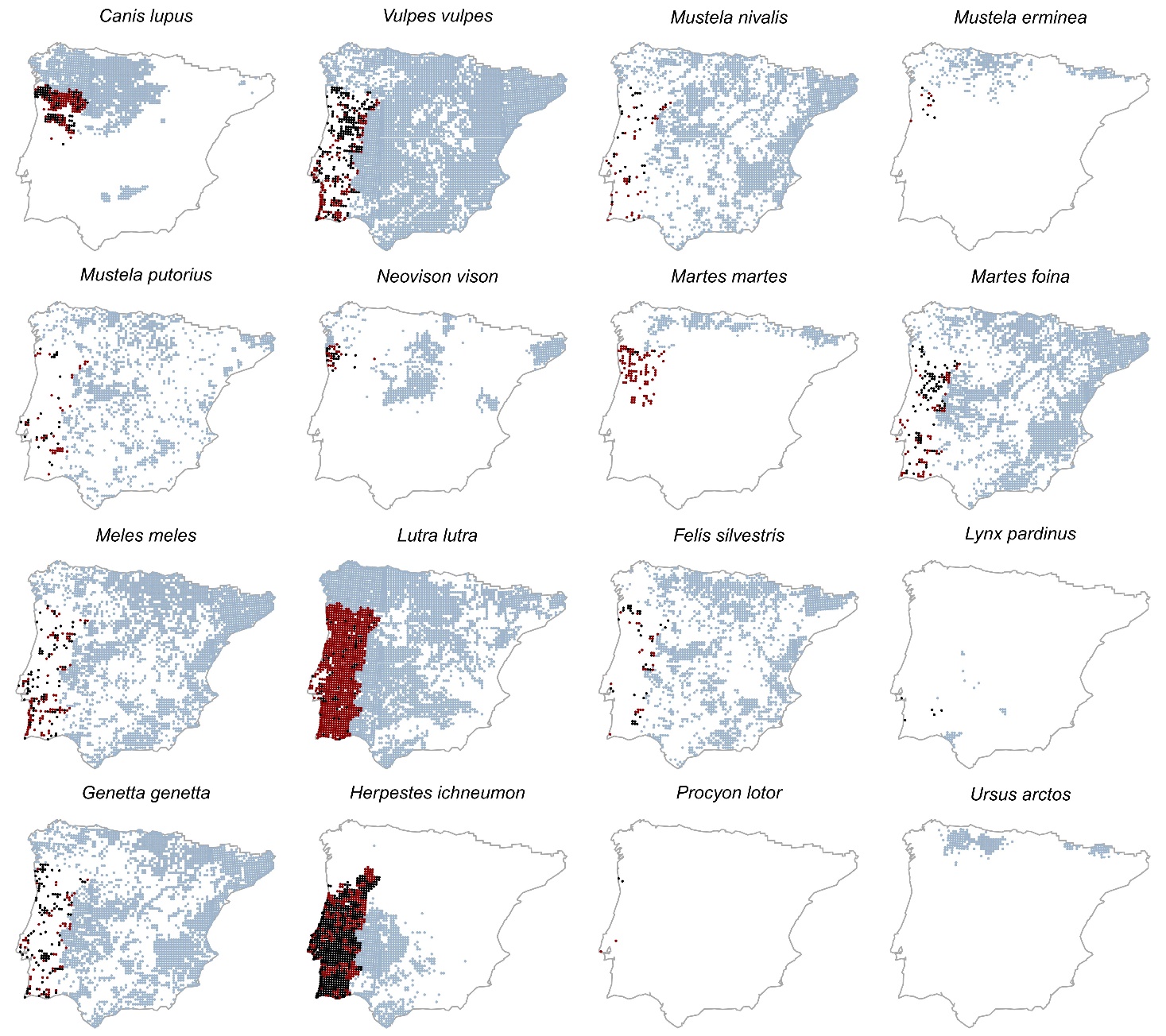

Supplement: S1 Fig — Dots represent UTM 10x10-km cells with confirmed (black) or unconfirmed (dark red) presence records in Portugal compiled in this study (see Methods for further details), together with presence records in Spain (light blue) from the latest mammal atlas in this neighbouring country (Palomo L.J., Gisbert J. & Blanco J.C. 2007, Atlas y Libro Rojo De Los Mamíferos Terrestres De España. Dirección General para la Biodiversidad-SECEM-SECEMU, Madrid: 588 pp. (DOCX) [file pone.0207866.s001.docx]
