# Supplementary material for: Research trends and geographical distribution of mammalian carnivores in Portugal (SW Europe)
Source: PLoS One. 2018 Nov 29;13(11):e0207866. doi: 10.1371/journal.pone.0207866 (PMC6264823; doi:10.1371/journal.pone.0207866)
Supplement: S1 Table — Note: Relevant studies were identified using several search engines, including Google Scholar (http://scholar.google.com), ISI Web of Knowledge / Web of Science (WoS, www.wokinfo.com), Scientific Electronic Library Online (SciELO, www.scielo.org) and online archives of all Portuguese universities. In each database, several combinations of keywords were used to identify relevant publications for all carnivore species known to occur in Portugal: the scientific and common name (both in Portuguese and English), ‘carnivore’, ‘Portugal’ and ‘Iberian Peninsula’. Reference lists of publications were also used as bibliographic sources. ‘Research Topic’ refers to the publication’s main area of research and was defined as follows: ‘Conservation’ (studies related to human-wildlife conflicts; human perceptions and attitudes towards carnivores; illegal persecution; damages; habitat recovery; conservation action plans; impact of human activities), ‘General Ecology’ (trophic ecology; reproduction; habitat requirements and selection; home ranges; space use; activity; ecological modelling; scent-marking; behavioural responses; social ecology; abundance), ‘Genetics’ (phylogeography, population genetics, non-invasive genetics, hybridization, molecular markers), ‘Health Status’ (parasites, diseases, physiological parameters), ‘Population Status’ (past and present distribution patterns; population size; population trends and dynamics; Population Viability Analysis (PVA); monitoring) and ‘Others’ (palaeontology; ethology; systematics; morphology; anatomy; methodological approaches; etc.). Publications marked in light grey were used to obtain presence records. (DOCX) [file pone.0207866.s002.docx]

**S1 Table. Scientific literature on mammalian terrestrial carnivores in Portugal from 1789 until November 2015 (n=755 studies).** Note: Relevant studies were identified using several search engines, including Google Scholar (http://scholar.google.com), ISI Web of Knowledge / Web of Science (WoS, www.wokinfo.com), Scientific Electronic Library Online (SciELO, www.scielo.org) and online archives of all Portuguese universities. In each database, several combinations of keywords were used to identify relevant publications for all carnivore species known to occur in Portugal: the scientific and common name (both in Portuguese and English), ‘carnivore’, ‘Portugal’ and ‘Iberian Peninsula’. Reference lists of publications were also used as bibliographic sources. ‘Research Topic’ refers to the publication’s main area of research and was defined as follows: ‘Conservation’ (studies related to human-wildlife conflicts; human perceptions and attitudes towards carnivores; illegal persecution; damages; habitat recovery; conservation action plans; impact of human activities), ‘General Ecology’ (trophic ecology; reproduction; habitat requirements and selection; home ranges; space use; activity; ecological modelling; scent-marking; behavioural responses; social ecology; abundance), ‘Genetics’ (phylogeography, population genetics, non-invasive genetics, hybridization, molecular markers), ‘Health Status’ (parasites, diseases, physiological parameters), ‘Population Status’ (past and present distribution patterns; population size; population trends and dynamics; Population Viability Analysis (PVA); monitoring) and ‘Others’ (palaeontology; ethology; systematics; morphology; anatomy; methodological approaches; etc.). Publications marked in light grey were used to obtain presence records.

| **Publication name** | | **Type of Publication** | | **Research Topic** | |
| --- | --- | --- | --- | --- | --- |
| Abreu F, Galhardo L (2012) Effects of feeding enrichment in behavior of a female otter in captivity. In: *Abstracts of the IV Congresso da Fauna Selvagem WAVES Portugal*, 75. Bragança, Portugal. | | Conference Proceedings | | General ecology | |
| Abreu MP (1993) *A comunidade de carnívoros da Reserva Natural da Serra da Malcata. Uma partilha de recursos.* Faculty of Sciences of the University of Lisbon, Lisbon, Portugal. | | Thesis | | General ecology | |
| Abreu P (2003) *O Lince-ibérico na nossa Região: um Projecto Interdisciplinar.* Instituto da Conservação da Natureza. | | Book | | Conservation | |
| Afonso MR (1997) *Abordagem ao estudo do grau de contaminação por mercúrio da bacia do Rio Tejo e avaliação do possível impacte na população de lontra (Lutra lutra L., 1758).* Final degree thesis, Faculty of Sciences of the University of Lisbon, Lisbon, Portugal. | | Thesis | | Conservation | |
| Alexandre AS (1995) *A raposa (Vulpes vulpes silacea Miller, 1907) na região de Onor no Parque Natural de Montesinho: Ecologia trófica, uso do espaço e uso do tempo.* Final Degree thesis, Faculty of Sciences of the University of Lisbon, Lisbon, Portugal. | | Thesis | | General ecology | |
| Alexandre AS, Cândido AT, Grilo C, Moço G, Petrucci-Fonseca F (1999) Nuevos datos sobre la población lupina portuguesa a sur del Rio Duero. In: *Abstracts of the Congresso sobre Recuperación del Lobo en Sierra Morena*, Sevilla, Spain. | | Conference Proceedings | | General ecology | |
| Alexandre AS, Cândido AT, Grilo C, Moço G, Petrucci-Fonseca F (1999) Propuesta de conservación del Lobo al sur del río Duero en Portugal: definición de corredores ecológicos. In: *Resúmenes IV Jornadas de la SECEM*, 4. Segovia, Spain. | | Conference Proceedings | | Conservation | |
| Alexandre AS, Cândido AT, Petrucci-Fonseca F (2000) A população lupina portuguesa a sul do Rio Douro. *Galemys* 12: 113-122. | | non-SCI Journals | | Population Status | |
| Alfonso-Roque MM (1991) Introdução de animais na ilha de S. Miguel (Açores) e eventuais consequências na helmintofauna de vertebrados terrestres. *Garcia de Orta - Série de Zoologia* 18: 5–12. | | non-SCI Journals | | Health status | |
| Almaça C (1977) A lontra, Lutra lutra, no litoral marítimo. *Boletim da LPN* 16: 17–19. | | non-SCI Journals | | Population Status | |
| Alonso P, Villar Pías J, Pérez Hernández T, Sánchez Quintas M (2012) Evolução recente da população de lobos presente a SE da Galiza. Apontamentos sobre reprodução e mortalidade. In: *Abstracts of the III Iberian Wolf Congress*, 39. Lugo, Spain. | | Conference Proceedings | | Population Status | |
| Álvares F (1995) *Aspectos da distribuição e ecologia do lobo no noroeste de Portugal. O caso do Parque Nacional da Peneda-Gerês.* Final Degree thesis, Faculty of Sciences of the University of Lisbon Lisbon, Portugal. | | Thesis | | Population Status | |
| Álvares F (1997) Novos dados sobre a fauna de vertebrados terrestres no Noroeste de Portugal. In: *I Encontro do Norte: Ambiente, Desenvolvimento, Autarquias e Educação*, Braga, Portugal. | | Conference Proceedings | | Population Status | |
| Álvares F (1997) O Lince-ibérico (Lynx pardinus) no Noroeste de Portugal - Presença Historica e Situação Actual. Instituto da Conservação da Natureza/Programa Liberne. | | Technical report | | Population Status | |
| Álvares F (1998) Consequências da Auto-Estrada Braga-Valença na população lupina do extremo Noroeste de Portugal. *Boletim informativo do Grupo Lobo* 13: 1–2. | | non-SCI Journals | | Conservation | |
| Álvares F (1999) Andam a soltar lobos?! *Boletim informativo do Grupo Lobo* 14: 1–3. | | non-SCI Journals | | Conservation | |
| Álvares F (1999) Implicações da utilização de veneno direccionado ao lobo na conservação dos ecossistemas. O exemplo do Noroeste de Portugal. In: *Seminário “O envenenamento e o seu efeito na conservação das espécies selvagens e em especial nas aves”,* Mértola, Portugal. | | Conference Proceedings | | Conservation | |
| Álvares F (2000) A realidade e o Mito do lobo. In: *Abstracts of the Ciclo de Conferências “O Despertar da Primavera,”* Universidade de Trás-os Montes e Alto Douro, Vila Real, Portugal. | | Conference Proceedings | | Conservation | |
| Álvares F (2000) O lobo em Portugal. Situação actual e causas de regressão. In: *Abstracts of the Ciclo de Colóquios 2000/2001: O Lobo na Beira Alta – Sobrevivência ou Extinção*, Guarda, Portugal. | | Conference Proceedings | | Population Status | |
| Álvares F (2000) O Lobo no Noroeste de Portugal, a vida de um sobrevivente. *Tribuna da Natureza* 1: 4–7. | | non-SCI Journals | | Population Status | |
| Álvares F (2001) O Lobo - Mitos e Realidades. In: *Abstract of the I Jornadas Ambientais do Alto Tâmega e Verín*, Chaves, Portugal. | | Conference Proceedings | | Conservation | |
| Álvares F (2003) *A Problemática dos Venenos na Conservação do Lobo e o seu Impacto na Biodiversidade dos Ecossistemas*. Programa Antídoto - Portugal, Lisbon, Portugal. | | Technical report | | Conservation | |
| Álvares F (2003) *Monitorização do lobo (Canis lupus) na área de influência do Parque Nacional Peneda.-Gerês: análise do decénio 1994-2003. Relatório Anual de Projecto.* Lisbon, Portugal. | | Technical report | | Population Status | |
| Álvares F (2004) O lobo no imaginário popular. In: Nunes M (ed) *Serra da Aboboreira: a Terra, o Homem e os Lobos*, 135–145. Câmara Municipal de Amarante. | | Book | | Conservation | |
| Álvares F (2004) O lobo-ibérico, biologia, ecologia e distribuição. In: Nunes M (ed) *Serra da Aboboreira: a Terra, o Homem e os Lobos*, 85–91. Câmara Municipal de Amarante. | | Book | | General ecology | |
| Álvares F (2004) Status and Conservation of the Iberian Wolf in Portugal. *Wolf Print* 20: 4–6. | | non-SCI Journals | | Population Status | |
| Álvares F (2005) *Casos Particulares de Conservação de Carnívoros em Portugal - O Lobo-ibérico, o Gato-bravo e a Marta*. ALDEIA | | Technical report | | Conservation | |
| Álvares F (2005) *O Mito do lobo: a importância de um grande predador na cultura das comunidades rurais.* Terranatur | | non-SCI Journals | | Conservation | |
| Álvares F (2006) Espécies Emblemáticas and Desenvolvimento Rural: o potencial do lobo-ibérico e da sua identidade na cultura popular. In: *Actas das Jornadas sobre Biodiversidade e Mundo Rural: Perspectivas e Estratégias de Conservação da Fauna Selvagem*, ALDEIA/NEBUP. | | Conference Proceedings | | Conservation | |
| Álvares F (2011) *Ecologia e conservação do lobo (Canis lupus, L.) no noroeste de Portugal. PhD thesis, Faculty of Sciences of the University of Lisbon, Lisbon, Portugal.* | | Thesis | | Conservation | |
| Álvares F (2013) Dinâmica das populações de lobo em ambientes humanizados *Colóquio “O lobo ibérico na Beira Interior”, ATN/Grupo Lobo, Almeida, Portugal, 16 March 2013. Oral.* | | Conference Proceedings | | Conservation | |
| Álvares F (2013) Etnobiologia: a utilização do lobo na medicina tradicional. In: Rodrigues JB (ed) *Entre Lobos & Pastores*, 90–92. CORANE/ALDEIA, Bragança, Portugal. | | Book | | Conservation | |
| Álvares F (2013) Meios e procedimentos na luta contra um predador. In: Rodrigues JB (ed) *Entre Lobos & Pastores*, 64–66. CORANE/ALDEIA, Bragança, Portugal. | | Book | | Conservation | |
| Álvares F (2013) Wolf conservation in human-dominated landscapes: ecological and socio-economic constraints. In: *Seminar Series “Colloquium for Applied Ecology and Planning”, Technical University Munich, Germany, 2013*, Oral. | | Conference Proceedings | | Conservation | |
| Álvares F, Alonso P, Sierra P, Petrucci-Fonseca F (2000) Os fojos dos lobos na Península Ibérica. Sua inventariação, caracterização e conservação. *Galemys* 12: 57–77. | | non-SCI Journals | | Conservation | |
| Álvares F, Brito JC (2006) Habitat requirements and potential areas of occurrence for the Pine Marten in North-western Portugal: conservation implications. In: Santos-Reis M, Birks JDS, O’Doherty EC, Proulx G (eds) *Martes in Carnivore Communities: Proceedings of the Fourth International Martes Symposium*, 29–45. Alpha Wildlife Publications, Alberta, Canada. | | Book | | Population Status | |
| Álvares F, Domingues J (2010) Presença histórica do urso em Portugal e testemunhos da sua relação com as comunidades rurais. *AÇAFA on-line*: 1–22. | | non-SCI Journals | | Others | |
| Álvares F, Domingues J, Sierra P, Primavera P (2011) Cultural dimension of wolves in the Iberian Peninsula: implications of ethnozoology in conservation biology. *Innovation: The European Journal of Social Science Research* 24: 313–331. | | non-SCI Journals | | Conservation | |
| Álvares F, Pereira E, Petrucci-Fonseca F (2000) O lobo no Parque Internacional Gerês-Xurés. Situaçao populacional, aspectos ecológicos e perspectivas de conservação. *Galemys* 12: 223–239. | | non-SCI Journals | | Conservation | |
| Álvares F, Petrucci-Fonseca F (1996) O lobo no Parque Nacional da Peneda-Gerês - Ecologia e Distribuição. In: *Abstracts of the I Encontro Ecologista sobre o Lobo Ibérico*, Benavente, Zamora, Espanha. | | Conference Proceedings | | General ecology | |
| Álvares F, Petrucci-Fonseca F (1997) O lobo no Alto Minho - Problemática da conservação de um predador numa região fortemente humanizada. In: *Abstracts of the I Encontro Regional do Norte - Ambiente, Desenvolvimento, Autarquias e Educação*, 47–48. Associação Portuguesa de Biólogos, Braga, Portugal,. | | Conference Proceedings | | Conservation | |
| Álvares F, Petrucci-Fonseca F (2001) Social and Ecological Dimemnsions of Human-Wolf Conflict in Northwest Portugal. In: *Abstracts of the 8th International Theriological Congress*, Sun City, South Africa. | | Conference Proceedings | | Conservation | |
| Álvares F, Petrucci-Fonseca F (2002) O papel do Ecoturismo e da Educação Ambiental na conservação de espécies ameaçadas - O caso do lobo-ibérico no Parque Nacional da Peneda-Gerês. In: *Proceedings from Congresso Nacional Desenvolvimento Sustentável em Áreas de Montanha*, PNPG, Terras de Bouro, Portugal. | | Conference Proceedings | | Conservation | |
| Álvares F, Petrucci-Fonseca F (2012) Ecologia trófica e impacto da predação do lobo num sistema com várias espécies de ungulados domésticos. In: *Abstracts of the III Iberian Wolf Congress*, 31. Lugo, Spain. | | Conference Proceedings | | General ecology | |
| Álvares F, Primavera P (2004) The Wolf in rural communities’ culture in the North of Portugal. *Wolf Print* 20: 10–12. | | non-SCI Journals | | Conservation | |
| Álvares F, Rio-Maior H, Nakamura M, Petrucci-Fonseca F, Godinho R (2012) Parâmetros populacionais do lobo no noroeste de Portugal: padrões espácio-temporais e considerações metodológicas. In: *Abstracts of the III Iberian Wolf Congress*, 33. Lugo, Spain. | | Conference Proceedings | | Population Status | |
| Álvares F, Roque S, Petrucci-Fonseca F (2013) Wolf feeding strategies by wolves in agricultural landscapes: lessons from two areas in Portugal. In: Potocnik H, Razen N, Mulej J, Jelencic M, Bertoncelj I (eds) *Abstracts of the International Conference “Wolf Conservation in Human Dominated Landscapes”, 25-28 September 2013*, Oral. University of Ljubljana, Biotechnical Faculty, Postojna, Slovenia. | | Conference Proceedings | | General ecology | |
| Álvares F, Sierra P (2004) *Monitorização do lobo (Canis lupus) na área de influência do Parque Nacional da Peneda-Gerês. Relatório Anual de Projecto.* Grupo Lobo/PNPG. | | Technical report | | Population Status | |
| Álvares F, Simão R, Alexandre AS, Cândido AT, Petrucci-Fonseca F (1998) Wolf monitoring in Portugal with the selection of key-sites. Importance for the definition of a conservation strategy. In: Reig S (ed) *Abstracts of the Euro-American Mammal Congress*, 262. Santiago Compostela, Spain. | | Conference Proceedings | | Conservation | |
| Alves AF, Loureiro F, Rosalino LM, Carvalho S, Rei C, Santos-Reis M (2007) Effects of fire on Eurasian badger’s trophic ecology in cork oak woodlands of SW Portugal. *Galemys* 19: 251–270. | | non-SCI Journals | | General ecology | |
| Álvaro C,Bekker H, Bekker J, Boshamer J, Buys J, Hunia A, Kosten E et al. (2004) Mammal Survey Alvão Natural Park (Portugal). Society for the Study and Conservation of Mammals, Nijmegen, Netherlands. | | Technical report | | Population Status | |
| Alves J (2014) *Avaliação da resposta de lince-ibérico (Lynx pardinus, Temminck 1827) à presence humana, face a diferentes métodos de preparação para reintrodução.* MSc thesis, Faculty of Sciences of the University of Lisbon, Lisbon, Portugal. | | Thesis | | Conservation | |
| Alves JP (2001) The Relationships between Humans and Wolves in the Montesinho Natural Park (North-east of Portugal): From Destruction to Conservation. In: *Abstracts of the Canid Biology & Conservation: An International Conference*, 26. Oxford, U.K. | | Conference Proceedings | | Conservation | |
| Andrade J, Pinho R (1959) Situation du Lynx au Portugal. In: *Conseil international de la Chasse - VIII Assemblée Generale*, 131–132. Vienne, France. | | Conference Proceedings | | Population Status | |
| Ângelo IS (2000) *Aspectos da ecologia da raposa (Vulpes vulpes) no Nordeste Algarvio.* Final Degree thesis, Faculty of Sciences of the University of Lisbon Lisbon, Portugal. | | Thesis | | General ecology | |
| Anonymous (2005) *Plano de ordenamento da Albufeira da Aguieira*. Direção de Serviços de Utilizações do Domínio Hídrico. | | Technical report | | Population Status | |
| Antunes MT (1993) Mamíferos em Portugal: extinções desde o Plistocénico Superior. *Liberne* 43/44: 6–10. | | non-SCI Journals | | Others | |
| Antunes P, Santos R, Gomes J, Madruga L (2006) Development of participatory conflict reconciliation processes: the case of biodiversity conservation and aquaculture in the Sado estuary (Portugal). In: *Abstracts of the Society for Conservation Biology Annual Meeting*, 2. San Jose, USA. | | Conference Proceedings | | Conservation | |
| Ascensão F (2001) *Mortalidade de Vertebrados por Atropelamento em Estradas do Alto Alentejo.* Faculty of Sciences of the University of Lisbon, Lisbon, Portugal. | | Thesis | | Conservation | |
| Ascensão F (2013) *Ecologically scaled responses of forest-dwelling vertebrates to habitat fragmentation.* PhD thesis, Faculty of Sciences of the University of Lisbon, Lisbon, Portugal. | | Thesis | | Conservation | |
| Ascensão F, Grilo C, LaPoint S, Tracey J, Clevenger AP, Santos-Reis M (2014) Inter-individual variability of stone marten behavioral responses to a highway. *PLoS ONE* 9: e103544. | | SCI Journals | | Conservation | |
| Ascensão F, Grilo C, Santos-Reis M (2007) Assessing the Stone Marten’s Patch Occupancy in Fragmented landscapes and its Relation to Road-Killing Occurences. In: Proceedings of the *The 2007 International Conference on Ecology and Transportation*, 583. Little Rock, Arkansas, U.S.A. | | Conference Proceedings | | Conservation | |
| Ascensão F, Mira A (2006) Factors affecting culvert use by vertebrates along two stretches of road in southern Portugal. *Ecological Research* 22: 57–66. | | SCI Journals | | Conservation | |
| Ascensão F, Mira A (2006) *Impactes das vias rodoviárias na fauna silvestre*. University of Évora, Évora, Portugal. | | Technical report | | Conservation | |
| Ascensão F, Tracey J, Pinheiro P, Costa M, Matos H, Dias F, Clevenger A, Santos-Reis M, Grilo C (2009) ¿Actúan las autopistas como barreras para los movimientos de la garduña (Martes foina)? Sí, y más de lo que podría parecer. In: *Resúmenes IX Jornadas de la SECEM*, Bilbao, Spain. | | Conference Proceedings | | Conservation | |
| Azeda C (2001) *Análise dos factores que condicionam a distribuição da geneta (Genetta genetta Linnaeus, 1758) e do sacarrabos (Herpestes icnheumon Linnaeus, 1758) na região de influência directa das futuras albufeiras de Alqueva e Pedrógão.* Faculty of Sciences of the University of Lisbon, Lisbon, Portugal. | | Thesis | | Population Status | |
| Azinheira C (1998) *Ecologia da Lontra (Lutra Lutra L., 1758): Selecção de Habitat.* Final degree thesis, University of Évora, Évora, Portugal. | | Thesis | | General ecology | |
| Baeta Neves CM (1967) Sobre a Existência e Extinção do Urso em Portugal. *Publicações da Liga para a Protecção da Natureza*: 3–7. | | non-SCI Journals | | Population Status | |
| Balestrieri A, Remonti L, Ruiz-González A, Zenato M, Gazzola A, Vergara M et al. (2015) Distribution and habitat use by pine marten Martes martes in a riparian corridor crossing intensively cultivated lowlands. *Ecological Research* 30: 153–162. | | SCI Journals | | Population Status | |
| Baptista AC (2013) *Aplicabilidade da análise isotópica na compreensão da variação sazonal e espacial da dieta da raposa (Vulpes vulpes) num habitat Mediterrânico.* MSc thesis, Faculty of Sciences of the University of Lisbon, Lisbon, Portugal. | | Thesis | | General ecology | |
| Baptista MD (1789) *Faunae Conimbricencis Rudimentum.* Coimbra, Portugal. | | Book | | Population Status | |
| Barbosa AM (2001) *Modelação biogeográfica da distribuição da lontra (Lutra lutra) na península Ibérica com base em factores espaciais, ambientais e humanos.* Final Degree thesis, University of Malaga and University of Porto. | | Thesis | | General ecology | |
| Barbosa AM, Puerto MA, Figueiredo D, Real R (2012) Modelling disjunct carnivore distributions: the case of the wolf (Canis lupus) in the Iberian Peninsula. In: Álvares FI , Mata GE (eds) *Carnivores: Species, Conservation, and Management*, 119–137. Nova Science Publishers, Inc., Hauppauge (NY), USA. | | Book | | Population Status | |
| Barbosa AM, Real R (2010) Áreas favoráveis e factores a ter em conta na reintrodução do lince em Portugal. In: *Conferência Conservação de Vertebrados Terrestres Ameaçados em Portugal: Situação Actual e Perspectivas, 2010*, (Poster). | | Conference Proceedings | | Conservation | |
| Barbosa AM, Real R (2010) Favourable areas for expansion and reintroduction of Iberian lynx accounting for distribution trends and genetic diversity of the European rabbit. *Wildlife Biology in Practice* 6: 34–47. | | non-SCI Journals | | Conservation | |
| Barbosa AM, Real R, Olivero J, Vargas JM (2003) Otter (Lutra lutra) distribution modeling at two resolution scales suited to conservation planning in the Iberian Peninsula. *Biological Conservation* 114: 377–387. | | SCI Journals | | General ecology | |
| Barbosa AM, Real R, Vargas JM (2010) Use of coarse-resolution models of species’ distributions to guide local conservation inferences. *Conservation Biology* 24: 1378–1387. | | SCI Journals | | Conservation | |
| Barbosa M, Santos P, Cardoso H, Rebelo A, Moço G (2009) Pine Marten Density and Home Ranges in Albergaria National Forest (Portugal). In: *Abstracts of the 27th Mustelid Coloquium*, 43. Lisbon, Portugal. | | Conference Proceedings | | General ecology | |
| Barreto D (2009) *Abordagem ao impacto das auto-estradas A24 e A7 na população lupina do distrito de Vila Real.*  MSc thesis, Faculty of Sciences of the University of Lisbon, Lisbon, Portugal. | | Thesis | | Conservation | |
| Barrett-Hamilton GEH (1904) Note on an undescribed weasel from the Atlas Mountains, and on the occurrence of a weasel in the Azores. *Annals and Magazine of Natural History* 7: 323–325. | | non-SCI Journals | | Population Status | |
| Barrinha C (2001) *Análise da dieta da lontra (Lutra lutra Linnaeus, 1758) na área de regolfo de Alqueva e Pedrógão (SE Portugal).* Final Degree thesis, Faculty of Sciences of the University of Lisbon Lisbon, Portugal. | | Thesis | | General ecology | |
| Barros T, Carvalho J, Pereira MJR, Ferreira JP, Fonseca C (2015) Following The Trail: Factors Underlying the Sudden Expansion of the Egyptian Mongoose (Herpestes ichneumon) in Portugal. *PloS one* 10: e0133768. | | SCI Journals | | Population Status | |
| Barros T, Fonseca C (2009) Estatus y distribución actual del meloncillo (Herpestes ichneumon) en Portugal. In: *Resúmenes IX Jornadas de la SECEM*, Bilbao, Spain. | | Conference Proceedings | | Population Status | |
| Barros T, Fonseca C (2011) Expansão do sacarrabos Herpestes ichneumon (Linnaeus, 1758) em Portugal. *Galemys* 23: 9–15. | | non-SCI Journals | | Population Status | |
| Barros T, Gaubert P, Rocha RG, Bandeira V, Souto L, Mira A, Fonseca C (2015) Mitochondrial demographic history of the Egyptian mongoose (Herpestes ichneumon), an expanding carnivore in the Iberian Peninsula. *Mammalian Biology - Zeitschrift für Säugetierkunde*. | | SCI Journals | | Genetics | |
| Barroso I, Carmo P (1998) Conservation of the wolf in Portugal: an administration point of view. In: Reig S (ed) *Abstracts of the Euro-American Mammal Congress*, 268. Santiago Compostela, Spain. | | Conference Proceedings | | Conservation | |
| Barroso I, Llaneza L (2005) Wolf status and conservation in the Iberian Peninsula. In: *Abstracts of the Frontiers of Wolf Recovery Conference*, 26–27. International Wolf Center, Colorado Springs, U.S.A. | | Conference Proceedings | | Conservation | |
| Barroso I, Pimenta V (2008) *Sistema de Monitorização de Lobos Mortos: Relatorio de Actividades 1999-2008*. Instituto da Conservação da Natureza. | | Technical report | | Population Status | |
| Barroso I, Pimenta V, Santos N, Pimenta C, Godinho R (2012) Sistema de monitorização de lobos mortos em Portugal: resultados entre 1999 e 2011. In: *Abstracts of the III Iberian Wolf Congress*, 37. Lugo, Spain. | | Conference Proceedings | | Population Status | |
| Basto MP (2015) *Population and landscape genetics of the stone marten and red fox in Portugal: implications for conservation management of common carnivores*. PhD thesis, Faculty of Sciences of the University of Lisbon, Lisbon, Portugal. | | Thesis | | Genetics | |
| Basto MP, Matos H, Pinheiro P, Santos-Reis M (2007) Diet of stone martens (Martes foina) in the Iberian Peninsula: a comparative analysis. In: Hajkova P, Rüziczová O (eds) Abstracts of the 25th Mustelid Colloquium, 53. Trebon, Czech Republic. | | Conference Proceedings | | General ecology | |
| Basto MP, Mira A, Pedroso NM, Santos-Reis M (2005) Use of small and medium-sized reservoirs by otters in a Mediterranean area in the South of Portugal (Monfurado Natura 2000 Site). In: Abstracts of the European Otter Workshop, 30. Padula, Salermo, Italy. | | Conference Proceedings | | General ecology | |
| Basto MP, Rodrigues M, Santos-Reis M, Bruford MW, Fernandes CA (2010) Isolation and characterization of 13 tetranucleotide microsatellite loci in the Stone marten (Martes foina). *Conservation Genetics Resources* 2: 317–319. | | SCI Journals | | Genetics | |
| Bastos T (2001) *Estudo da ecologia de duas alcateias pertencentes à população lupina a sul do rio Douro. Final Degree thesis, Faculty of Sciences of the University of Lisbon Lisbon, Portugal.* | | Thesis | | General ecology | |
| Beja P (1989) Coastal otters in southwest Portugal. IUCN Otter Specialist Group Bulletin 4: 2–7. | | non-SCI Journals | | Population Status | |
| Beja P (1991) Diet of Otters (Lutra-lutra) in closely associated fresh-water, brackish and marine habitats in south-west Portugal. Journal of Zoology 225: 141–152. | | SCI Journals | | General ecology | |
| Beja P (1992) Effects of freshwater availability on the summer distribution of Otters Lutra lutra in the southwest coast of Portugal. Ecography 15: 273–278. | | SCI Journals | | General ecology | |
| Beja P (1995) *Patterns of availability and use of resources by otters (Lutra lutra L.) in Southwest Portugal.* PhD thesis, University of Aberdeen, Aberdeen, Scotland. | | Thesis | | General ecology | |
| Beja P (1995) Structure and seasonal fluctuations of rocky littoral fish assemblages in south-western Portugal – implications for Otter prey availability. Journal of the Marine Biological Association of the United Kingdom 75: 833–847. | | SCI Journals | | General ecology | |
| Beja P (1996) An analysis of otter Lutra lutra predation on introduced american crayfish Procambarus clarkii in Iberian streams. Journal of Applied Ecology 33: 1156–1170. | | SCI Journals | | Conservation | |
| Beja P (1996) Seasonal breeding and food resources of otters, Lutra lutra (Carnivora, Mustelidae), in south-west Portugal: A comparison between coastal and inland habitats. Mammalia 60: 27–34. | | SCI Journals | | General ecology | |
| Beja P (1996) Temporal and spatial patterns of rest-site use by four female otters Lutra lutra along the south-west coast of Portugal. Journal of Zoology 239: 741–753. | | SCI Journals | | General ecology | |
| Beja P (1997) Predation by marine-feeding otters (Lutra lutra) in south-west Portugal in relation to fluctuating food resources. Journal of Zoology 242: 503–518. | | SCI Journals | | General ecology | |
| Beja P, Espírito-Santo C, Pedroso NM (2005) Estudos complementares do Plano de Ordenamento do Tejo Internacional. ERENA – Ordenamento e Gestão de Recursos Naturais, Lda/CARNIVORA – Núcleo de Estudos de Carnívoros e seus Ecossistemas. | | Technical report | | Population Status | |
| Beja P, Gordinho L, Reino L, Loureiro F, Santos-Reis M, Borralho R (2009) Predator abundance in relation to small game management in southern Portugal: conservation implications. European Journal of Wildlife Research 55: 227–238. | | SCI Journals | | Conservation | |
| Beja P, Schindler S, Santana J, Porto M, Morgado R, Moreira F, Pita R, Mira A, Reino L (2013) Predators and livestock reduce bird nest survival in intensive Mediterranean farmland. *European Journal of Wildlife Research* 60: 249–258. | | SCI Journals | | General ecology | |
| Bernardo J (2008) *Influência antropogénica na distribuição da lontra (Lutra lutra).* MSc thesis, University of Aveiro, Aveiro, Portugal. | | Thesis | | Population Status | |
| Bernardo J, Quaglietta L, Mira A, Fonseca C (2009) Uso del espacio en una población de nutrias en ambientes humanizados. In: *Resúmenes IX Jornadas de la SECEM*, Bilbao, Spain. | | Conference Proceedings | | General ecology | |
| Bernardo J, Roque S, Pedro AS, Petrucci-Fonseca F, Álvares F (2011) Implicaciones biológicas de la depredación del lobo (Canis lupus) al sur del Río Duero en Portugal: análisis exploratorio de las estadísticas oficiales de los ataques. In: *Resúmenes X Congreso de la SECEM*, Fuengirola, Málaga, Spain. | | Conference Proceedings | | Conservation | |
| Bessa-Gomes C (1996) *Contribuição para o estudo da distribuição do lobo em Portugal: caracterização da estrutura espacial da população no primeiro quinquénio da década de 90 e análise da adequação do habitat.* Final Degree thesis, Faculty of Sciences of the University of Lisbon Lisbon, Portugal. | | Thesis | | Population Status | |
| Bessa-Gomes C, Fernandes M, Abreu P, Castro LR, Ceia H, Pinto B, Pires AE (2002) Le lynx pardelle (Lynx pardinus) au Portugal: diverses approches dans un scénario de pré-extinction. In: L’Étude et la Conservation des Carnivores, 130–135. Société Française pour l’Etude et la Protection des Mammifères, Paris, France. | | Book | | Population Status | |
| Bessa-Gomes C, Petrucci-Fonseca F (1997) Análise preliminar da potencialidade de habitat para utilização pelo lobo (Canis lupus L. 1758) em Portugal. In: *Resúmenes I Congreso Hispano-Luso. Situación y conservación de las poblaciones de lobo en la Península Ibérica*, 67. Soria, Spain. | | Conference Proceedings | | General ecology | |
| Bessa-Gomes C, Petrucci-Fonseca F (1997) Caracterização de padrões de ocorrência e da estrutura espacial da população de lobos: a utilização de modelos lineares generalizados e redes neuronais artificiais para analisar a distribuição do lobo (Canis lupus L. 1758) em Portugal. In: *Resúmenes I Congreso Hispano-Luso. Situación y conservación de las poblaciones de lobo en la Península Ibérica*, 66. Soria, Spain. | | Conference Proceedings | | General ecology | |
| Bessa-Gomes C, Petrucci-Fonseca F (1998) Computer-intensive techniques for assessing distribution patterns: the wolf distribution in Portugal example. In: Reig S (ed) *Abstracts of the Euro-American Mammal Congress*, 260. Santiago Compostela, Spain. | | Conference Proceedings | | General ecology | |
| Bessa-Gomes C, Petrucci-Fonseca F (2003) Using artificial neural networks to assess wolf distribution patterns in Portugal. *Animal Conservation* 6: 221–229. | | SCI Journals | | Population Status | |
| Blanco-Garrido F, Prenda J, Narvaez M (2008) Eurasian otter (Lutra lutra) diet and prey selection in Mediterranean streams invaded by centrarchid fishes. *Biological Invasions* 10: 641–648. | | SCI Journals | | General ecology | |
| Bocage JVB (1863) Liste des mammifères et reptiles observées au Portugal. *Revue et Magazin de Zoologie* 15: 329–333. | | non-SCI Journals | | Population Status | |
| Bonesi L, Palazón S (2007) The American mink in Europe: Status, impacts, and control. *Biological Conservation* 134: 470–483. | | SCI Journals | | Population Status | |
| Bordelo JA, Martins R, Lempek MR, Viana TA (2012) Situação atual do lobo ibérico na região de Vila Real - Revisão bibliográfica. In: *Abstracts of the IV Congresso da Fauna Selvagem WAVES Portugal*, 61. Bragança, Portugal. | | Conference Proceedings | | Others | |
| Borges C, Marado C, Simões F, Mendonça D, Costa G, Almeida M et al. (2012) Non-invasive monitoring of canids at North of Douro River in Portugal. In: *Abstracts of the III Iberian Wolf Congress*, 17. Lugo, Spain. | | Conference Proceedings | | Genetics | |
| Borges C, Simões F, Mendonça D, Cadete D, Pinto S, Pires AE, Amorim do Rosário I, Matos J, Petrucci-Fonseca F (2012) Species assignment in feral attacks: a forensic issue. In: *Abstracts of the III Iberian Wolf Congress*, 70. Lugo, Spain. | | Conference Proceedings | | Conservation | |
| Borralho R, Rego F, Palomares F, Hora A (1996) The distribution of the Egyptian mongoose Herpestes ichneumon (L.) in Portugal. *Mammal Review* 26: 1–8. | | SCI Journals | | Population Status | |
| Cabaço N (2009) *Restos faunísticos em contexto funerário nos Perdigões, Reguengos de Monsaraz (Sepulcros 1 e 2).* MSc thesis, Universidade de Trás-os-Montes e Alto Douro, Vila Real, Portugal | | Thesis | | Others | |
| Cabral MJ, Almeida J, Almeida PR, Dellinger T, Ferrand de Almeida N, Oliveira ME et al. (2005) Livro Vermelho dos Vertebrados de Portugal. Instituto da Conservação da Natureza, Lisbon. | | Book | | Conservation | |
| Cadete D, Pinto S, Borges C, Simões F, Petrucci-Fonseca F (2012) O lobo na região fronteiriça portuguesa a sul do rio Douro: desafios à monitorização e conservação. In: *Abstracts of the III Iberian Wolf Congress*, 36. Lugo, Spain. | | Conference Proceedings | | Conservation | |
| Campos MI (1993) *Análise da situação actual da Lutra lutra (L. 1758) na Reserva do Estuário do Sado.* Faculty of Sciences of the University of Lisbon, Lisbon, Portugal. | | Thesis | | Population Status | |
| Campos MT (1997) Cuantificación y caracterización de los lugares de reposo de la nutria (Lutra lutra) en el Parque Nacional de Peneda-Gerês. In: *Resúmenes III Jornadas de la SECEM*, Castelló d’Empúries, Gerona, Spain. | | Conference Proceedings | | General ecology | |
| Cândido AT, Petrucci-Fonseca F (2000) O lobo da Serra da Estrela: passado, presente e futuro. *Galemys* 12: 209–222. | | non-SCI Journals | | Population Status | |
| Caninas JC, Henriques F, Álvares F (2014) Apiary-walls and pitfall-traps in Portugal: Archaic constructions for wild animals. In: Correia M, Carlos G, Rocha S (eds) *Vernacular Heritage and Earthen Architecture: Contributions for Sustainable Development*, 465–470. CRC Press / Taylor & Francis Group, London. | | Book | | Conservation | |
| Cardoso JL (1996) The large Upper-Pleistocene mammals in Portugal. A synthetical approach. *Geobios* 29: 235–250. | | SCI Journals | | Others | |
| Cardoso JL, Morais IA, Guérin C (1993) *Contribuição para o conhecimento dos grandes mamíferos do Plistocénico Superior de Portugal.* Câmara Municipal de Oeiras, Oeiras, Portugal. | | Book | | Others | |
| Cardoso L, Cortes HCE, Eyal O, Reis A, Lopes AP, Vila-Viçosa MJ, Rodrigues PA, Baneth G (2014) Molecular and histopathological detection of Hepatozoon canis in red foxes (Vulpes vulpes) from Portugal. *Parasites & Vectors* 7: 113. | | SCI Journals | | Health status | |
| Cardoso L, Cortes HCE, Reis A, Rodrigues PA, Simões M, Lopes AP et al. (2013) Prevalence of Babesia microti-like infection in red foxes (Vulpes vulpes) from Portugal. *Veterinary Parasitology* 196: 90–5. | | SCI Journals | | Health status | |
| Cardoso L, Gilad M, Cortes H, Nachum-Biala Y, Lopes A, Vila-Viçosa M, Simões M, Rodrigues PA, Baneth G (2015) First report of Anaplasma platys infection in red foxes (Vulpes vulpes) and molecular detection of Ehrlichia canis and Leishmania infantum in foxes from Portugal. *Parasites & Vectors* 8: 144. | | SCI Journals | | Health status | |
| Carmo P (1998) Factors affecting wolf population in Portugal and the possible role of a protected areas network on this species conservation. In: Reig S (ed) *Abstracts of the Euro-American Mammal Congress*, 257. Santiago Compostela, Spain. | | Conference Proceedings | | Conservation | |
| Carmo P, Barroso I (1997) Conservaçâo do lobo (Canis lupus) em Portugal: estado actual dos conhecimentos e perspectivas. In: *Resúmenes I Congreso Hispano-Luso. Situación y conservación de las poblaciones de lobo en la Península Ibérica, Soria, Spain.* | | Conference Proceedings | | Conservation | |
| Carmo P, Barroso I (1997) Gestão do lobo (Canis lupus) em Portugal. In: *Resúmenes I Congreso Hispano-Luso. Situación y conservación de las poblaciones de lobo en la Península Ibérica, Soria, Spain.* | | Conference Proceedings | | Conservation | |
| Carreira M (2010) *Contribuição para o Estudo da Ecologia do Lobo Ibérico no Distrito de Vila Real.* MSc thesis, Faculty of Sciences of the University of Lisboa, Lisbon, Portugal. | | Thesis | | General ecology | |
| Carreira R (1996) *O lobo no Parque Natural do Alvão: distribuição e ecologia.* Final Degree thesis, Faculty of Sciences of the University of Lisbon Lisbon, Portugal. | | Thesis | | Population Status | |
| Carreira R (1996) *Situação populacional e biologia alimentar do lobo na área de influência do Parque Natural do Alvão.* Final Degree thesis, Faculty of Sciences of the University of Lisbon Lisbon, Portugal. | | Thesis | | Population Status | |
| Carreira R, Petrucci-Fonseca F (2000) O lobo na região oeste de Trás-os-Montes (Portugal). *Galemys* 12: 123–134. | | non-SCI Journals | | Population Status | |
| Carvalho F (2004) *Modelação da Abundância de Carnívoros na Serra Monfurado.* Final Degree thesis, University of Évora, Évora, Portugal. | | Thesis | | Population Status | |
| Carvalho F, Carvalho R, Galantinho A, Mira A, Beja P (2015) Monitoring frequency influences the analysis of resting behaviour in a forest carnivore. *Ecological Research* 30: 537–546. | | SCI Journals | | General Ecology | |
| Carvalho F, Carvalho R, Mira A, Beja P (2014) Use of tree hollows by a Mediterranean forest carnivore. Forest Ecology and Management 315: 54–62. | | SCI Journals | | General ecology | |
| Carvalho F, Galantinho A, Mira A (2011) Factors affecting small carnivore occurrence and abundance in Mediterranean agricultural landscapes. Case studies in southern Portugal. In: Rosalino LM, Gheler-Costa C (eds) *Middle-Sized Carnivores in Agricultural Landscapes*, 39–68. Nova Science Publishers, Inc., New York, U.S.A. | | Book | | General ecology | |
| Carvalho F, Mira A (2010) Comparing annual vertebrate road kills over two time periods, 9 years apart: a case study in Mediterranean farmland. *European Journal of Wildlife Research* 57: 157–174. | | SCI Journals | | Conservation | |
| Carvalho JC, Gomes P (2001) Food habits and trophic niche overlap of the red fox, European wild cat and common genet in the Peneda-Gerês National Park. *Galemys* 13: 39–48. | | non-SCI Journals | | General ecology | |
| Carvalho JC, Gomes P (2004) Feeding resource partitioning among four sympatric carnivores in the Peneda-Gerês National Park (Portugal). *Journal of Zoology* 263: 275–283. | | SCI Journals | | General ecology | |
| Carvalho R (2012) *Fatores determinantes da intensidade de uso dos abrigos pela geneta (Genetta genetta L. 1758) numa região mediterrânica. MSc* thesis, University of Évora, Évora, Portugal. | | Thesis | | General Ecology | |
| Carvalho S (2001) *Avaliação do habitat para o texugo (Meles meles, L.) na Serra de Grândola: Uma abordagem utilizando um SIG.* Final Degree thesis, Faculty of Sciences of the University of Lisbon Lisbon, Portugal. | | Thesis | | General ecology | |
| Carvalhovalera M (1985) The Red Fox (Vulpes vulpes), disclosed as a source of helmintho-zoonoses in Portugal. *Revue d’Ecologie, La Terre et la Vie* 40: 239–239. | | SCI Journals | | Health status | |
| Castro D, Monterroso P, Moreira P, Alves PC (2007) Does Scat Morphological Misidentification Influence Ecological Studies? A Case Study with the European Wildcat (Felis silvestris) in Portugal. In: Hughes J, Mercer R (eds) *Abstracts of the Felid Biology and Conservation Conference*, 77. Oxford University, Oxford, U.K. | | Conference Proceedings | | Others | |
| Castro LR (1994) Ecologia y conservación del lince ibérico en la sierra portuguesa de Malcata. Quercus 2: 8–12. | | non-SCI Journals | | Conservation | |
| Castro LR (1995) Aplicación de un modelo de Indice de Adecuación de Hábitat (HSI) a las poblaciones de lince ibérico de la Sierra de Malcata (centro y este de Portugal). In: *Resúmenes II Jornadas de la SECEM*, Soria, Spain. | | Conference Proceedings | | Conservation | |
| Castro LR, Fernandes M, Sarmento P, Abreu P, Ceia H (1998) Preliminary results of some studies on iberian lynx in Portugal. In: Reig S (ed) Abstracts of the Euro-American Mammal Congress, Santiago Compostela, Spain. | | Conference Proceedings | | Population Status | |
| Castro LR, Palma L (1996) The current status, study and conservation of Iberian Lynx in Portugal. *Journal of Wildlife Research* 2: 179–181. | | non-SCI Journals | | Population Status | |
| Ceia H, Abreu P, Castro LR, Fernandes M, Sarmento P (1998) Conservation of iberian lynx in Portugal. In: Reig S (ed) Abstracts of the Euro-American Mammal Congress, Santiago Compostela, Spain. | | Conference Proceedings | | Conservation | |
| Ceia H, Castro LR, Fernandes M, Abreu P (1998) *Lince-ibérico em Portugal. Bases para a sua conservação. Relatório final do Projecto “Conservação do lince-ibérico”.* Instituto de Conservação da Natureza, Lisbon, Portugal. | | Technical report | | Conservation | |
| Ceia H, Castro LR, Fernandes M, Abreu P (1998) Past and present situation of the iberian lynx in Portugal. In: Reig S (ed) Abstracts of the Euro-American Mammal Congress, Santiago Compostela, Spain. | | Conference Proceedings | | Population Status | |
| Cerqueira L (2005) *Distribuição e ecologia alimentar da Lontra (Lutra lutra) em dois sistemas costeiros em Portugal.* MSc thesis, University of Minho, Braga, Portugal. | | Thesis | | General ecology | |
| Chambel I (1997) *Aspectos da ecologia da lontra (Lutra lutra) na área do Parque Natural do Alvão.* Final Degree thesis, Faculty of Sciences of the University of Lisbon Lisbon, Portugal. | | Thesis | | General ecology | |
| Chambel I (1997) *Situação actual da lontra (Lutra lutra L., 1758) na Reserva Natural do Sapal de Castro Marim e Vila Real de Santo António*. Instituto da Conservação da Natureza - Programa LIFE, Lisbon, Portugal. | | Technical report | | Population Status | |
| Chambel I, Mota M, Rodrigues M, Santos-Reis M (2001) *Inventariação de Mamíferos Não Voadores na Área da Paisagem Protegida da Arriba Fóssil da Costa de Caparica.* | | Technical report | | Population Status | |
| Chambel I, Trindade A, Santos-Reis M (1998) Use of trophic resourses by the eurasian-otter in the Alvão Natural Park (North of Portugal). In: Reig S (ed) *Abstracts of the Euro-American Mammal Congress*, Santiago Compostela, Spain. | | Conference Proceedings | | General ecology | |
| Clamote FT, Santos-Reis M (1997) Primera aproximación al estudio de la dieta del meloncillo (Herpestes ichneumon L.) en Portugal. *Resúmenes III Jornadas de la SECEM*, Castelló d’Empúries, Gerona, Spain. | | Conference Proceedings | | General ecology | |
| Coimbra E (2012) Gestão de sistemas socio-ambientais: seguindo os lobos na senda da sustentabilidade. In: *Abstracts of the III Iberian Wolf Congress*, 80. Lugo, Spain. | | Conference Proceedings | | Conservation | |
| Conceição-Neto N, Zeller M, Heylen E, Lefrère H, Mesquita JR, Matthijnssens J (2015) Fecal virome analysis of three carnivores reveals a novel nodavirus and multiple gemycircularviruses. *Virology Journal* 12: 79. | | SCI Journals | | Health status | |
| Conceição-Silva FM, Abranches P, Silva-Pereira MC, Janz JG (1988) Hepatozoonosis in foxes from Portugal. *Journal of Wildlife Diseases* 24: 344–347. | | SCI Journals | | Health status | |
| Correia AM (2001) Seasonal and interspecific evaluation of predation by mammals and birds on the introduced red swamp crayfish Procambarus clarkii (Crustacea, Cambaridae) in a freshwater marsh (Portugal). *Journal of Zoology* 255: 533–541. | | SCI Journals | | General ecology | |
| Costa B (2001) *Alguns aspectos do comportamento do Lobo Ibérico (Canis lupus signatus Cabrera, 1907) em cativeiro: influência de factores de perturbação.* Final Degree thesis, Faculty of Sciences of the University of Lisbon Lisbon, Portugal. | | Thesis | | General ecology | |
| Costa H, Santos-Reis M (2002) Use of middens by the common genet (Genetta genetta L.) and its relation with the landscape structure in Grândola Mountain (SW Portugal). *Revista de Biologia*: 135–145. | | non-SCI Journals | | General ecology | |
| Costa M, Fernandes C, Bruford MW, Santos-Reis M (2007) Mitochondrial DNA phylogeography of the European polecat (Mustela putorius) in Portugal. In: Hajkova P, Rüziczová O (eds) *Abstracts of the 25th Mustelid Colloquium*, 56. Trebon, Czech Republic. | | Conference Proceedings | | Genetics | |
| Costa M, Fernandes C, Rodrigues M, Santos-Reis M (2012) A panel of microsatellite markers for genetic studies of European polecats (Mustela putorius) and ferrets (Mustela furo). *European Journal of Wildlife Research* 58: 629–633. | | SCI Journals | | Genetics | |
| Costa M, Fernandes C, Santos-Reis M (2014) Ecology and conservation of the polecat Mustela putorius (Linnaeus , 1758) in Portugal: a review. In: Ruiz-González A, Rubines J, Luzuriaga JL (eds) *Conservation and Management of semi-aquatic mammals of Southwestern Europe*, 79–87. Aranzadi Society of Sciences. | | Book | | Conservation | |
| Costa M, Ferrão da Costa G, Guerra A, Petrucci-Fonseca F (2009) Uso del maxent para predecir la idoneidad del hábitat para el lobo en una región del norte de Portugal. In: *Resúmenes IX Jornadas de la SECEM*, 54. Bilbao, Spain. | | Conference Proceedings | | Population Status | |
| Cruz J (1996) *Estudo da utilização espácio-temporal da geneta (Genetta genetta L. 1758) e caracterização de uma comundade de micromamíferos na Reserva Natural da Serra da Malcata.* Final Degree thesis, Faculty of Sciences of the University of Porto, Porto, Portugal. | | Thesis | | General ecology | |
| Cruz J, Capinha M, Paula A, Pereira F, Monteiro B, Salazar D, Paula J (2007) *Consultoria técnica para avaliação da recuperação do ecossistema do lince-ibérico no âmbito do Projecto POA “Gestão de Espécies e Habitats na RNSM” - 2004-2006*. Terra e Tudo - Consultoria e Gestão de Recursos Naturais, Unipessoal Lda. Aranhas, Portugal. | | Technical report | | Conservation | |
| Cruz J, Sarmento P, Vingada J V., Eira C, Fonseca C, Soares M, Soares A (1998) Feeding ecology of the european genet in a mountain and in a coastal habitat. In: Reig S (ed) *Abstracts of the Euro-American Mammal Congress*, Santiago Compostela, Spain. | | Conference Proceedings | | General ecology | |
| Cruz J, Sarmento P, Vingada J V., Eira C, Fonseca C, Soares M, Soares A (1998) Some ecological aspects of the red fox at Serra da Malcata. In: Reig S (ed) *Abstracts of the Euro-American Mammal Congress*, Santiago Compostela, Spain. | | Conference Proceedings | | General ecology | |
| Cruz J, Sarmento P, Vingada J V., Soares A (1998) Spatiotemporal ecology of the european genet in centre Portugal. In: Reig S (ed) *Abstracts of the Euro-American Mammal Congress*, Santiago Compostela, Spain. | | Conference Proceedings | | General ecology | |
| Cruz J, Sarmento P, White PCL (2015) Influence of exotic forest plantations on occupancy and co-occurrence patterns in a Mediterranean carnivore guild. *Journal of Mammalogy* 96: 854–865. | | SCI Journals | | General Ecology | |
| Cruz M (2000) *Estudo da actividade e movimentos do texugo utilizando a rádio-triangulação.* Final Degree thesis, Faculty of Sciences of the University of Lisbon Lisbon, Portugal. | | Thesis | | General ecology | |
| Cruz T, Fonseca C, Carvalho J, Oliveira B, Torres RT (2014) Roe deer reintroduction in central Portugal: a tool for Iberian wolf conservation. *Galemys* 26: 31–40. | | non-SCI Journals | | Conservation | |
| Cumbassá A (2013) *Sources and routes of transmission of Q fever: detection, identification and molecular typing of Coxiella burnetti in domestic and wild animals.* MSc thesis, Faculty of Sciences of the University of Lisbon, Lisbon, Portugal. | | Thesis | | Health status | |
| Cumbassá A, Barahona MJ, Cunha M V, Azórin B, Fonseca C, Rosalino LM et al. (2015) Coxiella burnetii DNA detected in domestic ruminants and wildlife from Portugal. *Veterinary microbiology* 180: 136–41. | | SCI Journals | | Health status | |
| Curado N, Loureiro F, Lourenço P, Santos E (2011) Selección a nivel regional de áreas prioritarias donde llevar a cabo acciones de conservación para el lince ibérico en el SE de Portugal. In: *Resúmenes X Congreso de la SECEM*, Fuengirola, Málaga, Spain. | | Conference Proceedings | | Conservation | |
| Davis SJM (2002) The mammals and birds from the Gruta do Caldeirão, Portugal. *Natural History* 5: 29–98. | | SCI Journals | | Others | |
| Delibes-Mateos M, Blázquez MC, Blanco-Garrido F, Sánchez J, Segura A, Delibes M (2014) Sprainting sites and feeding habits of the otter (Lutra lutra) in the Douro River estuary, Portugal. *Galemys* 26: 91–95. | | non-SCI Journals | | General ecology | |
| Delibes-Mateos M, Delibes M, Ferreras P, Villafuerte R (2008) Key role of European rabbits in the conservation of the Western Mediterranean basin hotspot. *Conservation Biology* 22: 1106–1117. | | SCI Journals | | Conservation | |
| Detry C, Bicho N, Fernandes H, Fernandes C (2011) The Emirate of Córdoba (756-929 AD) and the introduction of the Egyptian mongoose (Herpestes ichneumon) in Iberia: the remains from Muge, Portugal. *Journal of Archaeological Science* 38: 3518–3523. | | SCI Journals | | Population Status | |
| Detry C, Cardoso JL (2010) On some remains of dog (Canis familiaris) from the Mesolithic shell-middens of Muge, Portugal. *Journal of Archaeological Science* 37: 2762–2774. | | SCI Journals | | Others | |
| Dias D, Petrucci-Fonseca F, Ramos MJ, Santos-Reis M, Oom M do M (1983) Os vertebrados do Algarve e o seu enquadramento num projecto de ordenamento do território. *Boletim da LPN* 17: 21–45. | | non-SCI Journals | | Population Status | |
| Díaz Álvarez E (2013) *Prejuízos e conflituosidade social do urso-pardo e do lobo na Península Ibérica.* MSc thesis, Faculty of Sciences of the University of Lisbon, Lisbon, Portugal. | | Thesis | | Conservation | |
| Díaz-Ruiz F, Delibes-Mateos M, García-Moreno JL, María López-Martín J, Ferreira C, Ferreras P (2013) Biogeographical patterns in the diet of an opportunistic predator: the red fox Vulpes vulpes in the Iberian Peninsula. *Mammal Review* 43: 59–70. | | SCI Journals | | General ecology | |
| Domingos SA, Santos-Reis M (1997) Una primera aproximación al uso del tiempo y del espacio por el meloncillo en Portugal. In: *Resúmenes II Jornadas de la SECEM*, Castelló d’Empúries, Gerona, Spain. | | Conference Proceedings | | General ecology | |
| Domingues J (2005) Caça ao Lobo: Legislação Arcaica. *Agália, Revista de Ciências Sociais e Humanidades* 83/84: 265–269. | | non-SCI Journals | | Conservation | |
| Duarte A (2012) *Abundância relativa do visão-americano (Neovison vison): análise comparativa da eficácia de duas técnicas.* MSc thesis, Faculty of Sciences of the University of Lisboa, Lisbon, Portugal. | | Thesis | | Population Status | |
| Duarte A, Fernandes M, Santos N, Tavares L (2012) Virological Survey in free-ranging wildcats (Felis silvestris) and feral domestic cats in Portugal. *Veterinary Microbiology* 158: 400–404. | | SCI Journals | | Health status | |
| Duarte MD, Henriques AM, Barros SC, Fagulha T, Mendonça P, Carvalho P et al. (2013) Snapshot of Viral Infections in Wild Carnivores Reveals Ubiquity of Parvovirus and Susceptibility of Egyptian Mongoose to Feline Panleukopenia Virus. *PLoS ONE* 8. | | SCI Journals | | Health status | |
| Eggermann J, Da Costa GF, Guerra AM, Kirchner WH, Petrucci-Fonseca F (2011) Presence of Iberian wolf (Canis lupus signatus) in relation to land cover, livestock and human influence in Portugal. *Mammalian Biology* 76: 217–221. | | SCI Journals | | General ecology | |
| Eira C, Soares M, Fonseca C, Faria M, Ferreira S, Ferreira AJ et al. (1998) Impact of red fox (Vulpes vulpes) in small game species. Is this true or just another tale? In: Reig S (ed) *Abstracts of the Euro-American Mammal Congress*, Santiago Compostela, Spain. | | Conference Proceedings | | Conservation | |
| Eira C, Vingada J, Torres J, Miquel J (2006) The Helminth Community of the Red Fox, Vulpes Vulpes, In Dunas de Mira (Portugal) and its effect on host condition. *Wildlife Biology in Practice* 2: 26–36. | | non-SCI Journals | | Health status | |
| Encarnação C, Mira A (2005) Modelación de la abundancia de la nutria (Lutra lutra Linnaeus, 1758) en el “Sítio Monfurado” (Portugal) en relación a la naturalización de las líneas de agua. In: *Resúmenes VII Jornadas de la SECEM*, Valencia, Spain. | | Conference Proceedings | | General ecology | |
| Equipa Corço (1992) *Relatório de Actividade 1991-92.* | | Technical report | | Population Status | |
| Espírito-Santo C (1999) *O comportamento de marcação de Genetta genetta Linnaeus, 1758 na Serra de Grândola.* Faculty of Sciences of the University of Lisbon, Lisbon, Portugal. | | Thesis | | General ecology | |
| Espirito-Santo C (2007) *Human dimensions in Iberian wolf management in Portugal: attitudes and beliefs of interest groups and the public toward a fragmented wolf population.* MSc thesis, Memorial University of Newfoundland, Canada | | Thesis | | Conservation | |
| Espírito-Santo C, Bath A, Petrucci-Fonseca F (2000) Public Attitudes Toward the Iberian Wolf: a Portuguese Study. In: *Proceedings and Agenda for Defenders of Wildlife’s Carnivores 2000: A Conference on Carnivore Conservation in the 21st Century*, Denver, Colorado, U.S.A. | | Conference Proceedings | | Conservation | |
| Espírito-Santo C, Freitas S (2012) O papel do ecoturismo na conservação do lobo-ibérico em Portugal. In: *Abstracts of the III Iberian Wolf Congress*, 75. Lugo, Spain. | | Conference Proceedings | | Conservation | |
| Espírito-Santo C, Petrucci-Fonseca F (2004) Human dimension in Iberian wolf management in Portugal. *Wolf Print* 20: 7–9. | | non-SCI Journals | | Conservation | |
| Espírito-Santo C, Petrucci-Fonseca F (2007) Perceptions of the Agricultural World and of Environmental Governmental Authorities about the Compensation System in Portugal. In: *Proceedings of the International Symposium: Large Carnivores and Agriculture Comparing Experiences across Italy and Europe*, 29. Assisi, Italy. | | Conference Proceedings | | Conservation | |
| Espírito-Santo C, Rosalino LM, Santos-Reis M (2007) Factors affecting the placement of common genet latrine sites in a Mediterranean landscape in Portugal. *Journal of Mammalogy* 88: 201–207. | | SCI Journals | | General ecology | |
| Espírito-Santo C, Santos-Reis M (1999) El papel de los excrementos como instrumento de marcaje por la Gineta en el SO de Portugal. In: *Resúmenes IV Jornadas de la SECEM*, Segovia, Spain. | | Conference Proceedings | | General ecology | |
| Farinha N (1995) *Distribuição e ecologia da lontra (Lutra lutra Linnaeus, 1758) no Parque Natural de Montesinho*. Instituto da Conservação da Natureza, Lisbon, Portugal. | | Technical report | | Population Status | |
| Farinha N, Florêncio E, Trindade A (1997) Distribução da Lontra (Lutra lutra) em Portugal Continental. In: *Resúmenes III Jornadas de la SECEM*, Castelló d’Empúries, Gerona, Spain. | | Conference Proceedings | | Population Status | |
| Farinha N, Trindade A (1997) A Lontra (Lutra lutra) na Reserva Natural do Estuário do Tejo. In: *Resúmenes III Jornadas de la SECEM*, Castelló d’Empúries, Gerona, Spain. | | Conference Proceedings | | Population Status | |
| Fernandes CA, Ginja C, Pereira Í, Tenreiro R, Bruford MW, Santos-Reis M (2008) Species-specific mitochondrial DNA markers for identification of non-invasive samples from sympatric carnivores in the Iberian Peninsula. *Conservation Genetics* 9: 681–690. | | SCI Journals | | Genetics | |
| Fernandes CA, Rodrigues M, Hajji, Ghaiet M, Vercammen P, Bruford MW, Santos-Reis M (2009) Isolation and characterisation of 11 tetranucleotide microsatellite loci in the common genet (Genetta genetta). *Conservation Genetics* 10: 1931–1934. | | SCI Journals | | Genetics | |
| Fernandes M (2007) Ocorrência de gato-bravo em Portugal. Unidade de Espécies e Habitats/Instituto da Conservaçã da Natureza e das Florestas. | | Technical report | | Population Status | |
| Fernandes M (2011) Plano de análise e vigilância sanitária nas áreas potenciais de reintrodução do Lince-ibérico. In: *Abstracts of the III Congresso da Fauna Selvagem*, Vila Real, Portugal. | | Conference Proceedings | | Conservation | |
| Fernandes ML (1991) *Alguns aspectos da ecologia e sistemática do gato-bravo (*Felis silvestris *Schreber, 1777).* Final Degree thesis, Faculty of Sciences of the University of Lisbon Lisbon, Portugal. | | Thesis | | General Ecology | |
| Fernandes ML (1996) *Estudo genético do gato-bravo europeu: abordagem ao problema da hibridação.* Faculty of Sciences of the University of Lisbon, Lisbon, Portugal. | | Thesis | | Genetics | |
| Fernández González A, García Hermosell I, Munné Prat S, Álvares F, Fernández Tuya P, Fernández Menéndez D (2012) Uso combinado de diferentes técnicas de monitorización de lobo ibérico en áreas de baja densidad: seguimiento de grupos reproductores en la cuenca media - alta del Tâmega (Norte de Portugal). In: *Abstracts of the III Iberian Wolf Congress*, 35. Lugo, Spain. | | Conference Proceedings | | Population Status | |
| Fernández González A, Munné Prat S, García Hermosell I, Álvares F, Fernández Tuya P, Fernández Menéndez D (2012) Aplicación de técnicas de trampeo fotográfico y confirmación genética para determinar la abundancia relativa de perro en áreas de escasa densidad de lobo ibérico. In: *Abstracts of the III Iberian Wolf Congress*, 42. Lugo, Spain. | | Conference Proceedings | | Conservation | |
| Ferrand de Almeida F (1986) Alguns aspectos da biologia e da distribuição da lontra em Portugal. *Quercus* 5: 14. | | non-SCI Journals | | Population Status | |
| Ferrand de Almeida F (1986) Principais ameaças à lontra em Portugal. *Quercus* 5: 14. | | non-SCI Journals | | Conservation | |
| Ferrand de Almeida F (1987) À propos de la distribution de la loutre au Portugal. *Ciência Biológica. Ecology and Systematics* 7: 11–15. | | non-SCI Journals | | Population Status | |
| Ferrão da Costa G (2000) *Situação populacional e ecologia trófica do lobo-ibérico (Canis lupus signatus Cabrera, 1907) na Serra do Soajo.* Final Degree thesis, Faculty of Sciences of the University of Lisbon Lisbon, Portugal. | | Thesis | | Population Status | |
| Ferrão da Costa G, Almeida M, Borges C, Simões F, Petrucci-Fonseca F (2012) Scats surveys versus camera trapping: comparison of noninvasive methods for impacts assessment studies in wolf low-density areas. In: *Abstracts of the III Iberian Wolf Congress*, 14. Lugo, Spain. | | Conference Proceedings | | Others | |
| Ferrão da Costa G, Guerra A, Eggermann J, Petrucci-Fonseca F (2006) Planning new highways on large carnivore territories – the wolf case in Portugal. In: *Abstracts of the 1st European Congress on Conservation Biology*, 109. Eger, Hungary. | | Conference Proceedings | | Conservation | |
| Ferreira AM (1991) *Alguns aspectos da ecologia da raposa (Vulpes vulpes silacea Miller, 1907) no Parque Natural de Montesinho.* Final Degree thesis, Faculty of Sciences of the University of Lisbon Lisbon, Portugal. | | Thesis | | General ecology | |
| Ferreira C (2011) *A situação do coelho-bravo na Península Ibérica: implicações para a conservação do Lince-ibérico.* | | Technical report | | Conservation | |
| Ferreira C, Alves PC (2005) *Impacto da implementação de medidas de gestão do habitat nas populações de coelho-bravo (Oryctolagus cuniculus algirus) no Parque Natural do Sudoeste Alentejano e Costa Vicentina*. Instituto da Conservação da Natureza/Research Center in Biodiversity and Genetic Resources - University of Porto. | | Technical report | | Conservation | |
| Ferreira C, Rio-Maior H, Roque S, Petrucci-Fonseca F, Brandão R, Santos N, Álvares F (2012) Hematological and serum biochemical reference values of free-ranging Iberian wolves in Portugal. In: *Abstracts of the III Iberian Wolf Congress*, 25. Lugo, Spain. | | Conference Proceedings | | Health status | |
| Ferreira I, Silva M, Santos N, Rio-Maior H, Nakamura M, Álvares F, Carvalho LMM de (2012) Rastreio sorológico de alguns agentes de zoonoses em canídeos silvestres no Norte de Portugal. *Abstracts of the III Iberian Wolf Congress*, 57. Lugo, Spain. | | Conference Proceedings | | Health status | |
| Ferreira JP (2003) *Análise dos factores condicionantes na distribuição de gato-bravo (Felis silvestris) no Sítio Moura-Barrancos.* MSc thesis, University of Évora, Évora, Portugal. | | Thesis | | Population Status | |
| Ferreira JP (2010) *Integrating anthropic factors into wildcat Felis silvestris conservation in Southern Iberia landscapes.* PhD thesis, Faculty of Sciences of the University of Lisbon, Lisbon, Portugal. | | Thesis | | Conservation | |
| Ferreira JP, Álvares F (2005) Gato-bravo, outro felídeo ameaçado em Portugal. *Tribuna da Natureza* 21: 4–10. | | non-SCI Journals | | Conservation | |
| Ferreira JP, Rosalino LM, Santos-Reis M (1999) *Avaliação qualitativa e quantitativa do património natural da Herdade da Coitadinha: componente mamíferos não voadores*. Centre for Environmental Biology/Empresa de Desenvolvimento e Infraestruturas de Alqueva, S.A, Lisbon, Portugal. | | Technical report | | Population Status | |
| Ferreira JP, Santos-Reis M (1997) La gineta en el Perímetro Forestal da Contenda (SE Portugal). In: *Resúmenes III Jornadas de la SECEM*, Castelló d’Empúries, Gerona, Spain. | | Conference Proceedings | | Population Status | |
| Ferreira JP, Santos-Reis M (1998) Managing mediterranean habitats: the common genet as a keystone species. In: rei (ed) *Abstracts of the Euro-American Mammal Congress*, Santiago Compostela, Spain. | | Conference Proceedings | | Conservation | |
| Figueiredo D (2005) *Monitorização do Património Natural da Albufeira de Pedrógão.* | | Technical report | | Population Status | |
| Figueroa TV, Delibes M (1987) Primeros datos sobre el visón americano (Mustela vison) en el suroeste de Galicia y noroeste de Portugal. *Ecología* 1: 145–152. | | non-SCI Journals | | Population Status | |
| Fino F, Torres J, Petrucci-Fonseca F, Segovia J-M, Miquel J, Bacellar F, Feliu C (1995) Helmintofauna de Canis lupus signatus Cabrera, 1907 (Carnivora: Canidae) en Portugal mediante técnicas coprológicas. In: *Resúmenes IV Congreso Iberico de Parasitologia*, 41–42. Sociedade Portuguesa de Parasitologia & Asociacion de Parasitologos Españoles, Santiago de Compostela, Espanha. | | Conference Proceedings | | Health status | |
| Florêncio E (1993) *O lagostim do rio (Procambarus clarkii Girard, 1852) como recurso alimentar da comunidade de carnívoros na Reserva Natural do Paúl do Boquilobo.* Faculty of Sciences of the University of Lisbon, Lisbon, Portugal. | | Thesis | | General ecology | |
| Florêncio E (1994) *Distribuição e ecologia trófica da lontra (Lutra lutra L.) na bacia hidrográfica do rio Almonda*. Instituto da Conservação da Natureza, Lisbon, Portugal. | | Technical report | | Population Status | |
| Florêncio E (1995) *Distribuição da lontra (Lutra lutra Linnaeus, 1758) em Portugal Continental: Região a Norte do Rio Tejo*. Instituto da Conservação da Natureza, Lisbon, Portugal. | | Technical report | | Population Status | |
| Flower E (1971) Lobos em Portugal (1933-1957). Direcção Geral dos Serviços Florestais e Aquícolas, Lisbon | | Book | | Population Status | |
| Fonseca AM (2013) *Comportamento do lobo ibérico em cativeiro: impacto dos visitantes e educação ambiental.* MSc thesis, Faculty of Sciences of the University of Lisbon, Lisbon, Portugal. | | Thesis | | Conservation | |
| Fonseca de Ambrósio IS, Marcos AF, Órfão I, Petrucci-Fonseca F (2012) O Centro de Recuperação do Lobo Ibérico: sensibilizar e envolver a sociedade na conservação do lobo. In: *Abstracts of the III Iberian Wolf Congress*, 82. Lugo, Spain. | | Conference Proceedings | | Conservation | |
| Fonseca V, Quaglietta L (2011) Fine-scale spatial genetic structure and dispersal of the Eurasian Otter (Lutra lutra) in a Mediterranean Environment. In: Prigioni C, Loy A, Balestrieri A, Remonti L (eds) *Proceedings of the XIth International Otter Colloquium*, 101. Associazone Teriologica Italiana, Pavia, Italy. | | Conference Proceedings | | Genetics | |
| Fontes L, Campos A (1998) Os Fojos de Lobo da Cabreira. *Mínia* 6: 73–94. | | non-SCI Journals | | Conservation | |
| Fordham DA, Akçakaya HR, Brook BW, Rodríguez A, Alves PC, Civantos E, Triviño M, Watts MJ, Araújo MB (2013) Adapted conservation measures are required to save the Iberian lynx in a changing climate. *Nature Climate Change* 3: 899–903. | | SCI Journals | | Conservation | |
| Fragoso S (1999) *Distribuição e alguns aspectos da ecologia da doninha (Mustela nivalis Linnaeus, 1766) no Parque Natural das Serras de Aire e Candeeiros.* Final Degree thesis, Faculty of Sciences of the University of Lisbon Lisbon, Portugal. | | Thesis | | Population Status | |
| Fragoso S, Santos-Reis M (1999) Abundancia y uso del espacio por la Comadreja en el Parque Natural Serra de Aire e Candeeiros (Portugal). In: *Resúmenes IV Jornadas de la SECEM*, Segovia, Spain. | | Conference Proceedings | | General ecology | |
| Fragoso S, Santos-Reis M (2000) Utilização dos recursos tróficos pela doninha no Parque Natural das Serra de Aire e Candeeiros. *Revista de Biologia* 18: 23–32. | | SCI Journals | | General ecology | |
| Franco I (2000) *Aspectos Ecológicos da Mortalidade de Vertebrados em Rodovias do Interior Alentejano.* Faculty of Sciences of the University of Lisbon, Lisbon, Portugal. | | Thesis | | Conservation | |
| Freitas D (1999) *A dieta da lontra (Lutra lutra) ao longo da bacia hidrográfica do rio Tejo*. Final Degree thesis, Faculty of Sciences of the University of Lisbon Lisbon, Portugal. | | Thesis | | General ecology | |
| Freitas D, Gomes J, Sales-Luís T, Madruga L, Marques C, Baptista G et al. (2007) Otters and fish farms in the Sado estuary: ecological and socio-economic basis of a conflict. *Hydrobiologia* 587: 51–62. | | SCI Journals | | Conservation | |
| Freitas D, Sales-Luís T, Santos-Reis M (2003) Evaluación del conflicto entre el hombre y la nutria (Lutra lutra) en las piscifactorias de Portugal. In: *Resúmenes VI Jornadas de la SECEM*, Ciudad Real, Spain. | | Conference Proceedings | | Conservation | |
| Freitas D, Santos-Reis M (2005) Ecuaciones de regresión de Sparus aurata, Dicentrarchus labrax y Solea senegalensis en una región del estuario del Sado (Portugal): aplicación al estudio de la dieta de la nutria (Lutra lutra). In: *Resúmenes VII Jornadas de la SECEM*, Valencia, Spain. | | Conference Proceedings | | General ecology | |
| Freitas MC (2014) *Assessing the spatial extent of wolf-dog hybridization in real-time and at population level using non-invasive DNA sampling.* MSc thesis, Faculty of Sciences of the University of Porto, Porto, Portugal. | | Thesis | | Genetics | |
| Galantinho A, Mira A (2005) Influencia del uso del suelo y de la conservación de las líneas de agua en la abundancia de gineta (Genetta genetta L., 1758) en Monfurado, Portugal. In: *Resúmenes VII Jornadas de la SECEM*, Valencia, Spain. | | Conference Proceedings | | Conservation | |
| Galantinho A, Mira A (2009) The influence of human, livestock, and ecological features on the occurrence of genet (Genetta genetta): a case study on Mediterranean farmland. *Ecological Research* 24: 671–685. | | SCI Journals | | General ecology | |
| Gaubert P, Del Cerro I, Centeno-Cuadros A, Palomares F, Fournier P, Fonseca C, Paillat J-P, Godoy JA (2015) Tracing historical introductions in the Mediterranean Basin: the success story of the common genet (Genetta genetta) in Europe. *Biological Invasions*. | | SCI Journals | | Genetics | |
| Gaubert P, Godoy JA, del Cerro I, Palomares F (2009) Early phases of a successful invasion: mitochondrial phylogeography of the common genet (Genetta genetta) within the Mediterranean Basin. *Biological Invasions* 11: 523–546. | | SCI Journals | | Genetics | |
| Giesta P (2010) *Efeito da concentração de DNA nas taxas de erro de genotipagem de microssatélites em lobo: uma contribuição para estudos com amostras não invasivas.* MSc thesis, Faculty of Sciences of the University of Porto, Porto, Portugal. | | Thesis | | Genetics | |
| Godinho R (2013) New insights into the dynamics of hybridization between wolves and dogs. In: *Abstracts of the International Conference “Wolf Conservation in Human Dominated Landscapes”, 25-28 September 2013*, Oral. | | Conference Proceedings | | Genetics | |
| Godinho R, Llaneza L, Blanco JC, Lopes S, Álvares F, García EJ et al. (2011) Genetic evidence for multiple events of hybridization between wolves and domestic dogs in the Iberian Peninsula. *Molecular Ecology* 20: 5154–5166. | | SCI Journals | | Genetics | |
| Godinho R, Lopes S, Ferrand N (2007) *Estudo da diversidade e estruturação genética das populações de lobo (Canis lupus) em Portugal*. Research Center in Biodiversity and Genetic Resources/University of Porto, Porto, Portugal. | | Technical report | | Genetics | |
| Godinho R, López-Bao JV, Castro D, Llaneza L, Lopes S, Silva P, Ferrand N (2015) Real-time assessment of hybridization between wolves and dogs: combining noninvasive samples with ancestry informative markers. *Molecular Ecology Resources* 15: 317–28. | | SCI Journals | | Genetics | |
| Godinho R, Roque S, Castro D, Lopes S, Petrucci-Fonseca F, Álvares F (2012) Molecular ecology of the isolated and endangered wolf population occurring south of Douro river, Portugal. In: *Abstracts of the III Iberian Wolf Congress*, 21. Lugo, Spain. | | Conference Proceedings | | Genetics | |
| Gomes C (2010) *Comparação de dois métodos de monitorização de carnívoros. Armadilhagem de pêlo vs. Armadilhagem fotográfica.* MSc thesis, Faculty of Sciences of the University of Porto, Porto, Portugal. | | Thesis | | Others | |
| Gomes N (1997) Dieta de la nutria (Lutra lutra) en una zona húmeda del Baixo Vouga Lagunar (Ría de Aveiro), y su relación con la abundancia de presas. In: *Resúmenes III Jornadas de la SECEM*, Castelló d’Empúries, Gerona, Spain. | | Conference Proceedings | | General ecology | |
| Gomes N (1998) *Distribuição e ecologia trófica da lontra (Lutra lutra) na ria de Aveiro.* MSc thesis, Faculty of Sciences of the Unoversity of Porto, Porto, Portugal. | | Thesis | | Population Status | |
| Gomes N, Alves P, Henriques J, Ramos A, Tavares L (1989) Contribution à l’étude du régime alimentaire de la loutre (Lutra lutra) au Portugal. *Ciência Biológica. Ecology and Systematics* 9: 47–54. | | non-SCI Journals | | General ecology | |
| Gomes N, Silva E, Pópulo H, Leite M (1999) La utilización de rastros de Nutria (Lutra lutra) para el estudio de la comunidad de peces del Río Paiva (Cuenca del Río Duero, Portugal). In: *Resúmenes IV Jornadas de la SECEM*, Segovia, Spain. | | Conference Proceedings | | General ecology | |
| Gomes P (1988) *Análise da estrutura trófica de uma comunidade de mamíferos: estudo comparativo do regime alimentar de três tipos de predadores (raposa, geneta e complexo toirão/fuínha) no Parque Nacional da Peneda-Gerês.* University of Minho, Braga, Portugal. | | Thesis | | General ecology | |
| Gomes P (1998) *Ocupação e Utilização do Espaço pela Geneta.* PhD thesis, University of Minho, Braga, Portugal. | | Thesis | | General ecology | |
| Gonçalves A (2015) *Antibiotic resistance in gastrointestinal bacteria isolated from the Iberian lynx and Iberian wolf: species conservation and public health concerns.* PhD thesis, University of Trás-os-Montes and Alto Douro, Vila Real, Portugal. | | Thesis | | Health status | |
| Gonçalves A, Igrejas G, Radhouani H, Correia S, Pacheco R, Santos T et al. (2013) Antimicrobial resistance in faecal enterococci and Escherichia coli isolates recovered from Iberian wolf. *Letters in Applied Microbiology* 56: 268–74. | | SCI Journals | | Health status | |
| Gonçalves A, Poeta P, Monteiro R, Marinho C, Silva N, Guerra A et al. (2014) Comparative proteomics of an extended spectrum β-lactamase producing Escherichia coli strain from the Iberian wolf. *Journal of Proteomics* 104: 80–93. | | SCI Journals | | Health status | |
| Gonçalves I (2006) Espaços silvestres para animais selvagens, no noroeste de Portugal, com as inquirições de 1258. In: *Estudos em Homenagem ao Professor Doutor José Marques*, 193–219. Faculty of Arts of the University of Porto, Porto. | | Book | | Conservation | |
| Gonçalves P, Alcobia S, Simões L, Santos-Reis M (2011) Effects of management options on mammal richness in a Mediterranean agro-silvo-pastoral system. *Agroforestry Systems* 85: 383–395. | | SCI Journals | | Conservation | |
| Gonçalves S (2012) *Comparação da dieta da lontra (Lutra lutra) e do visão-americano (Neovison vison) numa situação de simpatria no NO de Portugal.* MSc thesis, Faculty of Sciences of the University of Lisboa, Lisbon, Portugal. | | Thesis | | General ecology | |
| Grilo AF (2013) *Influência de factores locais no uso das galerias ripícolas por carnívoros num ambiente mediterrânico.* MSc thesis, Faculty of Sciences of the University of Lisbon, Lisbon, Portugal. | | Thesis | | General ecology | |
| Grilo C (2009) *Habitat Connectivity for Carnivores in Mediterranean Landscapes: Implications for Conservation Planning.* PhD thesis, Faculty of Sciences of the University of Lisbon, Lisbon, Portugal. | | Thesis | | General ecology | |
| Grilo C, Ascensão F, Santos-Reis M, Bissonette JA. (2010) Do well-connected landscapes promote road-related mortality? European Journal of Wildlife Research 57: 707–716. | | SCI Journals | | Conservation | |
| Grilo C, Bissonette JA, Santos-Reis M (2008) Response of carnivores to existing highway culverts and underpasses: implications for road planning and mitigation. Biodiversity and Conservation 17: 1685–1699. | | SCI Journals | | Conservation | |
| Grilo C, Bissonette JA, Santos-Reis M (2009) Spatial-temporal patterns in Mediterranean carnivore road casualties: Consequences for mitigation. Biological Conservation 142: 301–313. | | SCI Journals | | Conservation | |
| Grilo C, Cândido AT, Alexandre AS, Moço G, Petrucci-Fonseca F (2002) The isolated Iberian wolf population in south Douro River (Portugal): Ecology and Threat factors. In: *Abstract of the International Congress of Biodiversity and Management*, Vouziers, Ardennes França. | | Conference Proceedings | | General ecology | |
| Grilo C, Cruz M, Bissonette JA, Santos-Reis M (2006) The role of patch size and isolation to carnivore relative abundance in a oak woodland landscape. In: Abstracts of the 1st European Congress on Conservation Biology, 114. Eger, Hungary. | | Conference Proceedings | | General ecology | |
| Grilo C, Ferreira FZ, Revilla E (2015) No evidence of a threshold in traffic volume affecting road-kill mortality at a large spatio-temporal scale. *Environmental Impact Assessment Review* 55: 54–58. | | SCI Journals | | Conservation | |
| Grilo C, Moço G, Alexandre A, Cândido AT, Petrucci-Fonseca F (1999) Integração de um modelo ambiental num sistema de informação geografica para a definição de corredores ecológicos. In: *Abstracts of the V Encontro sobre Sistemas de Informação Geografica.* Escola Superior Agrária de Castelo Branco, Castelo Branco, Portugal. | | Conference Proceedings | | Conservation | |
| Grilo C, Moço G, Cândido AT, Alexandre AS, Petrucci-Fonseca F (2001) Modelação de áreas prioritárias para a recuperação do lobo-ibérico a sul do rio Douro. In: *Proceedings of the VI Encontro Nacional de Ecologia - Fragmentação de Habitats e Populações*, Lisbon, Portugal. | | Conference Proceedings | | Conservation | |
| Grilo C, Moço G, Cândido AT, Alexandre AS, Petrucci-Fonseca F (2002) *Bases para a definição de corredores ecológicos na conservação de uma população marginal e fragmentada: o caso da população lupina a Sul do rio Douro – 1^a^ Fase*. Centre for Environmental Biology, Lisbon, Portugal. | | Technical report | | Conservation | |
| Grilo C, Moço G, Cândido AT, Alexandre AS, Petrucci-Fonseca F (2002) *Bases para a definição de corredores ecológicos na conservação de uma população marginal e fragmentada: o caso da população lupina a Sul do rio Douro – 2^a^ Fase*. Centre for Environmental Biology, Lisbon, Portugal. | | Technical report | | Conservation | |
| Grilo C, Moço G, Cândido AT, Alexandre AS, Petrucci-Fonseca F (2002) Challenges for the recovery of the Iberian wolf in the Douro river south region. *Revista de Biologia*: 121–133. | | non-SCI Journals | | Conservation | |
| Grilo C, Roque S, Rio-Maior H, Petrucci-Fonseca F (2003) El lobo ibérico al sur del Río Duero (Portugal): bases para la definición de una estrategia para su recuperación. In: *Resúmenes VI Jornadas de la SECEM*, 84. Ciudad Real, Spain. | | Conference Proceedings | | Conservation | |
| Grilo C, Roque S, Rio-Maior H, Petrucci-Fonseca F (2004) The isolated wolf population South of Douro River: status and action lines for its recovery. *Wolf Print* 20: 13–15. | | non-SCI Journals | | Conservation | |
| Grilo C, Santos-Reis M (2011) *Monitorização da mortalidade de vertebrados por atropelamento nas estradas de Portugal. 1º Relatório de Progresso*. Center for Environmental Biology, Faculty of Sciences of the University of Lisbon, Lisbon, Portugal. | | Technical report | | Conservation | |
| Grilo C, Silva C, Baltazar C, Gomes L, Bissonette JA, Santos-Reis M (2007) Patterns of Carnivore Road Casualties in Southern Portugal. In: *International Conference on Ecology and Transportation “Bridging The Gaps, Naturally,”* Little Rock, Arkansas, U.S.A. | | Conference Proceedings | | Conservation | |
| Grilo C, Sousa J, Ascensão F, Matos H, Leitão I, Pinheiro P et al. (2012) Individual Spatial Responses towards roads: Implications for Mortality Risk. *PLoS ONE* 7: e43811. | | SCI Journals | | Conservation | |
| Guerra AM (2004) *Estudo das relações ecológicas entre o lobo-ibérico e equinos e bovinos no Alto Minho: propostas para a minimização do impacto predatório.* Final Degree thesis, Faculty of Sciences of the University of Lisbon Lisbon, Portugal. | | Thesis | | General ecology | |
| Guerra D, Armua-Fernandez MT, Silva M, Bravo II, Santos N, Deplazes P, Carvalho LMM de (2013) Taeniid species of the Iberian wolf (Canis lupus signatus) in Portugal with special focus on Echinococcus spp. *International Journal for Parasitology: Parasites and Wildlife* 2: 50–53. | | SCI Journals | | Health status | |
| Guzman López-Ocón JN, García González FJ, Garrote Alonso G, Pérez de Ayala Balzola R, Iglesias Llamas C (2006) *El Lince Iberico (Lynx Pardinus) En España Y Portugal. Censo Diagnostico De Sus Poblaciones.* Dirección General para la Biodiversidad, Madrid, Spain. | | Book | | Population Status | |
| Haaften JL Van, Petrucci-Fonseca F, Pereira M (1983) A wolf study in Portugal. In: *Proceedings of the XVI Congress of Game Biologists*, 550–553. Strbské Pleso, Slovakia. | | Conference Proceedings | | General ecology | |
| Henriques JF, Tavares PC, Correia-dos-Santos MM, Trancoso MA, Santos-Reis M (2009) Contamination by heavy metals in aquatic trophic webs: the case of Otter and Red-Swamp Crayfish. In: *Abstracts of the 27th Mustelid Coloquium*, 50. Lisbon, Portugal. | | Conference Proceedings | | Health status | |
| Hipólito D (2014) *Influência das opções de gestão em recursos-chave para o texugo num ambiente agro-silvo-pastoril mediterrânico.* MSc thesis, Faculty of Sciences of the University of Lisbon, Lisbon, Portugal. | | Thesis | | Conservation | |
| Hodge I, Milheiras S (2011) Attitudes towards compensation for wolf damage to livestock in Viana do Castelo, North of Portugal. *Innovation: The European Journal of Social Science Research* 24: 333–351. | | non-SCI Journals | | Conservation | |
| Inácio Amaro AM, Felgueiras M, Lencastre M (2012) Representações e atitudes morais face ao lobo ibérico de alunos do ensino básico e secundário do concelho de Penafiel. In: *Abstracts of the III Iberian Wolf Congress*, 77. Lugo, Spain. | | Conference Proceedings | | Conservation | |
| Janeiro C, Ferreira JP, Mira A, Santos-Reis M (2007) Idoneidad y perspectivas de gestión de hábitat para el lince ibérico Lynx pardinus en el sitio Moura-Barrancos (Alentejo - Portugal). In: *Resúmenes VIII Jornadas de la SECEM*, Huelva, Spain. | | Conference Proceedings | | Conservation | |
| Jefferies R, Shaw SE, Willesen J, Viney ME, Morgan ER (2010) Elucidating the spread of the emerging canid nematode Angiostrongylus vasorum between Palaearctic and Nearctic ecozones. *Infection, Genetics and Evolution* 10: 561–568. | | SCI Journals | | Health status | |
| Jolibert C, Max-Neef M, Rauschmayer F, Paavola J (2011) Should We Care About the Needs of Non-humans? Needs Assessment: A Tool for Environmental Conflict Resolution and Sustainable Organization of Living Beings. *Environmental Policy and Governance* 21: 259–269. | | SCI Journals | | Conservation | |
| Lampa S, Gruber B, Halle S, Santos-Reis M, Henle K (2009) Distribution and Habitat Preferences of the American Mink (Mustela vison) in Portugal. In: *Abstracts of the 27th Mustelid Coloquium*, 32. Lisbon, Portugal. | | Conference Proceedings | | General ecology | |
| Lançós JP (1999) *Contribuição para o conhecimento da ecologia do lobo no Parque Nacional da Peneda-Gerês.* Faculty of Sciences of the University of Porto, Porto, Portugal. | | Thesis | | General ecology | |
| Leandre E (2000) *Research concerning the analysis of the otter’s diet (Lutra lutra) and marking behaviour in Grândola Mountain, Portugal*. Van Hall Instituut, Leeuwarden, Netherlands. | | Technical report | | General ecology | |
| Lecoq M, Santos E, Mouro C, Castro P, Allcorn R, Loureiro F (2007) Proyecto Life-Naturaleza recuperación del hábitat del lince ibérico en el Sitio Moura/Barrancos. In: *Resúmenes VIII Jornadas de la SECEM*, Huelva, Spain. | | Conference Proceedings | | Conservation | |
| Lopes AP, Sargo R, Rodrigues M, Cardoso L (2011) High seroprevalence of antibodies to Toxoplasma gondii in wild animals from Portugal. *Parasitology Research* 108: 1163–1169. | | SCI Journals | | Health status | |
| Lopes AP, Vila-Viçosa MJ, Coutinho T, Cardoso L, Gottstein B, Müller N, Cortes HCE (2015) Trichinella britovi in a red fox (Vulpes vulpes) from Portugal. *Veterinary parasitology* 210: 260–3. | | SCI Journals | | Health status | |
| Lopes M (1998) Utilização do Rio Guadiana e dos seus afluentes pela Lontra (Lutra lutra Linnaeus, 1758) na Área do Parque Natural do Vale do Guadiana. Instituto da Conservação da Natureza/Parque Natural do Vale do Guadiana/ INTERREG II. | | Technical report | | General Ecology | |
| Lopes M, Medeiros F (2012) Conservação dos Vertebrados Terrestres de São Jorge. Relatórios e Comunicações do Departamento de Biologia, 40: 89-95. | | Technical report | | Conservation | |
| Loureiro C (2008) *Time and Space Use of Key Resources by the Eurasian badger (Meles meles) in a Mediterranean Cork Oak Woodland: Conservation Implications.* PhD thesis, Faculty of Sciences of the University of Lisbon, Lisbon, Portugal. | | Thesis | | General ecology | |
| Loureiro C, Mendes L, Oliveira J, Brotas G (2012) Medidas de compensação para o lobo: análise da experiência de cinco anos com parques eólicos. In: *Abstracts of the III Iberian Wolf Congress*, 66. Lugo, Spain. | | Conference Proceedings | | Conservation | |
| Loureiro F (1999) *Aspectos da ecologia do texugo Meles meles, L. na Serra de Grândola.* Final Degree thesis, Faculty of Sciences of the University of Lisbon Lisbon, Portugal. | | Thesis | | General ecology | |
| Loureiro F, Bissonette JA, Macdonald DW, Santos-Reis M (2006) Pulsed resources in a Mediterranean landscape and their importance to Eurasian badgers conservation. In: Abstracts of the 1st European Congress on Conservation Biology, 134. Eger, Hungary. | | Conference Proceedings | | General ecology | |
| Loureiro F, Bissonette JA, Macdonald DW, Santos-Reis M (2009) Temporal Variation in the Availability of Mediterranean Food Resources: Do Badgers Meles meles Track Them? Wildlife Biology 15: 197–206. | | SCI Journals | | General ecology | |
| Loureiro F, Martins AR (2011) Lynx Programme’s contribute through LIFE Nature Programme in wild rabbit's recovery. In: *Abstracts of the II International European Rabbit Congress*, Toledo, Spain. | | Conference Proceedings | | Conservation | |
| Loureiro F, Martins AR, Santos E, Lecoq M, Emauz A, Pedroso NM, Hotham P (2011) O Papel do Programa Lince (LPN/FFI) na Recuperação do Habitat e Presas do Lince-Ibérico no Sul de Portugal. *Galemys* 23: 17–25. | | non-SCI Journals | | Conservation | |
| Loureiro F, Rosalino LM, Macdonald DW, Santos-Reis M (2007) Path tortuosity of Eurasian badgers (Meles meles) in a heterogeneous Mediterranean landscape. *Ecological Research* 22: 837–844. | | SCI Journals | | General ecology | |
| Loureiro F, Rosalino LM, Macdonald DW, Santos-Reis M (2007) Use of multiple den sites by Eurasian badgers, Meles meles, in a Mediterranean habitat. *Zoological Science* 24: 978–985. | | SCI Journals | | General ecology | |
| Loureiro F, Rosalino LM, Santos-Reis M, Macdonald DW (1999) Hábitos alimenticios del Tejón en Portugal: una primera aproximación. In: *Resúmenes IV Jornadas de la SECEM*, Segovia, Spain. | | Conference Proceedings | | General ecology | |
| Loureiro F, Santos-Reis M (2005) Vídeo vigilancia en estudios ecológicos: aplicación al estudio del tejón eurasiático (Meles meles). In: *Resúmenes VII Jornadas de la SECEM*, Valencia, Spain. | | Conference Proceedings | | General ecology | |
| Loureiro F, Sousa M, Basto MP, Pedroso NM, Rosário J, Sales-Luís T, Chambel I, Rosalino LM (2007) A comunidade de mamíferos não voadores da Paisagem Protegida da Serra de Montejunto (Centro de Portugal): Distribuição e situação regional. Galemys 19: 139–157. | | non-SCI Journals | | Population Status | |
| Loureiro S (2013) *Enriquecimento ambiental num núcleo cativo de lobo (Canis lupus).*  MSc thesis, Faculty of Sciences of the University of Lisbon, Lisbon, Portugal. | | Thesis | | Conservation | |
| Lourenço A (2013) *Combining movement and genetic data to assess a forest carnivore’s response to forest fragmentation.*  MSc thesis, Faculty of Sciences of the University of Porto, Porto, Portugal. | | Thesis | | General ecology | |
| Lourenço S (1999) *Ecologia espacial de Genetta genetta e Martes foina na Serra de Grândola.* Final Degree thesis, Faculty of Sciences of the University of Lisbon Lisbon, Portugal. | | Thesis | | General ecology | |
| Lousada HR (2006) *Monitorização de prejuízos do Lobo-ibérico na área de influência do Parque Natural do Alvão: relatório final de projecto.* Vila Real, Portugal. | | Technical report | | Conservation | |
| Lyle R (1988) *The wolf in Portugal.* Eurogroup for Animal Welfare (Unpublished report) | | Technical report | | Population Status | |
| MacDonald SM, Mason CF (1982) The otter Lutra lutra in central Portugal. *Biological Conservation* 22: 207–215. | | SCI Journals | | Population Status | |
| Magalhães CP (1974) *Hábitos alimentares da Raposa (Vulpes vulpes silacea) e da Geneta (Genetta genetta) na Tapada de Mafra*. Direcção-Geral dos Serviços Florestais e Aquícolas. | | Technical report | | General ecology | |
| Magalhães CP (1975) Some features of the wolf (Canis lupus signatus Cabrera, 1907) in Portugal. In: *Actas do XII Congresso da União Internacional dos Biologistas da Caça*, 303–309. Lisbon, Portugal. | | Conference Proceedings | | Population Status | |
| Magalhães CP (1984) *Aspectos do lobo (Canis lupus L.) em Portugal*. Ministério da Agricultura, Florestas e Alimentação - D.G.F. - Biologia e Ordenamento Cinegético. | | Technical report | | Population Status | |
| Magalhães CP, Palma L (1985) Present Status of Portuguese Predators. In: *Symposium Predateurs*, 241–278. Conseil International de la Chasse et la Conservation du Gibier, Lisbon, Portugal. | | Conference Proceedings | | Population Status | |
| Magalhães CP, Petrucci-Fonseca F (1982) The wolf in Bragança County: Impact on cattle and game. In: *Transactions of the XIV Congress of the International Union of Game Biologists*, 281–286. Dublin, Ireland. | | Conference Proceedings | | Conservation | |
| Magalhães MF, Beja P, Canas C, Collares-Pereira MJ (2002) Functional heterogeneity of dry-season fish refugia across a Mediterranean catchment: the role of habitat and predation. Freshwater Biology 47: 1919–1934. | | SCI Journals | | General ecology | |
| Malveiro E (2007) *Estudo Genético do Lobo Ibérico. Utilização de microssatélites no estudo populacional, na identificação individual e de relações de parentesco.* MSc thesis, Faculty of Sciences of the University of Lisboa, Lisbon, Portugal. | | Thesis | | Genetics | |
| Marques C (2003) *Distribuição, densidade e utilização sazonal de locais de repouso diurno de texugo (Meles meles, L.) na Serra de Grândola.* Final Degree thesis, Faculty of Sciences of the University of Lisbon Lisbon, Portugal. | | Thesis | | General ecology | |
| Marques C, Rosalino LM, Santos-Reis M (2007) Otter predation in a trout fish farm of Central-east Portugal: Preference for “fast-food”? *River Research and Applications* 23: 1147–1153. | | SCI Journals | | General ecology | |
| Marques É (2011) *Aplicação da análise da composição isotópica no esclarecimento da origem das presas consumidas pela lontra numa área de pisciculturas.* MSc thesis, Faculty of Sciences of the University of Lisbon, Lisbon, Portugal. | | Thesis | | General ecology | |
| Marques T (1999) *Padrão de actividade e movimentos de Martes foina e Genetta genetta na Serra de Grândola.* Final Degree thesis, Faculty of Sciences of the University of Lisbon Lisbon, Portugal. | | Thesis | | General ecology | |
| Martins HMC (1996) *Modelos estatísticos de Avaliação do Habitat: um exercício metodológico com três espécies cinegéticas.* Instituto Superior de Agronomia, University of Lisbon, Lisbon, Portugal. | | Thesis | | General ecology | |
| Masseti M (2010) Mammals of the Macaronesian islands (the Azores, Madeira, the Canary and Cape Verde islands): redefinition of the ecological equilibrium. *Mammalia* 74: 3–34. | | SCI Journals | | General ecology | |
| Mate I, Barrull J, Gosálbez J, Ruiz-Olmo J, Salicrú M (2014) The role of the southern water vole A rvicola sapidus in the diet of predators: a review. *Mammal Review*. Advance online publication | | SCI Journals | | *General Ecology* | |
| Mateus AR (2008) *Passagens hidráulicas das auto-estradas: comparação de métodos de monitorização e avaliação dos factores que promovem a sua utilização pelos carnívoros.* MSc thesis, Faculty of Sciences of the University of Lisbon, Lisbon, Portugal. | | Thesis | | Others | |
| Mateus AR, Grilo C, Santos-Reis M (2011) Surveying drainage culvert use by carnivores: sampling design and cost-benefit analyzes of track-pads vs. video-surveillance methods. *Environmental Monitoring Assessment* 181: 101–109. | | non-SCI Journals | | Conservation | |
| Mateus TL, Barrocas C (2012) Diversidade e prevalência de helmintes intestinais encontrados em fezes de Genetta genetta numa área protegida do Norte de Portugal - dados preliminares. In: *Abstracts of the IV Congresso da Fauna Selvagem WAVES Portugal*, 48. Bragança, Portugal. | | Conference Proceedings | | Health status | |
| Mathias ML (1988) An annotated list of the mammals recorded from the Madeira islands. *Boletim do Museu Municipal do Funchal,* 40: 111–137. | | non-SCI Journals | | Population Status | |
| Mathias ML, Ramalhinho MG, Santos-Reis M, Petrucci-Fonseca F, Libois R, Fons R, Ferraz De Carvalho G, Oom MM, Collares-Pereira MJ (1998) Mammals from the Azores islands (Portugal): an updated overview. *Mammalia* 62: 397–407. | | SCI Journals | | Population Status | |
| Mathias ML, Santos-Reis M, Palmeirim J, Ramalhinho MG (1998) Mamíferos de Portugal. Edições Inapa, Lisbon. | | Book | | Population Status | |
| Matos AC, Figueira L, Martins MH, Loureiro F, Pinto ML, Matos M, Coelho AC (2014) Survey of Mycobacterium avium subspecies paratuberculosis in road-killed wild carnivores in Portugal. *Journal of Zoo and Wildlife Medicine* 45: 775–781. | | SCI Journals | | Health status | |
| Matos AC, Figueira L, Martins MH, Matos M, Álvares S, Pinto ML, Coelho AC (2013) Disseminated Mycobacterium avium subsp. paratuberculosis infection in two wild Eurasian otters (Lutra lutra L.) from Portugal. *Journal of Zoo and Wildlife Medicine* 44: 193–195. | | SCI Journals | | Health status | |
| Matos AC, Figueira L, Martins MH, Matos M, Morais M, Dias AP, Pinto ML, Coelho AC (2014) Disseminated Mycobacterium bovis infection in red foxes (Vulpes vulpes) with cerebral involvement found in Portugal. *Vector Borne and Zoonotic Diseases* 14: 531–533. | | SCI Journals | | Health status | |
| Matos AC, Figueira L, Martins MH, Matos M, Pires MA, Álvares S et al. (2013) Diffuse Lymphadenitis and Disseminated Mycobacterium avium subsp. paratuberculosis Infection in Two Wild Eurasian Otters (Lutra lutra L. 1758). *Journal of Comparative Pathology* 148: 85. | | SCI Journals | | Health status | |
| Matos AC, Figueira L, Martins MH, Matos M, Pires MA, Coelho AC, Pinto ML (2013) Pulmonary lesions consistent with disseminated adiaspiromycosis in Egyptian Mongooses (Herpestes ichneumon) from Portugal. Journal of Comparative Pathology 148: 87–87. | | SCI Journals | | Health status | |
| Matos AC, Figueira L, Martins MH, Pinto ML, Matos M, Coelho AC (2014) New Insights into Mycobacterium bovis Prevalence in Wild Mammals in Portugal. *Transboundary and Emerging Diseases.* Advance online publication | | SCI Journals | | Health status | |
| Matos H (1999) *Aspectos da ecologia da lontra (Lutra lutra) em ribeiras intermitentes num sector da bacia do rio Sado (SO Portugal).* Final Degree thesis, Faculty of Sciences of the University of Lisbon Lisbon, Portugal. | | Thesis | | General ecology | |
| Matos H (2006) Riparian Habitat Use By Mammalian Carnivores in Two Contrasting Mediterranean Matrix Landscapes in Southwestern Portugal. In: Delach A (ed) *Abstracts of the Defenders of Wildlife’s Carnivores 2006 - Habitat, Challenges and Opportunities*, 187. St. Petersburg, Florida, U.S.A. | | Conference Proceedings | | General ecology | |
| Matos H, Basto MP, Santos-Reis M (2009) Riparian habitat influence on habitat use and home range of Martes foina in Mediterranean ecossystems. In: Abstracts of the 27th Mustelid Coloquium, 56. Lisbon, Portugal. | | Conference Proceedings | | General ecology | |
| Matos H, Santos MJ, Grilo C, Sousa I, Santos-Reis M (2001) *Estudos de biologia e ecologia do toirão Mustela putorius na área de regolfo de Alqueva e Pedrógão. Centre for Environmental Biology, Faculty of Sciences of the University of Lisbon* Lisbon, Portugal. | | Technical report | | General ecology | |
| Matos H, Santos-Reis M (2001) Trophic Ecology of Lutra lutra in Intermittent Streams of SW Protugal. In: *Proceedings of the VIIIth International Otter Colloquium*, Valdivia, Chile. | | Conference Proceedings | | General ecology | |
| Matos H, Santos-Reis M (2003) *Distribuição actual e abundância relativa de Martes martes e Mustela putorius. Relatório Final. Estudo integrado no Projecto do ICN “Livro Vermelho dos Vertebrados de Portugal – Revisão”, Programa Operacional do Ambiente.* Centro de Biologia Ambiental (FCUL), Lisboa, Portugal. | | Technical report | | Population Status | |
| Matos H, Santos-Reis M (2006) Distribution and status of the pine marten Martes martes in Portugal. In: Santos-Reis M, Birks JDS, O’Doherty EC, Proulx G (eds) *Martes in Carnivore Communities: Proceedings of the Fourth International Martes Symposium*, 45–65. Alpha Wildlife Publications, Alberta, Canada. | | Book | | Population Status | |
| Matos HM, Santos MJ, Palomares F, Santos-Reis M (2009) Does riparian habitat condition influence mammalian carnivore abundance in Mediterranean ecosystems? *Biodiversity and Conservation* 18: 373–386. | | SCI Journals | | General ecology | |
| Matos M, Soares A, Morgado F, Fonseca C (2007) Mastofauna del bosque nacional de Buçaco, Centro de Portugal. *Galemys* 19: 45–59. | | non-SCI Journals | | Population Status | |
| Medeiros F, Mântua P, Portela N, Furtado M, Magalhães B, Teixeira B, Fonseca A (2010) *Conservação dos vertebrados terrestres na ilha de Santa Maria*. University of Açores, Ponta Delgada, Portugal. | | Technical report | | Conservation | |
| Melo Cepêda Aires JA, Miranda C, Silva E, Álvares F, Thompson G (2012) Estudo preliminar para a avaliação da incidência de Parvovírus Canino na população portuguesa de lobo: potencialidades e limitações. In: *Abstracts of the III Iberian Wolf Congress*, 58. Lugo, Spain. | | Conference Proceedings | | Health status | |
| Mendes M (2012) *A gruta da cerca do Zambujal: uma necrópole do Neolítico final.* MSc thesis, Universidade Nova de Lisboa, Lisbon, Portugal. | | Thesis | | Others | |
| Mendes R (2011) *Resposta da comunidade de carnívoros à invasão dos sistemas ribeirinhos do NO de Portugal por lagostim-vermelho (Procambarus clarkki).* MSc thesis, Faculty of Sciences of the University of Lisboa, Lisbon, Portugal. | | Thesis | | General ecology | |
| Mendes R, Rodrigues D, Moreira F, Mullins J, Rebelo R, Santos-reis M (2011) Dietary shifts of the otter in a recently invaded area by the American crayfish. In: *Abstracts of the* *29th European Mustelid Colloquium*, Southampton, England. | | Conference Proceedings | | General ecology | |
| Mendes T (2014) *Adequabilidade da Charneca do Infantado para o gato-bravo (Felis silvestris) e influência da presença do gato doméstico (Felis catus).* MSc thesis, Faculty of Sciences of the University of Lisbon, Lisbon, Portugal. | | Thesis | | Conservation | |
| Mertens A, Angelucci S, Cortés Y, Di Nicola U, Huber D, Latini R, Ribeiro S, Convito L (2006) Carnivore-livestock conflicts and their management – a comparison across five Mediterranean countries. In: *Abstracts of the 1st European Congress on Conservation Biology*, 138. Eger, Hungary. | | Conference Proceedings | | Conservation | |
| Mestre FM, Ferreira JP, Mira A (2007) Modelling the distribution of the European polecat Mustela putorius in a Mediterranean agricultural landscape. *Revue d’Ecologie, La Terre et la Vie* 62: 35–47. | | non-SCI Journals | | General ecology | |
| Mestre H (2012) *The effect of habitat fragmentation in the diet of pine marten.* MSc thesis, Faculty of Sciences of the University of Lisbon, Lisbon, Portugal. | | Thesis | | General ecology | |
| Mira A (1995) *Inventariação/caracterização dos mamíferos do Parque Natural da Serra de S. Mamede.* Instituto de Conservação da Natureza | | Technical report | | Population Status | |
| Mira A, Costa M, Manghi G (2005) Área vital y patrones de actividad del turón en el sur de Portugal. Datos preliminares. In: *Resúmenes VII Jornadas de la SECEM*, Valencia, Spain. | | Conference Proceedings | | General ecology | |
| Mira A, Galantinho A, Silva A, Ascensão F, Mestre FM, Godinho R (2004) Caracterização da Fauna terrestre da zona de implantação do futuro Aeroporto a construir na OTA, e envolvente próxima. Instituto de Oceanografia/Faculty of Sciences of the University of Lisbon. | | Technical report | | Population Status | |
| Monteiro MA (1998) *Habitat e distribuição do lince-ibérico (Lynx pardinus Temminck, 1824) no Vale do Sado: implicações de conservação.* Faculty of Sciences of the University of Lisbon, Lisbon, Portugal. | | Thesis | | General ecology | |
| Monterroso P (2004) *Distribuição, selecção de habitat e actividade do gato-bravo (Felis silvestris) no Parque natural do Vale do Guadiana.* MSc thesis, Faculty of Sciences of the University of Porto, Porto, Portugal. | | Thesis | | General ecology | |
| Monterroso P (2013) *Ecological Interactions and Species Coexistence in Iberian Mesocarnivore Communities.* PhD thesis, Faculty of Sciences of the University of Porto, Porto, Portugal. | | Thesis | | General ecology | |
| Monterroso P, Alves PC, Ferreras P (2011) Evaluation of attractants for non-invasive studies of Iberian carnivore communities. *Wildlife Research* 38: 446. | | SCI Journals | | Others | |
| Monterroso P, Alves PC, Ferreras P (2013) Catch Me If You Can: Diel Activity Patterns of Mammalian Prey and Predators (L Fusani, Ed). *Ethology* 119: 1044–1056. | | SCI Journals | | General ecology | |
| Monterroso P, Alves PC, Ferreras P (2014) Plasticity in circadian activity patterns of mesocarnivores in Southwestern Europe: implications for species coexistence. *Behavioral Ecology and Sociobiology* 68: 1403–1417. | | SCI Journals | | General ecology | |
| Monterroso P, Brito J, Ferreras P, Alves PC (2009) Spatial ecology of the European wildcat in a Mediterranean ecosystem: dealing with small radio-tracking datasets in species conservation. *Journal of Zoology* 279: 27–35. | | SCI Journals | | General ecology | |
| Monterroso P, Castro D, Silva TL, Ferreras P, Godinho R, Alves PC (2013) Factors affecting the (in)accuracy of mammalian mesocarnivore scat identification in South-western Europe. *Journal of Zoology* 289: 243–250. | | SCI Journals | | Others | |
| Monterroso P, Ferreras P, Brito JC, Alves PC (2006) Combining camera-trapping and radiotelemetry methods to define ecological requirements for the european wildcat (F. silvestris) in a Mediterranean ecosystem. In: *Abstracts of the 1st European Congress on Conservation Biology*, 53. Eger, Hungary. | | Conference Proceedings | | General ecology | |
| Monterroso P, Rich LN, Serronha A, Ferreras P, Alves PC (2013) Efficiency of hair snares and camera traps to survey mesocarnivore populations. *European Journal of Wildlife Research* 60: 279–289. | | SCI Journals | | Conservation | |
| Monterroso P, Sarmento P, Brito JC, Ferreras P, Alves PC (2005) Aplicación de trampeo fotográfico para desarrollar un modelo de abundancia para el gato montés (Felis silvestris) en el sur de Portugal: definición de prioridades para su conservación. In: *Resúmenes VII Jornadas de la SECEM*, Valencia, Spain. | | Conference Proceedings | | Conservation | |
| Monterroso P, Sarmento P, Ferreras P, Alves PC (2005) Spatial distribution of the European wildcat (Felis silvestris) in Vale do Guadiana Natural Park, South Portugal. In: *Abstracts of the Symposium: Biology and Conservation of the European Wildcat (Felis silvestris silvestris)*, 17. Vosges, Germany. | | Conference Proceedings | | Population Status | |
| Moreira L (1992) *Contribuição para o estudo da ecologia do lobo (Canis lupus signatus Cabrera, 1907) no Parque Natural de Montesinho*. Final Degree thesis, Faculty of Sciences of the University of Lisbon Lisbon, Portugal. | | Thesis | | General ecology | |
| Moreira L (1998) *O lobo no Nordeste de Trás-os-Montes.* Azevedo, J. (Ed). Mirandela. | | Book | | Conservation | |
| Moreira L (2012) 20 anos de Conservação do lobo no Nordeste de Portugal. In: *Abstracts of the IV Congresso da Fauna Selvagem WAVES Portugal*, 39. Bragança, Portugal. | | Conference Proceedings | | Conservation | |
| Moreira L, Rosa JL, Lourenço J, Barroso I, Pimenta V (1997) *Projecto Lobo - Relatório de Progressão 1996*. Instituto da Conservação da Natureza, Bragança, Portugal. | | Technical report | | Conservation | |
| Moreira L, Rosa JL, Lourenço J, Pimenta V, Kelm D (1997) Análise dos prejuízos causados por lobo (Canis lupus) nos animais domésticos no distrito de Bragança. In: *Resúmenes I Congreso Hispano-Luso. Situación y conservación de las poblaciones de lobo en la Península Ibérica, Soria, Spain.* | | Conference Proceedings | | Conservation | |
| Moreno-García M, Pimenta C, Ruas JP (2003) *Contribuição para o estudo das populações actuais de Lobo ibérico (Canis lupus signatus) em Portugal através dos seus ossos. Estudo de treze exemplares.* Humana e Arqueociências (CIPA) do Instituto Português de Arqueologia, Lisbon, Portugal. | | Technical report | | Population Status | |
| Moreno-García M, Pimenta C, Silva A, Ruas JP (2005) *Contribuição para o estudo das populações actuais de Lobo ibérico (Canis lupus signatus) em Portugal através dos seus ossos. Estudo de seis exemplares*. Centro de Investigação em Paleoecologia Humana e Arqueociências (CIPA) do Instituto Português de Arqueologia, Lisbon, Portugal. | | Technical report | | Population Status | |
| Morgado M (2014) *Estudo comportamental da espécie lontra-europeia (Lutra lutra) em cativeiro: a situação no parque biológico de Gaia.* MSc thesis, Abel Salazar Institute of Biomedical Sciences, University of Porto, Porto, Portugal. | | Thesis | | General ecology | |
| Mucci N, Arrendal J, Ansorge H, Bailey M, Bodner M, Delibes M et al. (2010) Genetic diversity and landscape genetic structure of otter (Lutra lutra) populations in Europe. *Conservation Genetics* 11: 583–599. | | SCI Journals | | Genetics | |
| Mueller A, Silva E, Santos N, Thompson G (2011) Domestic Dog Origin of Canine Distemper Virus in Free-ranging Wolves in Portugal as Revealed by Hemagglutinin Gene Characterization. *Journal of Wildlife Diseases* 47: 725–729. | | SCI Journals | | Health status | |
| Nakamura M (2011) Utilización de Índices de Abundancia de indicios para la monitorización del lobo (Canis lupus): potencialidades y validación con Telemetría GPS *Resúmenes X Congreso de la SECEM*, 98. Fuengirola, Málaga, Spain. | | Conference Proceedings | | Conservation | |
| Nakamura M, Godinho R, Rio-Maior H, Roque S, Bernardo J, Castro D, Lopes S, Petrucci-Fonseca F, Álvares F (2012) Como potenciar o sucesso na extração de DNA, identificação específica e individual em amostras não-invasivas de lobo?. In: *Abstracts of the III Iberian Wolf Congress*, 48. Lugo, Spain. | | Conference Proceedings | | Genetics | |
| Nakamura M, Rio-Maior H, Godinho R, Álvares F (2013) *Investigação Aplicada à Conservação do Lobo no Noroeste de Portugal. Relatório Técnico – Ano II.* CIBIO-UP/Grupo Lobo, 56pp+Anexos. | | Technical report | | Conservation | |
| Negrões N, Carvalho JC, Gomes PA (2001) The Diet of Red Fox (Vulpes vulpes) In Two Areas of Parque Nacional da Peneda-Gerês (NW Portugal). In: *Abstracts of the Canid Biology & Conservation: An International Conference*, 53. Oxford, U.K. | | Conference Proceedings | | General ecology | |
| Neto de Carvalho C (2014) Footprints of Iberian lynx (Lynx pardinus) in the Upper Pleistocene from Pessegueiro Island (Portugal). *Comunicações Geológicas* 101: 513–516. | | non-SCI Journals | | Others | |
| Neukirchen B, Niethammer J (1992) Igel (Erinaceus euro- paeus) und andere Säugetiere auf den Azoren. *Säugetierk. Mitt* 34: 59–63. | | non-SCI Journals | | Population Status | |
| Neves MA, Albuquerque T, Botelho A, Monteiro M, Carvalho P, Mendonça P et al. (2012) Tuberculose em Carnívoros Silvestres: hipóteses, evidências e implicações na Conservação. In: *Abstracts of the IV Congresso da Fauna Selvagem WAVES Portugal*, 26. Bragança, Portugal. | | Conference Proceedings | | Health status | |
| Novais A, Sedlmayr A, Moreira-Santos M, Goncalves F, Ribeiro R (2010) Diet of the otter Lutra lutra in an almost pristine Portuguese river: seasonality and analysis of fish prey through scale and vertebrae keys and length relationships. *Mammalia* 74: 71–81. | | SCI Journals | | General ecology | |
| Nunes L, Gomes AL, Fonseca A (2015) Wildlife corridors based on the spatial modeling of the human pressure: A Portuguese case study. In: Watson A, Carver S , Krenova Z, McBride B (eds) *USDA Forest Service Proceedings RMRS-P-74*, 5–13. Department of Agriculture, Forest Service, Rocky Mountain Rese, Fort Collins, CO: U.S. | | Conference Proceedings | | Conservation | |
| Nunes M, Nunes J (2004) A situação do lobo na Serra da Aboboreira: resultados do Projecto LOBO, um Projecto de Educação Ambiental. In: Nunes M (ed) *Serra da Aboboreira: a Terra, o Homem e os Lobos*, 103–121. Câmara Municipal de Amarante. | | Book | | Population Status | |
| Ogando J (1996) *Estudo da influência das condições de cativeiro no comportamento social do lobo ibérico (Canis lupus signatus Cabrera, 1907).* Final Degree thesis, Faculty of Sciences of the University of Lisbon Lisbon, Portugal. | | Thesis | | Others | |
| Oliveira H (2010) *Análise espacial do habitat do lobo no Noroeste de Portugal.*  Master thesis, School of Statistics and Information Management, Universidade Nova de Lisboa, Lisboa, Portugal. | | Thesis | | General ecology | |
| Oliveira LF (1985) *Prospecções de campo para a detecção do Lince em parte da região do Baixo Sado.* | | Technical report | | Population Status | |
| Oliveira M, Pedroso NM, Sales-Luís T, Santos-Reis M, Tavares L, Vilela CL (2009) Evidence of antimicrobial resistance in Eurasian otter (Lutra lutra Linnaeus, 1758) fecal bacteria in Portugal. In: Harris J, Brown PL (eds) Wildlife: Destruction, Conservation and Biodiversity, 201–221. Nova Science Publishers. | | Book | | Health status | |
| Oliveira M, Pedroso NM, Sales-Luís T, Santos-Reis M, Tavares L, Vilela CL (2010) Antimicrobial-resistant Salmonella isolated from Eurasian otters (Lutra lutra Linnaeus, 1758) in Portugal. *Journal of Wildlife Diseases* 46: 1257–1261. | | SCI Journals | | Health status | |
| Oliveira M, Ribeiro T (2011) Antimicrobial Resistant Aeromonas Isolated from Eurasian Otters (Lutra lutra Linnaeus , 1758) in Portugal. In: Gupta VK, Varma AK (eds) *Animal Diversity, Natural History and Conservation*, 123–143. Daya Publishing House. | | Book | | Health status | |
| Oliveira M, Sales-Luís T, Duarte A, Nunes SF, Carneiro C, Tenreiro T et al. (2008) First assessment of microbial diversity in faecal microflora of Eurasian otter (Lutra lutra Linnaeus, 1758) in Portugal. *European Journal of Wildlife Research* 54: 245–252. | | SCI Journals | | Health status | |
| Oliveira R, Castro D, Godinho R, Luikart G, Alves PC (2010) Species identification using a small nuclear gene fragment: application to sympatric wild carnivores from South-western Europe. *Conservation Genetics* 11: 1023–1032. | | SCI Journals | | Genetics | |
| Oliveira R, Godinho R, Pierpaoli M, Randi E, Ferrand N, Alves PC (2005) Genetic diversity of portuguese wildcat (Felis silvestris) populations and detection of hybridization with domestic cats. In: *Abstracts of the Symposium: Biology and Conservation of the European Wildcat (Felis silvestris silvestris)*, 14. Vosges, Germany. | | Conference Proceedings | | Genetics | |
| Oliveira R, Godinho R, Randi E, Alves PC (2007) The Promise of SNPs (Single Nucleotide Polymorphisms) for Wildcat Conservation: Detecting Hybridisation with Domestic Cats. In: Hughes J, Mercer R (eds) *Abstracts of the Felid Biology and Conservation Conference*, 102. Oxford University, Oxford, U.K. | | Conference Proceedings | | Genetics | |
| Oliveira R, Godinho R, Randi E, Alves PC (2008) Hybridization versus conservation: are domestic cats threatening the genetic integrity of wildcats (Felis silvestris silvestris) in Iberian Peninsula? *Philosophical Transactions of the Royal Society B: Biological Sciences* 363: 2953–2961. | | SCI Journals | | Genetics | |
| Oliveira R, Godinho R, Randi E, Ferrand N, Alves PC (2008) Molecular analysis of hybridisation between wild and domestic cats (Felis silvestris) in Portugal: implications for conservation. *Conservation Genetics* 9: 1–11. | | SCI Journals | | Genetics | |
| Oliveira T, Carmo P (2000) Distribuição das Principais Presas Selvagens do Lobo Ibérico (Canis lupus signatus Cabrera 1907) a Norte do Rio Douro. *Galemys* 12: 257–268. | | non-SCI Journals | | General ecology | |
| Oliveira TM, Santos-Reis M (1993) Variabilidad genética y morfológica del zorro (Vulpes vulpes silacea Miller, 1907) en Portugal. In: *Resúmenes I Jornadas de la SECEM*, Molina, Málaga, Spain. | | Conference Proceedings | | Genetics | |
| Osojnik N, Nakamura M, Rio-Maior H, Álvares F (2013) Social Behaviour of Iberian wolf packs: a preliminary study based on footage obtained in the wild. In: *Abstracts of the International Conference “Wolf Conservation in Human Dominated Landscapes”, 25-28 September 2013*, (Poster). Postojna, Slovenia. | | Conference Proceedings | | General ecology | |
| Palacios V, Font E, Márquez R (2003) A quantitative description of the Iberian Wolf Howls. In: *Abstracts of the World Wolf Congress 2003: Bridging Science and Community*, 78. Banff, Alberta, Canada. | | Conference Proceedings | | General ecology | |
| Palma L (1977) *Contribuição para o estudo da biologia do lince-ibérico, Lynx pardina (Temminck, 1824) e da sua conservação na Serra da Malcata.* Faculty of Sciences of the University of Lisbon, Lisbon, Portugal. | | Thesis | | Conservation | |
| Palma L (1978) Sobre a distribuição, ecologia e conservação do lince ibérico em Portugal. In: *Actas I Reunion Iberoamericana de Zoólogos de Vertebrados*, 569–586. Huelva, Spain. | | Conference Proceedings | | Conservation | |
| Palma L (1981) Os últimos linces de Portugal. 1.^a^ Parte - História, Distribuição e Regressão do Lince na Península Ibérica. *Diana - Revista Mensal de Caça e Pesca* 16: 39–53. | | non-SCI Journals | | Population Status | |
| Palma L (1981) Os últimos linces de Portugal. 2.^a^ Parte - Habitat, biologia e ecologia. *Diana - Revista Mensal de Caça e Pesca* 17: 35–39. | | non-SCI Journals | | General ecology | |
| Palma L (1995) *Bases para uma estrategia de conservação do Lince Ibérico Lynx pardinus no interior do Algarve e sudoeste do Alentejo*. Programa LIBERNE. | | Technical report | | Conservation | |
| Palma L (1996) O lince ibérico (Lynx pardinus) no Algarve e Sudoeste do Alentejo. *Ciência e Natureza* 2: 7–14. | | non-SCI Journals | | Population Status | |
| Palma L, Beja P, Rodrigues M (1999) The use of sighting data to analyse Iberian lynx habitat and distribution. Journal of Applied Ecology 36: 812–824. | | SCI Journals | | General ecology | |
| Paula J, Santos J, Monteiro B, Marques T, Novais R, Costa H, Mascarenhas M (2011) Distribución y dieta de la nutria en el área de influencia del Aprovechamiento Hidroeléctrico del Alvito. In: *Resúmenes X Congreso de la SECEM*, Fuengirola, Málaga, Spain. | | Conference Proceedings | | General ecology | |
| Paupério J, Monterroso P, Rebelo H, Moreira P, Castro D, Silva A, Alves PC (2008) Avaliação do Estado Actual do Conhecimento sobre os Mamíferos do Parque Natural do Douro Internacional. Research Center in Biodiversity and Genetic Resources/Instituto de Conservação da Natureza e da Biodiversidade, Vairão, Portugal. | | Technical report | | Population Status | |
| Pedrosa M (2000) *Aspectos da ecologia da lontra (Lutra lutra) em ambientes aquáticos de Vilamoura (Sul de Portugal).* Final Degree thesis, Faculty of Sciences of the University of Lisbon Lisbon, Portugal. | | Thesis | | General ecology | |
| Pedroso NM (1997) A lontra (Lutra lutra Linnaeus, 1758) na barragem da Aguieira. Final Degree thesis, Faculty of Sciences of the University of Lisbon Lisbon, Portugal. | | Thesis | | Population Status | |
| Pedroso NM (2003) *Implicações ambientais do planeamento e gestão de grandes barragens: o caso da lontra*. MSc thesis, Faculty of Sciences and Technology of Universidade Nova de Lisboa, Lisbon, Portugal. | | Thesis | | Conservation | |
| Pedroso NM (2012) *Otters and dams in mediterranean habitats: a conservation ecology approach.* PhD thesis, Faculty of Sciences of the University of Lisbon, Lisbon, Portugal. | | Thesis | | Conservation | |
| Pedroso NM, Chambel I, Matos H, Sales-Luís T, Sousa M, Santos-Reis M (1999) La influencia de la estacionalidad de los recursos sobre la dieta de la Nutria en diferentes hábitats de Portugal. In: Resúmenes IV Jornadas de la SECEM, Segovia, Spain. | | Conference Proceedings | | General ecology | |
| Pedroso NM, Marques TA, Santos-Reis M (2014) The response of otters to environmental changes imposed by the construction of large dams. *Aquatic Conservation: Marine and Freshwater Ecosystems* 24: 66–80. | | SCI Journals | | Conservation | |
| Pedroso NM, Sales-Luís T (1998) Use of extensive lentic system by the eurasian otter in central Portugal. In: Reig S (ed) Abstracts of the Euro-American Mammal Congress, Santiago Compostela, Spain. | | Conference Proceedings | | General ecology | |
| Pedroso NM, Sales-Luís T, Loureiro F, Santos-Reis M (2008) Assessing the impact of dam implementation on otters: the cases of Alqueva (SE Portugal) and Sabor (NE Portugal) dams. In: *European Otter Worksho*p. Moravske Toplice, Slovenia. | | Conference Proceedings | | Conservation | |
| Pedroso NM, Sales-Luís T, Santos-Reis M (2003) Uso de los grandes embalses en Portugal por la nutria. In: *Resúmenes VI Jornadas de la SECEM*, Ciudad Real, Spain. | | Conference Proceedings | | Conservation | |
| Pedroso NM, Sales-Luís T, Santos-Reis M (2007) Use of Aguieira Dam by Eurasian otters in Central Portugal. *Folia Zoologica* 56: 365–377. | | SCI Journals | | General ecology | |
| Pedroso NM, Sales-Luís T, Santos-Reis M (2011) Importancia de las monitorizaciones a largo plazo para evaluar la respuesta de especies a los impactos de embalses: la nutria en el embalse de Alqueva (Portugal) - implicaciones de gestión. In: *Resúmenes X Congreso de la SECEM*, Fuengirola, Málaga, Spain. | | Conference Proceedings | | Conservation | |
| Pedroso NM, Sales-Luís T, Santos-Reis M (2011) Long Term Monitoring of the Eurasian Otter in the Alqueva Dam. In: Prigioni C, Loy A, Balestrieri A, Remo (eds) *Proceedings of the XIth International Otter Colloquium*, 97. Associazone Teriologica Italiana, Pavia, Italy. | | Conference Proceedings | | Conservation | |
| Pedroso NM, Sales-Luís T, Santos-Reis M (2014) The Eurasian otter Lutra lutra (Linnaeus , 1758) in Portugal. In: Ruiz-González A , In: Rubines J , In: Luzuriaga JL de (eds) *Conservation and management of semi-aquatic mammals in southwestern Europe*, 133–144. Aranzadi Society of Sciences. | | Book | | Conservation | |
| Pedroso NM, Santos E, Loureiro F, Martins AR, Lecoq M, Hotham P (2009) El papel del programa Lince (LPN/FFI) en la recuperación del hábitat y de las presas del lince ibérico en el sur de Portugal. In: Resúmenes IX Jornadas de la SECEM, Bilbao, Spain. | | Conference Proceedings | | Conservation | |
| Pedroso NM, Santos-Reis M (2006) Summer diet of Eurasian Otters in large dams of South Portugal. *Hystrix* 17: 117–128. | | SCI Journals | | General ecology | |
| Pedroso NM, Santos-Reis M (2007) Response of otters to new habitat opportunities: the islands of Alqueva reservoir (SE Portugal). In: Hájková P, Rüziczová O (eds) *Abstracts of the 25th Mustelid Colloquium*, 38. Trebon, Czech Republic. | | Conference Proceedings | | General ecology | |
| Pedroso NM, Santos-Reis M (2009) Assessing Otter Presence in Dams: A Methodological Proposal. *IUCN Otter Specialist Group Bulletin* 26: 97–110. | | non-SCI Journals | | Others | |
| Pedroso NM, Santos-Reis M, Vasconcelos L (2004) O uso de grandes barragens pela lontra no Alentejo. Revista de Biologia: 211–224. | | non-SCI Journals | | Conservation | |
| Pedroso NM, Vasconcelos L, Santos-Reis M (2006) Large dams policy in Portugal: implications for conservation. In: Abstracts of the 1st European Congress on Conservation Biology, 147. Eger, Hungary. | | Conference Proceedings | | Conservation | |
| Pereira D (2003) *Predação de pequenos ruminantes domésticos por uma alcateia pertencente à população lupina a sul do rio Douro.* Final Degree thesis, Faculty of Sciences of the University of Lisbon Lisbon, Portugal. | | Thesis | | General ecology | |
| Pereira I, Ferreira JP, Grilo C, Sousa I, Santos-Reis M (2001) *Estudos de Biologia e Ecologia do gato-bravo Felis silvestris na área de regolfo do Alqueva e Pedrogão.* Centre for Environmental Biology, Faculty of Sciences of the University of Lisbon/Centro de Estudos da Avifauna Ibérica. | | Technical report | | General ecology | |
| Pereira I, Lourenço S, Márques T, Santos-Reis M (1999) Ecología espacio-temporal comparada de ginetas y garduñas en Serra de Grândola (SO Portugal). In: *Resúmenes IV Jornadas de la SECEM*, Segovia, Spain. | | Conference Proceedings | | General ecology | |
| Pereira I, Santos-Reis M (2004) Patterns of resting sites use by Stone martens and Genets inhabiting a cork oak woodland. In: *Abstracts of the 4th International Martes Symposium: Martes in Carnivore Communities*, 37. University of Lisbon, Lisbon, Portugal. | | Conference Proceedings | | General ecology | |
| Pereira Í. (1999). *Comportamento de selecção de locais de repouso diurno em Genetta genetta e Martes foina na Serra de Grândola.* Final Degree thesis, Faculty of Sciences of the University of Lisbon Lisbon, Portugal. | | Thesis | | General ecology | |
| Pereira JP, Pereira I, Marques JT, Grilo C, Baltazar C, Santos-Reis M (2005) Modelling wild cat (Felis silvestris) distribution in a Portuguese Natura 2000 Site (SPA Moura-Barrancos, SE Portugal). In: *Abstracts of the Symposium: Biology and Conservation of the European Wildcat (Felis silvestris silvestris)*, 15. | | Conference Proceedings | | Population Status | |
| Pereira M, Petrucci-Fonseca F, Magalhães CP (1985) Wolf ecology in Portugal. In: *Actes du Symposium Prédateurs*, 122–167. Lisbon, Portugal. | | Conference Proceedings | | General ecology | |
| Pereira MJ (1985) Effects of human and wolf (Canis lupus) presence on a roe deer (Capreolus capreolus) population in Northeastern Portugal (Serra da Nogueira). In: *Transactions of the XVII Congress of the International Union of Game Biologists*, 671–678. Brussels, Belgium. | | Conference Proceedings | | General ecology | |
| Pereira MR (1984) *Elementos sobre algumas espécies cinegéticas do concelho de Bragança. Dados obtidos em 1982.* | | non-SCI Journals | | Population Status | |
| Pereira P (2010) *Selecção de habitat por carnívoros na Serra do Bussaco.* MSc thesis, University of Aveiro, Aveiro, Portugal. | | Thesis | | General ecology | |
| Pereira P, Silva AA da, Alves J, Matos M, Fonseca C (2012) Coexistence of carnivores in a heterogeneous landscape: habitat selection and ecological niches. *Ecological Research* 27: 745–753. | | SCI Journals | | General ecology | |
| Petrucci-Fonseca F (1979) *Canis lupus signatus Cabrera, 1907. Estudo do seu impacto nos animais domésticos e na população de corços de Trás-os-Montes (NE Portugal).* Final Degree thesis, Faculty of Sciences of the University of Lisbon Lisbon, Portugal. | | Thesis | | General ecology | |
| Petrucci-Fonseca F (1981) The protection of the wolf in Portugal. In: *XV Congreso International de Fauna Cinegética y Silvestre*, 661–664. Trujillo, Spain. | | Conference Proceedings | | Conservation | |
| Petrucci-Fonseca F (1987) The Signatus Project - a strategy for wolf conservation in Portugal. In: Reed DW (ed) *Spirit of Enterprise - The 1987 Rolex Awards*, 387–389. Van Nostrand Reinhold, U.K. | | Book | | Conservation | |
| Petrucci-Fonseca F (1990) *O lobo (Canis lupus signatus Cabrera, 1907) em Portugal. Problemática da sua conservação.* PhD thesis, Faculty of Sciences of the University of Lisbon, Lisbon, Portugal. | | Thesis | | Conservation | |
| Petrucci-Fonseca F (1992) Population trends of wolf in Portugal. In: Promberger C, Schroder W (eds) *Wolves in Europe - status and perspectives. Proceedings of the Workshop “Wolves in Europe - current status and prospects”,* 110–112. Munich Wildlife Society, Oberammergau. | | Book | | Population Status | |
| Petrucci-Fonseca F (1997) Estratégia para a conservação do lobo em Portugal. In: *Resúmenes I Congreso Hispano-Luso. Situación y conservación de las poblaciones de lobo en la Península Ibérica*, 68. Soria, Spain. | | Conference Proceedings | | Conservation | |
| Petrucci-Fonseca F (1999) Wolves and livestock guarding dogs in Portugal: Partners in survival. In: *Abstracts of the Wolf & Co. 1999. International Symposium on Canids – Behavior and Conservation. A Challenges to Mankind’s Tolerance*, Cologne, Germany. | | Conference Proceedings | | Conservation | |
| Petrucci-Fonseca F (2000) The future of the Wolf in Portugal: Trends, Risk Factors and Conservation Challenges. In: *Proceedings and Agenda for Defenders of Wildlife’s Carnivores 2000: A Conference on Carnivore Conservation in the 21st Century*, Denver, Colorado, U.S.A. | | Conference Proceedings | | Conservation | |
| Petrucci-Fonseca F (2000) The recovery of livestock guarding dogs’ use and the iberian wolf conservation in Portugal - promising results. *Carnivore Damage Prevention News* 1: 8–9. | | non-SCI Journals | | Conservation | |
| Petrucci-Fonseca F (2000) Wolf conservation in Portugal and the new millennium: New directions after 25 years of research. In: *Proceedings from Beyond 2000: Realities of Global Wolf Restoration, The International Wolf Center*, Duluth, Minnesota, U.S.A. | | Conference Proceedings | | Conservation | |
| Petrucci-Fonseca F .(1991) Vanishing predators: the case of the Iberian wolf. In: *Abstracts of the I European Congress of Mammalogy*, 40. Lisbon, Portugal. | | Conference Proceedings | | Conservation | |
| Petrucci-Fonseca F, Alexandre AS, Álvares F, Bessa-Gomes C, Cândido AT, Carreira R, Ribeiro S (1997) *Conservação do lobo em Portugal*. Grupo LOBO/Instituto da Conservação da Natureza, Lisbon, Portugal. | | Technical report | | Conservation | |
| Petrucci-Fonseca F, Alexandre AS, Álvares F, Cândido AT, Carreira R, Bessa-Gomes C (1996) Monitorização da população de lobo Ibérico em Portugal. In: Abstracts of the I Congresso Ibérico de Biólogos/Ambiente, Associação Portuguesa de Biólogos, Lisboa, Portugal. | | Conference Proceedings | | Population Status | |
| Petrucci-Fonseca F, Almendra L, Clemente A, Collaço MT, Matos J, Pires AE, Ribeiro S, Simões F (1997) Contributo para a Minimização do Impacto Económico dos Predadores sobre os Animais Domésticos. In: *Abstracts of the I Encontro Regional do Norte - Ambiente, Desenvolvimento, Autarquias e Educação*, Associação Portuguesa de Biólogos, Braga, Portugal. | | Conference Proceedings | | Conservation | |
| Petrucci-Fonseca F, Álvares F (1997) Evolução histórica da distribuição e análise da situação actual do lobo na metade Sul de Portugal. In: *Transactions of the II Jornadas sobre el Lobo Mediterráneo*, Associação Conservacionista Rómulo y Remo, Sevilha, Espanha. | | Conference Proceedings | | Population Status | |
| Petrucci-Fonseca F, Bessa-Gomes C, Álvares F (1995) *Áreas prioritárias para a conservação do lobo em Portugal. Perspectiva global e bases para a gestão e conservação do lobo no Parque Nacional da Peneda-Gerês*. Fundação da Faculdade de Ciências da Universidade de Lisboa/Instituto da Conservação da Natureza, Lisbon, Portugal. | | Technical report | | Conservation | |
| Petrucci-Fonseca F, Collares-Pereira MJ, Bacellar F, Marques-Pereira N (1991) Some parasitological and bacteriological aspects of the Iberian wolf population in Portugal. In: *Abstracts of the I European Congress of Mammalogy*, 82. Lisbon, Portugal. | | Conference Proceedings | | Health status | |
| Petrucci-Fonseca F, Guerra AM, Ferrão da Costa G (2009) *Plano de Monitorização da população lupina no âmbito da construção da A24 e A7 no Sítio Natura 2000 Alvão/Marão*. Grupo Lobo/Centre for Environmental Biology, Lisbon, Portugal. | | Technical report | | Conservation | |
| Petrucci-Fonseca F, Pires AE, Ribeiro S, Almendra L, Clemente A, Collaço MT, Matos J, Simões F (2000) Cães de gado na conservação do lobo em Portugal. Galemys 12: 135–148. | | non-SCI Journals | | Conservation | |
| Petrucci-fonseca F, Ribeiro S, Pires AE, Cruz C, Almada M (2002) The Conservation of the Iberian Wolf in Portugal: The Use of New and Traditional Techniques for Damage Prevention on Livestock. In: *Abstracts of the* *Wolf & Co. 2002 - A Challenge to Mankind’s Tolerance, Behaviour and Conservation*, Cologne, Germany. | | Conference Proceedings | | Conservation | |
| Pimenta V (1998) *Estudo comparativo de duas alcateias no nordeste do distrito de Bragança. Utilização do espaço e do tempo e hábitos alimentares.* Final Degree thesis, Faculty of Sciences of the University of Lisbon Lisbon, Portugal. | | Thesis | | General ecology | |
| Pimenta V, Barroso I, Álvares F, Correia J, Ferrão da Costa G, Moreira L et al. (2005) Situação Populacional do Lobo em Portugal: resultados do Censo Nacional 2002/2003. Instituto da Conservação da Natureza/Gurpo Lobo, Lisbon, Portugal. | | Technical report | | Population Status | |
| Pimenta V, Barroso I, Costa C, Diamantino J, Dias A, Fernandes M, Marques S, Alves Pinto CM (2012) Avaliação do risco de predação do lobo sobre o gado em Portugal: uma abordagem preliminar. In: *Abstracts of the III Iberian Wolf Congress*, 72. Lugo, Spain. | | Conference Proceedings | | Conservation | |
| Pinheiro P, Costa M, Ascensão F, Grilo C, Matos H, Silva F, Reto D, Santos-Reis M (2009) Stone Martens and Highway Interactions: Home Range Configurations and Crossing Movements. In: *Abstracts of the 27th Mustelid Coloquium*, 37. Lisbon, Portugal. | | Conference Proceedings | | Conservation | |
| Pinho S, Silva S, Alcântara T (1997) Centro de recuperaçâo do lobo ibérico (Canis lupus signatus Cabrera, 1907). In: *Resúmenes I Congreso Hispano-Luso. Situación y conservación de las poblaciones de lobo en la Península Ibérica*, 19. Soria, Spain. | | Conference Proceedings | | Conservation | |
| Pinto B (1998) *Abundância e ecologia espacio-temporal da comunidade de carnívoros da Serra de Grândola.* Faculty of Sciences of the University of Lisbon, Lisbon, Portugal. | | Thesis | | General ecology | |
| Pinto B, Fernandes M (2001) *Abordagem preliminar à distribuição do gato-bravo em Portugal*. Unpublished report. Instituto da Conservação da Natureza. | | Technical report | | Population Status | |
| Pinto B, Santos MJ (2002) *Abundância e dieta de quatro espécies de carnívoros na Serra de Grândola*. Naturlink. http://naturlink.sapo.pt | | non-SCI Journals | | General ecology | |
| Pinto N, Fonseca C (2005) Análisis espacio-temporal de la dieta de la nutria (Lutra lutra L.) en la Reserva Natural de Paúl de Arzila (RNPA). In: *Resúmenes VII Jornadas de la SECEM*, Valencia, Spain. | | Conference Proceedings | | General ecology | |
| Pinto NML, Alves da Silva A, Fonseca C (2009) Influence of three non-indigenous species in Otter diet in a Portuguese wetland. In: *Abstracts of the 2nd European Congress of Conservation Biology*, 204. Czech University of Life Sciences, Faculty of Environmental Sciences, Prague, Czech Republic. | | Conference Proceedings | | General ecology | |
| Pinto S, Cadete D, Petrucci-Fonseca F (2012) Curral de lobos: um fojo de cabrita a sul do rio Douro em Portugal. In: *Abstracts of the III Iberian Wolf Congress*, 83. Lugo, Spain. | | Conference Proceedings | | Others | |
| Pires A (1996) *Contribuição para a distinção bioquímica dos dejectos de lobo e de cão.* Final Degree thesis, Faculty of Sciences of the University of Lisbon Lisbon, Portugal. | | Thesis | | Others | |
| Pires AE, Fernandes ML (2003) Last lynxes in Portugal? Molecular approaches in a pre-extinction scenario. *Conservation Genetics* 4: 525–532. | | SCI Journals | | Genetics | |
| Pires AE, Reis A, Crespo A, Petrucci-Fonseca F (1997) O uso da Cromatografia de Camada Fina (TLC) na distinção molecular dos dejectos de lobo e de cão. In: *Abstracts of the I Congresso Ibérico de Biólogos/Ambiente*, Associação Portuguesa de Biólogos, Lisbon, Portugal. | | Conference Proceedings | | Genetics | |
| Pires AE, Ribeiro S, Petrucci-Fonseca F (1998) The recovery of livestock guarding dog breeds: Analysis of polymorphic microsatellites. In: *Abstrats of the 8th International Congress of the International Council of Archaeozoology*, Victoria, Canada. | | Conference Proceedings | | Conservation | |
| Pires JP (2001) Ecología alimentaria del zorro (Vulpes vulpes Linnaeus, 1758) en el Parque Natural de la Sierra de la Estrella- Región Central de Portugal. In: *Resúmenes V Jornadas de la SECEM*, Vitoria, Spain. | | Conference Proceedings | | General ecology | |
| Pita R, Mira A, Moreira F, Morgado R, Beja P (2009) Influence of landscape characteristics on carnivore diversity and abundance in Mediterranean farmland. Agriculture, Ecosystems & Environment 132: 57–65. | | non-SCI Journals | | General ecology | |
| Primavera P (1998) A cura para o mau ar do lobo. *Boletim informativo do Grupo Lobo* 13: 6. | | non-SCI Journals | | Conservation | |
| Primavera P, Álvares F (2004) Os Fojos dos lobos: Testemunhos da caça histórica ao lobo. *Caça & Cães* 77: 38–42. | | non-SCI Journals | | Conservation | |
| Primavera P, Álvares F (2005) O lobo na tradição oral das comunidades rurais *A Hora do Lobo: Contos com lobos*, 1–3. Grupo Lobo, Lisbon, Portugal. | | Book | | Conservation | |
| Primavera P, Álvares F (2006) Testemunhos da ancestral relação entre o lobo e as comunidades rurais na Serra de Arga. *Naturlink*. | | non-SCI Journals | | Conservation | |
| Primavera P, Álvares F, Petrucci-Fonseca F (2003) *Recuperação e valorização dos Fojos do Lobo. Protocolo Parque Nacional da Peneda-Gerês/Grupo Lobo,* Lisbon, Portugal. | | Technical report | | Conservation | |
| Proulx G, Aubry K, Birks J, Buskirk S, Fortin C, Frost H et al. (2005) World distribution and status of the genus Martes in 2000. In: Harrison DJ, Fuller AK, Proulx G (eds) *Martens and Fishers (Martes) in Human-altered Environments*, 21–76. Springer Science & Business Media. | | Book | | Population Status | |
| Quaglietta L, Fonseca V, Mira A, Boitani L (2011) Social interactions of the Eurasian Otter (Lutra lutra) in a Mediterranean environment. In: Prigioni C, Loy A, Balestrieri A, Remonti L (eds) *Proceedings of the XIth International Otter Colloquium*, 60. Associazone Teriologica Italiana, Pavia, Italy. | | Conference Proceedings | | General ecology | |
| Quaglietta L, Fonseca VC, Hájková P, Mira A, Boitani L (2013) Fine-scale population genetic structure and short-range sex-biased dispersal in a solitary carnivore, Lutra lutra. *Journal of Mammalogy* 94: 561–571. | | SCI Journals | | Genetics | |
| Quaglietta L, Fonseca VC, Mira A, Boitani, Luigi B (2014) Sociospatial organization of a solitary carnivore, the Eurasian otter (Lutra lutra). *Journal of Mammalogy* 95: 140–150. | | SCI Journals | | General ecology | |
| Quaglietta L, Hájková P, Mira A, Boitani L (2015) Eurasian otter (Lutra lutra) density estimate based on radio tracking and other data sources. *Mammal Research* 60: 127–137. | | SCI Journals | | Population Status | |
| Quaglietta L, Martins BH, de Jongh A, Mira A, Boitani L (2012) A low-cost GPS GSM/GPRS telemetry system: performance in stationary field tests and preliminary data on wild otters (Lutra lutra). *PLoS ONE* 7: e29235. | | SCI Journals | | Others | |
| Quaglietta L, Mira A, Boitani L (2011) Home ranges, activity patterns and habitat selection of Mediterranean otters (Lutra lutra): the need to cope with highly variable environmental conditions. In: Prigioni C, Loy A, Balestrieri A, Remonti L (eds) *Proceedings of the XIth International Otter Colloquium*, 18. Associazone Teriologica Italiana, Pavia, Italy. | | Conference Proceedings | | General ecology | |
| Quaglietta L, Zina H, Mira A, Boitani L (2009) Assessing the Use of Spraints as Indicators of Space Use by Otters. In: *Abstracts of the 27th Mustelid Coloquium*, 38. Lisbon, Portugal. | | Conference Proceedings | | Others | |
| Quaresma S (2002) *Aspectos da situação populacional e hábitos alimentares do lobo-ibérico a sul do rio Douro.* Final Degree thesis, Faculty of Sciences of the University of Lisbon Lisbon, Portugal. | | Thesis | | Population Status | |
| Queirós AI (1989) *Regime alimentar de Genetta genetta e sua relação trófica no interior da comunidade de mamíferos.* Serviço Nacional de Parques, Reservas e Conservação da Natureza, Lisbon, Portugal. | | Book | | General ecology | |
| Radhouani H, Igrejas G, Carvalho C, Pinto L, Gonçalves A, Lopez M et al. (2011) Clonal lineages, antibiotic resistance and virulence factors in vancomycin-resistant enterococci isolated from fecal samples of red foxes (Vulpes vulpes). *Journal of Wildlife Diseases* 47: 769–773. | | SCI Journals | | Health status | |
| Radhouani H, Igrejas G, Gonçalves A, Estepa V, Sargo R, Torres C, Poeta P (2013) Molecular characterization of extended-spectrum-beta-lactamase-producing Escherichia coli isolates from red foxes in Portugal. *Archives of Microbiology* 195: 141–144. | | SCI Journals | | Health status | |
| Ramos L (2014) *Assessing hybridization between wildcat and domestic cat: the particular case of Iberian Peninsula and some insights into North Africa.* MSc thesis, Faculty of Sciences of the University of Porto, Porto, Portugal. | | Thesis | | Genetics | |
| Ramos L, Monterroso P, Godinho R, Alves PC (2013) Hibridación entre el gato montés y el gato doméstico en la Península Ibérica: desarrollo y optimización de marcadores moleculares. In: *Resúmenes XI Jornadas de la SECEM*, Avilés, Spain, (Oral). | | Conference Proceedings | | Genetics | |
| Rauel V (2002) *Biologie d’une population de loup: matériels et méthodes. Etude du regime alimentaire dans le Centre du Portugal.* Faculty of Sciences of the University of Angers, Angers, France. | | Thesis | | General ecology | |
| Rebelo P (2012) *Insights into the trophic ecology of the Pine Marten (Martes martes) in Northwestern Iberian Peninsula.* MSc thesis, Faculty of Sciences of the University of Porto, Porto, Portugal. | | Thesis | | General ecology | |
| Rei C (2001) *Contribuição para o estudo da ecologia de um grupo social de texugos (Meles meles, L.) na Serra de Grândola.* Final Degree thesis, Faculty of Sciences of the University of Lisbon Lisbon, Portugal. | | Thesis | | General ecology | |
| Requeijão V (2014) *Análise do comportamento predatório de lince-ibérico (LYNX PARDINUS) em cativeiro através de enrriquecimento ambiental.*  Master thesis, Faculty of Social Sciences and Humanities, Universidade Nova de Lisboa, Lisboa, Portugal. | | Thesis | | General ecology | |
| Reto D, Grilo C, Santos-Reis M (2009) How do mustelids respond to highways mitigation measures?. In: *Abstracts of the 27th Mustelid Coloquium*, 59. Lisbon, Portugal. | | Conference Proceedings | | Conservation | |
| Ribeiro Dias MI, Sargo R, Valente J, Raposo M, Rio-Maior H, Brandão R, Santos N, Álvares F, Silva F (2012) Treatment of a diaphyseal radial complex fracture associated to an ulnar simple fracture and recuperation of an Iberian wolf. In: *Abstracts of the III Iberian Wolf Congress*, 55. Lugo, Spain. | | Conference Proceedings | | Health status | |
| Ribeiro I (2015) *Aplicação de programas educativos: o caso de estudo do lobo-ibérico (Canis lupus signatus, 1907).* MSc thesis, Faculty of Sciences of the University of Lisbon, Lisbon, Portugal. | | Thesis | | Conservation | |
| Ribeiro S (1996) *A problemática dos cães vadios na conservação do lobo em Portugal: Estudo da situação dos cães vadios em Portugal e caracterização do comportamento predatório do cão e do lobo.* Final Degree thesis, Faculty of Sciences of the University of Lisbon Lisbon, Portugal. | | Thesis | | Conservation | |
| Ribeiro S, Almada M, Petrucci-Fonseca F (2003) Recovery of traditional techniques of livestock protection from wolf predation in Portugal. In: *Abstracts of the World Wolf Congress 2003: Bridging Science and Community*, 40. Banff, Alberta, Canada. | | Conference Proceedings | | Conservation | |
| Ribeiro S, Petrucci-Fonseca F (2004) Recovering the use of livestock guarding dogs in Portugal: results of a long-term action. *Carnivore Damage Prevention News* 7: 2–5. | | non-SCI Journals | | Conservation | |
| Ribeiro S, Petrucci-Fonseca F (2007) The use of Livestock Guarding Dogs in Portugal: Results from a long-term experience. In: *Proceedings of the International Symposium: Large Carnivores and Agriculture Comparing Experiences across Italy and Europe*, 18. Assisi, Italy. | | Conference Proceedings | | Conservation | |
| Ribeiro S, Petrucci-Fonseca F (2012) O Programa Cão de Gado: da tradição à conservação do lobo em Portugal. In: *Abstracts of the III Iberian Wolf Congress*, 65. Lugo, Spain. | | Conference Proceedings | | Conservation | |
| Ribeiro S, Sousa L, Petrucci-Fonseca F (2012) Os lobos têm personalidade? Uma primeira aproximação ao estudo das diferenças comportamentais em lobos. In: *Abstracts of the III Iberian Wolf Congress*, 48. Lugo, Spain. | | Conference Proceedings | | General ecology | |
| Rio-Maior H (2002) *Aspectos comportamentais do texugo (Meles meles, L.) na Serra de Grândola.* Final Degree thesis, Faculty of Sciences of the University of Lisbon Lisbon, Portugal. | | Thesis | | Others | |
| Rio-Maior H (2005) Eco-ethological relationships between the iberian wolf and free grazing livestock: implications for conservation in Northwest Portugal. In: *Abstracts of the Frontiers of Wolf Recovery Conference*, 82–83. Colorado Springs, U.S.A. | | Conference Proceedings | | Conservation | |
| Rio-Maior H, Brandão R, Sargo R, Valente J, Silva F, Álvares F (2012) Devolução à Natureza de dois lobos submetidos a intervenções cirúrgicas: uma perspetiva clínica e ecológica. In: *Abstracts of the III Iberian Wolf Congress*, 51. Lugo, Spain. | | Conference Proceedings | | Conservation | |
| Rio-Maior H, Godinho R, Álvares F (2009) Monitorização e Investigação do lobo no Alto Minho. *Tribuna da Natureza* 30: 4–7. | | non-SCI Journals | | Population Status | |
| Rio-Maior H, Godinho R, Álvares F (2009) *Projecto de Investigação e Conservação do lobo no Noroeste de Portugal – Ano II*. VERANDA/Research Center in Biodiversity and Genetic Resources. | | Technical report | | Conservation | |
| Rio-Maior H, Godinho R, Nakamura M, Álvares F (2012) Comportamento social e espacial de um núcleo de 5 alcateias no noroeste de Portugal. In: *Abstracts of the III Iberian Wolf Congress*, 29. Lugo, Spain. | | Conference Proceedings | | General ecology | |
| Rio-Maior H, Malveiro E, Álvares F, Petrucci-Fonseca F (2005) O lobo e o gado bovino e equino pastoreado em liberdade no Noroeste de Portugal: Um estudo das relações ecológicas. *Naturlink*. | | non-SCI Journals | | Conservation | |
| Rio-Maior H, Malveiro E, Álvares F, Petrucci-Fonseca F (2006) *O lobo e o gado extensivo no Noroeste de Portugal – Um estudo das relações ecológicas. Center for Environmental Biology, Faculty of Sciences of the University of Lisbon,* Lisbon, Portugal. | | Technical report | | General ecology | |
| Rio-Maior H, Nakamura M, Álvares F (2011) Evaluación del comportamiento del lobo frente a los parques eólicos. Un estudio en el Alto Miño (NO Portugal). In: *Resúmenes X Congreso de la SECEM*, 123. Fuengirola, Málaga, Spain. | | Conference Proceedings | | Conservation | |
| Rocha M, Silva CSS, Fonseca CICR, Henriques IMM, Oliveira PMM (1995) *Estudo integrado do biótopo do carvalhal da Mata de Albergaria - Relatório Intercalar*. Equipa Corço - Porto, Porto, Portugal. | | Technical report | | General ecology | |
| Rocha S, Petrucci-Fonseca F (1998) The impact of stray/feral dogs on livestock and game in Portugal. Implications for wolf conservation. In: Reig S (ed) *Abstracts of the Euro-American Mammal Congress*, 270. Santiago Compostela, Spain. | | Conference Proceedings | | Conservation | |
| Rodrigues D, Simões L, Mullins J, Goebel J, Mendes R, Moreira F et al. (2013) The American mink (Neovison vison) in Portugal: current status and ecological insights. In: *Abstracts of the* *Wild Mustelid Conference*, Oxford, U.K. | | Conference Proceedings | | General ecology | |
| Rodrigues D, Simões L, Mullins J, Lampa S, Mendes R, Fernandes C, Rebelo R, Santos-Reis M (2015) Tracking the expansion of the American mink (Neovison vison) range in NW Portugal. *Biological Invasions* 17: 13–22. | | SCI Journals | | Population Status | |
| Rodrigues M (1996) *A raposa nos Parques Naturais de Sintra-Cascais e do Alvão: estratégia de utilização e recursos.* Final Degree thesis, Faculty of Sciences of the University of Lisbon Lisbon, Portugal. | | Thesis | | General ecology | |
| Rodrigues M, Fernandes CA, Palomares F, Amorim, Isabem R, Bruford MW, Santos-Reis M (2009) Isolation and characterization of 11 tetranucleotide microsatellite loci in the Egyptian mongoose (Herpestes ichneumon). *Molecular Ecology Resources* 9: 1205–1208. | | SCI Journals | | Genetics | |
| Rodrigues M, Santos-Reis M, Elmeros M, Fernandes CA (2012) Microsatellite markers for genetic studies in the weasel (Mustela nivalis). *European Journal of Wildlife Research* 58: 507–510. | | SCI Journals | | Genetics | |
| Rodrigues S, Coelho JP, Bandeira V, Barros T, Duarte AC, Fonseca C, Pereira ME (2014) Mercury Bioaccumulation in the Egyptian Mongoose (Herpestes ichneumon): Geographical, Tissue, Gender and Age Differences. *Water, Air, & Soil Pollution* 225: 2005. | | SCI Journals | | Health status | |
| Rodriguez-Refojos C, Zuberogoitia I, Rosalino LM, Zabala J, Santos MJ, Santos-Reis M, Camps D (2011) Geographical and sexual differences in body size of common genets, Genetta genetta (Viverridae, Carnivora), in south-western Europe (Iberian Peninsula). *Folia Zoologica* 60: 54–62. | | SCI Journals | | Others | |
| Roque S (1999) *Estudo eto-ecológico do lobo-ibérico no Noroeste de Portugal.* Final Degree thesis, Faculty of Sciences of the University of Lisbon Lisbon, Portugal. | | Thesis | | General ecology | |
| Roque S, Álvares F, Petrucci-Fonseca F (2001) Utilización espacio-temporal y hábitos alimentarios de un grupo reproductor de lobos en el noroeste de Portugal. *Galemys* 13: 179–198. | | non-SCI Journals | | General ecology | |
| Roque S, Bernardo J, Godinho R, Petrucci-Fonseca F, Álvares F (2013) *Plano de Monitorização do Lobo a Sul do Rio Douro – Zona Este. Relatório Técnico, Ano I.* CIBIO-UP/Grupo Lobo, 85 pp+ Anexos. | | Technical report | | Conservation | |
| Roque S, Espírito-Santo C, Grilo C, Rio-Maior H, Petrucci-Fonseca F (2005) *A população lupina a sul do Rio Douro em Portugal: análise temporal, atitudes públicas e aperfeiçoamento dos corredores ecológicos*. Center for Environmental Biology, Faculty of Sciences of the University of Lisbon, Lisbon, Portugal. | | Technical report | | Conservation | |
| Roque S, Godinho R, Cadete D, Pinto S, Bernardo J, Petrucci-Fonseca F, Álvares F (2011) *Plano de Monitorização do Lobo Ibérico nas áreas dos Projectos Eólicos das Serras de Montemuro, Freita, Arada e Leomil*. Research Center in Biodiversity and Genetic Resources - University of Porto/Grupo Lobo. | | Technical report | | Population Status | |
| Roque S, Godinho R, Petrucci-Fonseca F, Brotas G, Álvares F (2011) El lobo (Canis lupus) al sur del Río Duero en Portugal: nuevos avances en el seguimiento y conservación de una población amenazada. In: *Resúmenes X Congreso de la SECEM*, 126. | | Conference Proceedings | | Conservation | |
| Roque S, Grilo C, Quaresma S, Oliveira H, Petrucci-Fonseca F (2003) Conservation problems and solutions for an isolated Iberian wolf population in Portugal. In: *Abstracts of the World Wolf Congress 2003: Bridging Science and Community*, 61. Banff, Alberta, Canada. | | Conference Proceedings | | Conservation | |
| Roque S, Palmegiani I, Petrucci-Fonseca F, Álvares F (2012) O custo da necrofagia: estratégias de uso do território por uma alcateia a sul do rio Douro em Portugal. In: *Abstracts of the III Iberian Wolf Congress*, 32. Lugo, Spain. | | Conference Proceedings | | General ecology | |
| Rosa RI (2004) *Caracterização da Fauna Mamalógica da Área de Paisagem Protegida da Serra do Açor.* Final Degree thesis, University of Aveiro, Aveiro, Portugal. | | Thesis | | Population Status | |
| Rosa S (2003) *Distribuição e avaliação da qualidade do habitat para o gato-bravo no troço médio do Vale do Guadiana. Proposta de medidas de gestão.* Faculty of Sciences of the University of Lisbon, Lisbon, Portugal. | | Thesis | | Conservation | |
| Rosalino LM (1995) *A geneta no Parque Natural de Sintra-Cascais: distribuição e utilização dos recursos disponíveis.* Faculty of Sciences of the University of Lisbon, Lisbon, Portugal. | | Thesis | | General ecology | |
| Rosalino LM (2004) *Environmental determinants of badger (Meles meles) density and sociality in Mediterranean woodlands.* PhD thesis, Faculty of Sciences of the University of Lisbon, Lisbon, Portugal. | | Thesis | | Population Status | |
| Rosalino LM (2005) Spatial and trophic ecology of Portuguese badgers. In: Macdonald DW, Randall D, Hurst C (eds) *The Second WildCRU Review: Another Ten Years of Conservation Research*, 225–226. Wildlife Conservation Research Unit. | | Book | | General ecology | |
| Rosalino LM (2008) Revisão dos parasitas e outros agentes infecciosos que afectam as populações ibéricas de um carnívoro social - o Texugo euroasiático (Meles meles, Linnaeus 1758). *Revista Lusófona de Ciência e Medicina Veterinária* 2: 9–16. | | non-SCI Journals | | Health status | |
| Rosalino LM, Basto MP, Sales-Luís T, Pedroso NM, Tavares L, Vilela CL, Oliveira M (2013) Bacterial diversity in faecal microbiota of badgers (Meles meles Linnaeus, 1758) in Portugal. In: Gupta VK, Verma AK (eds) Animal Diversity, Natural History and Conservation, 1–17. Daya Publishing House. | | Book | | Health status | |
| Rosalino LM, do Rosário J, Santos-Reis M (2009) The role of habitat patches on mammalian diversity in cork oak agroforestry systems. *Acta Oecologica* 35: 507–512. | | SCI Journals | | General ecology | |
| Rosalino LM, Loureiro F, Macdonald DW, Santos-Reis M (2003) Food digestibility of an Eurasian badger (Meles meles) with special reference to the Mediterranean region. *Acta Theriologica* 48: 283–288. | | SCI Journals | | General ecology | |
| Rosalino LM, Loureiro F, Macdonald DW, Santos-Reis M (2005) Dietary shifts of the badger (Meles meles) in Mediterranean woodlands: an opportunistic forager with seasonal specialisms. *Mammalian Biology* 70: 12–23. | | SCI Journals | | General ecology | |
| Rosalino LM, Loureiro F, MacDonald DW, Santos-Reis M (2005) Ecología del tejón (Meles meles) en un alcornocal del suroeste de Portugal. In: Virgos E, Revilla E, Mangas JG, Domingo-Roura X (eds) *Ecología y conservación del tejón en ecosistemas mediterráneos*, 103–117. Sociedad Española de Conservación y Estudio de Mamíferos, Madrid, Spain. | | Book | | General ecology | |
| Rosalino LM, Loureiro F, Santos-Reis M, Macdonald DW (2001) Badger setts and letrines in a cork oak woodland, SW Portugal. In: *Resúmenes V Jornadas de la SECEM*, Vitoria, Spain. | | Conference Proceedings | | General ecology | |
| Rosalino LM, Loureiro F, Santos-Reis M, Macdonald DW (2002) First data on the social and spatial structure of an Eurasian badger (Meles meles L., 1758) population in a cork oak woodland (SW Portugal). *Revista de Biologia*: 147–154. | | non-SCI Journals | | General ecology | |
| Rosalino LM, Loureiro F, Santos-Reis M, Macdonald DW (2003) Habitat use of an Eurasian badger population in a heterogeneous and disturbed landscape, SW Portugal. *Comptes Rendus Biologies* 326: 225. | | SCI Journals | | General ecology | |
| Rosalino LM, MacDonald DW, Santos-Reis M (2004) Spatial structure and land-cover use in a low-density Mediterranean population of Eurasian badgers. *Canadian Journal of Zoology* 82: 1493–1502. | | non-SCI Journals | | General ecology | |
| Rosalino LM, MacDonald DW, Santos-Reis M (2005) Activity rhythms, movements and patterns of sett use by badgers, Meles meles, in a Mediterranean woodland. *Mammalia* 69: 395–408. | | SCI Journals | | General ecology | |
| Rosalino LM, Macdonald DW, Santos-Reis M (2005) Resource dispersion and badger population density in Mediterranean woodlands: is food, water or geology the limiting factor? *Oikos* 110: 441–452. | | SCI Journals | | General ecology | |
| Rosalino LM, Marques C, Santos-Reis M (2007) Highly specialist or just an efficient opportunism: otter predation in a trout fish farm of central-east Portugal. In: Hajkova P, Rüziczová O (eds) *Abstracts of the 25th Mustelid Colloquium*, Trebon, Czech Republic. | | Conference Proceedings | | General ecology | |
| Rosalino LM, Rodrigues M, Santos-Silva M, Santos-Reis M (2007) Unusual findings on host-tick interactions through carnivore scat analysis. *Experimental & applied acarology* 43: 293–302. | | SCI Journals | | Health status | |
| Rosalino LM, Rosa S, Santos-Reis M (2010) The Role of Carnivores as Mediterranean Seed Dispersers. *Annales Zoologici Fennici* 47: 195–205. | | SCI Journals | | General ecology | |
| Rosalino LM, Santos MJ, Beier P, Santos-Reis M (2008) Eurasian badger habitat selection in Mediterranean environments: Does scale really matter? *Mammalian Biology* 73: 189–198. | | SCI Journals | | General ecology | |
| Rosalino LM, Santos MJ, Domingos S, Rodrigues M, Santos-Reis M (2005) Estrutura populacional e dimensões corporais de carnívoros simpátricos num ecossistema Mediterrânico do Sudoeste Português. *Revista Biologia* 23: 135– 146. | | non-SCI Journals | | Population Status | |
| Rosalino LM, Santos MJ, Domingos S, Rodrigues M, Santos-Reis M (2005) Population structure and body size of sympatric carnivores in a Mediterranean landscape of SW Portugal. *Revista de Biologia*: 135–146. | | non-SCI Journals | | General ecology | |
| Rosalino LM, Santos MJ, Pereira I, Santos-Reis M (2009) Sex-driven differences in Egyptian mongoose’s (Herpestes ichneumon) diet in its northwestern European range. *European Journal of Wildlife Research* 55: 293–299. | | SCI Journals | | General ecology | |
| Rosalino LM, Santos MJ, Santos-Reis M (2007) Expansión geográfica del meloncillo en la Península Ibérica: ¿mapa de camino a la invasión? In: *Resúmenes VIII Jornadas de la SECEM*, Huelva, Spain. | | Conference Proceedings | | Population Status | |
| Rosalino LM, Santos MJ, Santos-Reis M (2013) Landscape and species interaction effects on mesocarnivore use of Mediterranean environments. In: *Abstracts of The 11th International Mammalogical Congress 2013*, Belfast, Northern Ireland. | | Conference Proceedings | | General ecology | |
| Rosalino LM, Santos-Reis M (1998) Selection of latrine sites by the common genet (Genetta genetta L., 1758). In: Reig S (ed) *Abstracts of the Euro-American Mammal Congress*, Santiago Compostela, Spain. | | Conference Proceedings | | General ecology | |
| Rosalino LM, Santos-Reis M (2002) Feeding habits of the common genet Genetta genetta (Carnivora: Viverridae) in a semi-natural landscape of central Portugal. *Mammalia* 656: 195–205. | | SCI Journals | | General ecology | |
| Rosalino LM, Santos-Reis M (2009) Fruit consumption by carnivores in Mediterranean Europe. *Mammal Review* 39: 67–78. | | *SCI Journals* | | General ecology | |
| Rosalino LM, Santos-Reis M (2010) Fruits and meso-carnivores in mediterranean Europe. In: *Abstracts of the 10th International Mammalogical Congress*, 109. Mendoza, Argentina. | | Conference Proceedings | | General ecology | |
| Rosalino LM, Sousa M, Pedroso NM, Basto MP, Rosário J, Santos MJ, Loureiro F (2010) The Influence of Food Resources on Red Fox Local Distribution in a Mountain Area of the Western Mediterranean. Vie et milieu - Life and Environment 60: 1–7. | | non-SCI Journals | | General ecology | |
| Rosalino LM, Torres J, Santos-Reis M (2006) A survey of helminth infection in Eurasian badgers (Meles meles) in relation to their foraging behaviour in a Mediterranean environment in southwest Portugal. *European Journal of Wildlife Research* 52: 202–206. | | SCI Journals | | Health status | |
| Rosário LP, Palma L, Ramos JG (1982) Perspectivas da conservação da fauna e atividade venatória no Algarve. In: *Abstracts of the* *II Congresso Nacional sobre o Algarve.* | | Conference Proceedings | | Conservation | |
| Ruiz-Olmo J, Jiménez J (1998) Revisión bibliográfica sobre los carnívoros semiacuáticos (Nutria y visones) en España y Portugal. In: Ruiz-Olmo J, Delibes M (eds) *La nutria en España ante el horizonte del año 2000*, 257–273. Sociedad Española para la Conservación y el Estudio de los Mamíferos, Madrid, Spain. | | Book | | Others | |
| Ruíz-Olmo J, Lafontaine L, Prigioni C, López-Martín JM, Santos-Silva M (2000) Pollution and its effects on Otter populations in South-Western Europe. In: *Proceedings of the First Otter Toxicology Conference*, 63–82. Isle of Skye, Scotland. | | Conference Proceedings | | Conservation | |
| Sales-Luís T (2008) Scale and season does matter: implications for otter conservation. In: *Proceedings of the Xth International Otter Colloquium*, Hwacheon, Korea. | | Conference Proceedings | | Conservation | |
| Sales-Luís T (2011) *Patterns of otter (Lutra Lutra) distribution and man-otter conflicts in river Sado basin: conservation implications.* PhD thesis, Faculty of Sciences of the University of Lisbon, Lisbon, Portugal. | | Thesis | | Conservation | |
| Sales-Luís T, Bissonette JA, Santos-Reis M (2012) Conservation of Mediterranean otters: the influence of map scale resolution. Biodiversity and Conservation 21: 2061–2073. | | SCI Journals | | Conservation | |
| Sales-Luís T, Freitas D, Antunes P, Santos-Reis M (2006) Eurasian otters (Lutra lutra) in southern Portugal: well for how long? In: *Abstracts of the 1st European Congress on Conservation Biology*, 153. Eger, Hungary. | | Conference Proceedings | | Conservation | |
| Sales-Luís T, Freitas D, Rosalino LM, Marques C, Santos-Reis M (2011) Fish farms and otter predation: the role of environment and management options. In: Andrews GL, Vexton LA (eds) *Fish Farms: Management, Disease Control and the Environment*, 133–159. Nova Science Publishers. | | Book | | General ecology | |
| Sales-Luís T, Freitas D, Santos-Reis M (2009) Key landscape factors for Eurasian otter Lutra lutra visiting rates and fish loss in estuarine fish farm. *European Journal of Wildlife Research* 55: 345–355. | | SCI Journals | | Conservation | |
| Sales-Luís T, Pedroso NM, Grilo C, Santos-Reis M (2001) Monitoring of Lutra lutra in the Predicted Inundation Area of the Largest Dam in Europe (Alqueva, SE Portugal). In: *Proceedings of the VIIIth International Otter Colloquium*, Valdivia, Chile. | | Conference Proceedings | | Population Status | |
| Sales-Luís T, Pedroso NM, Santos-Reis M (1998) Comparative analysis of the diet of the eurasian otter in a hydroelectric dam and associated tributaries in Portugal. In: Reig S (ed) Abstracts of the Euro-American Mammal Congress, Santiago Compostela, Spain. | | Conference Proceedings | | General ecology | |
| Sales-Luís T, Pedroso NM, Santos-Reis M (2007) Prey availability and diet of the Eurasian otter (Lutra lutra) on a large reservoir and associated tributaries. *Canadian Journal of Zoology* 85: 1125–1135. | | SCI Journals | | General ecology | |
| Santos E (2001) *Contribuição para o conhecimento da helmintofauna do Lobo-ibérico no Noroeste de Portugal, mediante técnicas coprológicas.* Final Degree thesis, Faculty of Sciences of the University of Lisbon Lisbon, Portugal. | | Thesis | | Health status | |
| Santos E, Lecoq M, Allcorn R, Emauz A, Loureiro F (2007) Lynx Programme: an Integrated Approach to Iberian Lynx Conservation in Southern Portugal. In: Hughes J, Mercer R (eds) *Abstracts of the Felid Biology and Conservation Conference*, 104. Oxford University, Oxford, U.K. | | Conference Proceedings | | Conservation | |
| Santos E, Loureiro F, Martins AR, Lecoq M (2009) Iberian Lynx habitat conservation on a traditional humanized landscape. In: *Abstracts of the 2nd European Congress of Conservation Biology*, 256. Czech University of Life Sciences, Faculty of Environmental Sciences, Prague, Czech Republic. | | Conference Proceedings | | Conservation | |
| Santos M, Matos H, Bissonette JA, Santos-Reis M (2006) Riparian areas in the fragmented landscape of southern Portugal: applications for conservation planning. In: Abstracts of the Society for Conservation Biology Annual Meeting, 152. San Jose, USA. | | Conference Proceedings | | Conservation | |
| Santos M, Vaz C, Travassos P, Cabral JA (2007) Simulating the impact of socio-economic trends on threatened Iberian wolf populations Canis lupus signatus in north-eastern Portugal. *Ecological Indicators* 7: 649–664. | | SCI Journals | | Conservation | |
| Santos MJ (1998) *Interacções espaciais e tróficas da comunidade de carnívoros da Serra de Grândola.* Faculty of Sciences of the University of Lisbon, Lisbon, Portugal. | | Thesis | | General ecology | |
| Santos MJ (2003) *Habitat selection by European badgers at multiple spatial scales: implications for the conservation of the montado.* MSc, Northern Arizona University, Arizona, USA. | | Thesis | | General ecology | |
| Santos MJ, Beier P (2008) Habitat selection by European badgers at multiple spatial scales in Portuguese Mediterranean ecosystems. *Wildlife Research* 35: 835–843. | | SCI Journals | | General ecology | |
| Santos MJ, Matos HM, Baltazar C, Grilo C, Santos-Reis M (2009) Is polecat (Mustela putorius) diet affected by “mediterraneity”? *Mammalian Biology* 74: 446–453. | | SCI Journals | | General ecology | |
| Santos MJ, Matos HM, Palomares F, Santos-Reis M (2011) Factors affecting mammalian carnivore use of riparian ecosystems in Mediterranean climates. *Journal of Mammalogy* 92: 1060–1069. | | SCI Journals | | General ecology | |
| Santos MJ, Pedroso NM, Ferreira JP, Matos HM, Sales-Luís T, Pereira Í et al. (2008) Assessing dam implementation impact on threatened carnivores: the case of Alqueva in SE Portugal. *Environmental Monitoring Assessment* 142: 47–64. | | non-SCI Journals | | Conservation | |
| Santos MJ, Pinto B, Santos-Reis M (2007) Trophic niche partitioning between two native and two exotic carnivores in SW Portugal. *Web Ecology* 7: 53–62. | | SCI Journals | | General ecology | |
| Santos MJ, Rosalino LM, Santos-Reis M (2006) Habitat Selection of Expanding Exotic Species: the Egyptian Mongoose in Portugal. In: Delach A (ed) *Abstracts of the Defenders of Wildlife’s Carnivores 2006 - Habitat, Challenges and Opportunities*, 41. St. Petersburg, Florida, U.S.A. | | Conference Proceedings | | General ecology | |
| Santos MJ, Santos-Reis M (2009) Stone marten (Martes foina) habitat in a Mediterranean ecosystem: integrating selection from multiple scales. In: *5th International Martes Symposium - Biology and Conservation of Martens, Sables, and Fishers: a New Synthesis*, 15. University of Washington, Seattle, USA. | | Conference Proceedings | | General ecology | |
| Santos MJ, Santos-Reis M (2010) Stone marten (Martes foina) habitat in a Mediterranean ecosystem: effects of scale, sex, and interspecific interactions. *European Journal of Wildlife Research* 56: 275–286. | | SCI Journals | | General Ecology | |
| Santos ML, Lopez MC (1997) Predaçâo de lobo ibérico (Canis lupus signatus Cabrera, 1907) sobre gado doméstico no Maciço da Gralheira, entre 1992-1997. In: *Resúmenes I Congreso Hispano-Luso. Situación y conservación de las poblaciones de lobo en la Península Ibérica*, 59. Soria, Spain. | | Conference Proceedings | | Conservation | |
| Santos N, Almendra C, Tavares L (2009) Serologic survey for canine distemper virus and canine parvovirus in free-ranging wild carnivores from Portugal. *Journal of Wildlife Diseases* 45: 221–226. | | SCI Journals | | Health status | |
| Santos N, Carvalho H, Rio-Maior H, Nakamura M, Álvares F (2012) Padrões espacio-temporais da sarna sarcóptica em canídeos selvagens e domésticos no Parque Nacional da Peneda-Gerês. In: *Abstracts of the III Iberian Wolf Congress*, 22. Lugo, Spain. | | Conference Proceedings | | Health status | |
| Santos N, Maior HR, Nakamura M, Roque S, Brandão R, Petrucci-Fonseca F et al. (2014) Hematology and serum biochemistry values of free-ranging Iberian wolves (Canis lupus) trapped by leg-hold snares. *European Journal of Wildlife Research* 61: 135–141. | | SCI Journals | | Health status | |
| Santos T, Fonseca C, Barros T, Godinho R, Bastos-Silveira C, Bandeira V, Rocha RG (2015) Using stomach contents for diet analysis of carnivores through DNA barcoding. *Wildlife Biology in Practice* 11: 31–39. | | non-SCI Journals | | General Ecology | |
| Santos-Reis M (1983) Present situation and conservancy of the river otter (Lutra lutra L., 1758) in Portugal. In: *3rd International Otter Symposium*, 24. Strasbourg, France. | | Conference Proceedings | | Conservation | |
| Santos-Reis M (1983) Status and distribution of the Portuguese Mustelids. *Acta Zoologica Fennica* 174: 213–216. | | non-SCI Journals | | Population Status | |
| Santos-Reis M (1985) Mustela erminea Linnaeus, 1758: A new mustelid to Portugal. *Mammalia* 49: 136–138. | | SCI Journals | | Population Status | |
| Santos-Reis M (1986) Os mustelídeos: símbolos de uma fauna ameaçada. *Quercus* 5: 14. | | non-SCI Journals | | Conservation | |
| Santos-Reis M (1986) Só em Portugal e Grécia se encontram populações de lontra viáveis de distribuição generalizada. *Quercus* 5: 15–16. | | non-SCI Journals | | Population Status | |
| Santos-Reis M (1989) *As doninhas ibéricas (Carnivora: Mustela). Um estudo taxonómico e ecológico.* PhD thesis, Faculty of Sciences of the University of Lisbon, Lisbon, Portugal. | | Thesis | | General ecology | |
| Santos-Reis M (1994) Doninha. *Forum Ambiente* 1: 71–72. | | non-SCI Journals | | Population Status | |
| Santos-Reis M (1995) Lobo. *Forum Ambiente* 19: 77–78. | | non-SCI Journals | | Population Status | |
| Santos-Reis M (1995) Lontra. *Forum Ambiente* 10: 73–74. | | non-SCI Journals | | Population Status | |
| Santos-Reis M (1996) Raposa. *Forum Ambiente* 24: 75–76. | | non-SCI Journals | | Population Status | |
| Santos-Reis M (1998) Sacarrabos. *Forum Ambiente* 48: 125–126. | | non-SCI Journals | | Population Status | |
| Santos-Reis M (2008) Mustelid ecology and conservation in Portugal: an overview and new challenges. In: Ronkay MT (ed) *Abstracts of the 26th Mustelid Colloquium*, 53. Budapest, Hungary. | | Conference Proceedings | | General ecology | |
| Santos-Reis M, Grilo C, Ascensão F, Mateus ARA, Serronha AM (2008) Road mortality and permeability for otters in a Mediterranean region. In: *2008 European Otter Workshop*, Slovenia. | | Conference Proceedings | | Conservation | |
| Santos-Reis M, Mathias M (1996) The historical and recent distribution and status of mammals in Portugal. *Hystrix* 8: 75–89. | | SCI Journals | | Population Status | |
| Santos-Reis M, Mira A, Basto MP, Pedroso NM (2011) Use of small and medium-sized water reservoirs by otters in a Mediterranean ecosystem. Animal Biology 61: 75–94. | | SCI Journals | | General ecology | |
| Santos-Reis M, Pedroso NM, Sales-Luís T (1997) Impacto das barragens na distribuição e ecologia da lontra em Portugal. A barragem da Aguieira, estudo de um caso. In: Resúmenes III Jornadas de la SECEM, Castelló d’Empúries, Gerona, Spain. | | Conference Proceedings | | Conservation | |
| Santos-Reis M, Petrucci-Fonseca F (1999) Carnívoros. In: Mathias ML (ed) Mamíferos terrestres de Portugal Continental, Madeira e Açores, 135–165. Instituto de Conservação da Natureza / Centro de Biologia Ambiental, Lisbon. | | Book | | Population Status | |
| Santos-Reis M, Rosalino LM, Loureiro F, Macdonald DW (2005) Los tejones en Portugal: distribución, estatus y conservación. In: Virgós E, Mangas JG, REvilla E, Roura XD (eds) *Ecología, distribución y estatus de conservación del tejón Ibérico*, 241–250. Sociedad Española de Conservación y Estudio de Mamíferos, Madrid, Spain. | | Book | | Population Status | |
| Santos-Reis M, Rosalino LM, Rodrigues M (1999) Lagomorfos, carnívoros e artiodáctilos (Mamíferos). In: Santos-Reis M, Correia AI (eds) Caracterização da flora e da fauna do montado da Herdade da Ribeira Abaixo (Grândola-Baixo Alentejo), 249–262. Centro de Biologia Ambiental, Lisbon. | | Book | | Population Status | |
| Santos-Reis M, Sales-Luís T, Freitas D, Marques C, Rosalino LM (2005) Fish farming and otters in Portugal: is there a conflict of interests? In: *Abstracts of the European Otter Workshop*, 40. Padula, Salermo, Italy. | | Conference Proceedings | | Conservation | |
| Santos-Reis M, Santos MJ, Lourenço S, Marques J, Pereira Í, Pinto B (2005) Relationships between stone martens, genets and cork oak woodlands in Portugal. In: Harrison DJ, Fuller AK, Proulx G (eds) *Martens and Fishers (Martes) in Human-altered Environments*, 147–172. Springer Science & Business Media. | | Book | | General Ecology | |
| Santos-Reis M, Trindade A, Beja P (1995) Situation et état des recherches sur la loutre au Portugal. Cahiers d’Ethologie 15: 181–194. | | SCI Journals | | Others | |
| Saraiva MJ, Salvador ÂC, Fernandes T, Ferreira JP, Barros AS, Rocha SM, Fonseca C (2014) Three mammal species distinction through the analysis of scats chemical composition provided by comprehensive two-dimensional gas chromatography. *Biochemical Systematics and Ecology* 55: 46–52. | | SCI Journals | | Others | |
| Sargo R, Loureiro F, Catarino AL, Valente J, Silva F, Cardoso L, Otranto D, Maia C (2014) First Report of Thelazia Callipaeda in Red Foxes (Vulpes vulpes) from Portugal. *Journal of Zoo and Wildlife Medicine* 45: 458–460. | | SCI Journals | | Health status | |
| Sarmento P (1995) La situación actual del lince ibérico (Lynx pardinus, Temmick 1824) en la Sierra de Malcata (Portugal). In: *Resúmenes II Jornadas de la SECEM*, Soria, Spain. | | Conference Proceedings | | Population Status | |
| Sarmento P (1996) Feeding ecology of the European wildcat Felis silvestris in Portugal. *Acta Theriologica* 41: 409–414. | | SCI Journals | | General Ecology | |
| Sarmento P (2002) Propousal of the action plan for the conservation of the Iberian lynx in Portugal. In: *Abstracts of the International Seminar on the Iberian Lynx*, 10. Andujar, Spain. | | Conference Proceedings | | Conservation | |
| Sarmento P (2002) Status survey of Iberian Lynx in Portugal. In: *Abstracts of the International Seminar on the Iberian Lynx*, 9. Andujar, Spain. | | Conference Proceedings | | Population Status | |
| Sarmento P (2004) Iberian Lynx in Portugal: Status and Conservation efforts since 2002. In: Simón MA, Cadenas R, Leiva A (eds) *Proceedings of the II Seminario Internacional sobre la conservación del Lince Ibérico*, 89–90. Consejaria de Medio Ambiente, Junta de Andalucia, Córdoba, Portugal. | | Conference Proceedings | | Population Status | |
| Sarmento P (2010) *Habitat-species interactions in a carnivore community.* PhD thesis, Universidade do Averio, Aveiro, Portugal. | | Thesis | | General ecology | |
| Sarmento P, Cruz J (1995) Ecología trófica del gato montés (Felis silvestris Schreber, 1777) en un hábitat mediterráneo. In: *Resúmenes III Jornadas de la SECEM*, Castelló d’Empúries, Gerona, Spain. | | Conference Proceedings | | General ecology | |
| Sarmento P, Cruz J (1998) *Ecologia e conservação do lince-ibérico e da comunidade de carnívoros da Serra da Malcata*. Instituto da Conservação da Natureza. | | Technical report | | Conservation | |
| Sarmento P, Cruz J, Eira C, Fonseca C (2009) Evaluation of camera trapping for estimating red fox abundance. *Journal of Wildlife Management* 73: 1207–1212. | | SCI Journals | | General ecology | |
| Sarmento P, Cruz J, Eira C, Fonseca C (2009) Spatial colonization by feral domestic cats Felis catus of former wildcat Felis silvestris silvestris home ranges. *Acta Theriologica* 54: 31–38. | | SCI Journals | | General ecology | |
| Sarmento P, Cruz J, Eira C, Fonseca C (2011) Modeling the occupancy of sympatric carnivorans in a Mediterranean ecosystem. *European Journal of Wildlife Research* 57: 119–131. | | SCI Journals | | General ecology | |
| Sarmento P, Cruz J, Eira C, Fonseca C (2014) A spatially explicit approach for estimating space use and density of common genets. *Animal Biodiversity and Conservation* 37: 23–33. | | SCI Journals | | General ecology | |
| Sarmento P, Cruz J, Ferreira C, Monterroso P, Serra R, Tarroso P, Negrões N (2009) Conservation status and Action Plan for the recovery of Iberian lynx populations in Portugal. In: Vargas A, Breitenmoser C, Breitenmoser U (eds) *Iberian Lynx Ex situ Conservation: An Interdisciplinary Approach*, 32–41. Fundación Biodiversidad / IUCN Cat Specialist Group. | | Book | | Conservation | |
| Sarmento P, Cruz J, Monterroso P, Tarroso P, Ferreira C, Negrões N (2004) *The Iberian lynx in Portugal. Status survey and conservation action plan*. Instituto da Conservação da Natureza. | | Technical report | | Conservation | |
| Sarmento P, Cruz J, Monterroso P, Tarroso P, Ferreira C, Negrões N (2005) El lince ibérico en Portugal. In: Guzmán López-Ocón JN, García González FJ, Garrote Alonso G, Pérez De Ayala R, Iglesias Llamas MC (eds) *El lince ibérico (Lynx pardinus) en España y Portugal. Censo-diagnóstico de sus poblaciones*, 174. Dirección General para la Biodiversidad, Madrid, Spain. | | Book | | Conservation | |
| Sarmento P, Cruz J, Monterroso P, Tarroso P, Ferreira C, Negrões N (2005) Iberian lynx conservation in Portugal: Dilemmas and solutions. *Wildlife Biology* in Practice 1: 156–162. | | non-SCI Journals | | Conservation | |
| Sarmento P, Cruz J, Monterroso P, Tarroso P, Ferreira C, Negrões N, Eira C (2009) Status survey of the critically endangered Iberian lynx Lynx pardinus in Portugal. *European Journal of Wildlife Research* 55: 247–253. | | SCI Journals | | Population Status | |
| Sarmento P, Cruz J, Paula A, Eira C, Capinha M, Ambrósio I, Ferreira C, Fonseca C (2012) Occupancy, colonization and extinction patterns of rabbit populations: implications for Iberian lynx conservation. *European Journal of Wildlife Research* 58: 523–533. | | SCI Journals | | General ecology | |
| Sarmento P, Cruz J, Tarroso P, Fonseca C (2006) Space and habitat selection by female European wild cats (Felis silvestris silvestris). *Wildlife Biology in Practice* 2: 79–89. | | non-SCI Journals | | General ecology | |
| Sarmento P, Cruz J, Tarroso P, Gonçalves P, Vingada J V. (2001) Restoration of habitat and preys for the conservation of the Iberian lynx (Lynx pardinus) in Serra da Malcata (Portugal). In: *Resúmenes V Jornadas de la SECEM*, Vitoria, Spain. | | Conference Proceedings | | Conservation | |
| Sarmento P, Cruz JP, Eira C, Fonseca C (2009) Habitat selection and abundance of common genets Genetta genetta using camera capture-mark-recapture data. *European Journal of Wildlife Research* 56: 59–66. | | SCI Journals | | General ecology | |
| Seabra AF (1910) Catalogue systématique des vertébrés du Portugal. I. Mammifères. II. Oiseaux. *Bulletin de la Societé Portugaise des Sciences Naturelles* 4: 91–115. | | non-SCI Journals | | Population Status | |
| Seara M, Costa G, Roque S, Rio-Maior H, Álvares F, Petrucci-Fonseca F, Grilo C (2012) Predicting occurrence of Iberian wolf: the role of sample size and spatial scale. In: *Abstracts of the III Iberian Wolf Congress*, 16. Lugo, Spain. | | Conference Proceedings | | Others | |
| Segovia JM, Torres J, Miquel J (2002) The red fox, Vulpes vulpes L., as a potential reservoir of zoonotic flukes in the Iberian Peninsula. *Acta Parasitologica* 47: 163–166. | | SCI Journals | | Health status | |
| Semedo-Lemsaddek T, Nóbrega CS, Ribeiro T, Pedroso NM, Sales-Luís T, Lemsaddek A et al. (2013) Virulence traits and antibiotic resistance among enterococci isolated from Eurasian otter (Lutra lutra). *Veterinary Microbiology* 163: 378–382. | | SCI Journals | | Health status | |
| Serronha A, Mateus ARA, Eaton F, Santos-Reis M, Grilo C (2012) Towards effective culvert design: monitoring seasonal use and behavior by Mediterranean mesocarnivores. *Environmental Monitoring Assessment* 185: 6235–46. | | non-SCI Journals | | Conservation | |
| Sierra P, Álvares F (2005) *Monitorização do lobo (Canis lupus) na área de influência do Parque Nacional da Peneda-Gerês*. Grupo Lobo/PNPG. | | Technical report | | Population Status | |
| Silva A (2009) *Monitoring of Iberian wolf expansion in Sabugal: Malcata Region.* Final Degree thesis, Faculty of Sciences of the University of Lisbon, Lisbon, Portugal. | | Thesis | | Population Status | |
| Silva A (2014) *Avaliação estomatológico-dentária da cavidade oral na raposa vermelha (Vulpes vulpes).* MSc thesis, University of Trás-os-Montes and Alto Douro, Vila Real, Portugal. | | Thesis | | Health status | |
| Silva A, Valente J, Viegas C (2012) Lesões periodontais em achados de necrópsia de três raposas vermelhas (Vulpes vulpes). In: *Abstracts of the IV Congresso da Fauna Selvagem WAVES Portugal*, 64–65. Bragança, Portugal. | | Conference Proceedings | | Others | |
| Silva C (1999) *Predação por lontra, Lutra lutra L., em habitats estivais de uma ribeira intermitente mediterrânica.* Final Degree thesis, University of Algarve, Faro, Portugal. | | Thesis | | General ecology | |
| Silva C, Mira A (2005) El efecto del pastoreo en la abundancia del tejón Meles meles (Linnaeus, 1758), en la Sierra de Monfurado (Portugal) *Resúmenes VII Jornadas de la SECEM*, Valencia, Spain. | | Conference Proceedings | | Conservation | |
| Silva F, Sargo R, Valente J, Martins Requincha JF, Ribeiro Dias MI, Seixas Travassos F, Rio-Maior H, Álvares F, Antunes, Viegas CA (2012) Papilomatose em Canis lupus signatus: relato de caso. In: *Abstracts of the III Iberian Wolf Congress*, 56. Lugo, Spain. | | Conference Proceedings | | Health status | |
| Silva G (2011) *Impacto dos parques eólicos em grandes mamíferos: o caso do Parque Eólico do Alto da Coutada.* Faculty of Sciences of the University of Lisbon Lisbon, Portugal. | | Thesis | | Conservation | |
| Silva M, Ferreira IB, Guerra D, Deplazes P, Rio-Maior H, Nakamura M, Álvares F, Santos N, Carvalho LMM de (2012) Rastreio de parasitas gastrointestinais, pulmonares e musculares em canídeos domésticos e silvestres no Norte de Portugal. In: *Abstracts of the III Iberian Wolf Congress*, 59. Lugo, Spain. | | Conference Proceedings | | Health status | |
| Silva P (2010) *Identification of highly differentiated molecular markers for the efficient detection of wolf-dog hybrids.* MSc thesis, Faculty of Sciences of the University of Porto, Porto, Portugal. | | Thesis | | Genetics | |
| Silva S (2000) *Estudo das relações sociais do lobo ibérico em cativeiro: influência do sexo, idade e hierarquia.* Final Degree thesis, Faculty of Sciences of the University of Lisbon Lisbon, Portugal. | | Thesis | | Others | |
| Simão LR (1998) *Elementos para o estudo da biologia alimentar da geneta (Genetta genetta L. 1758) (Carnivora, Viverridae) em algumas regiões de Portugal.* Faculty of Sciences of the University of Lisbon, Lisbon, Portugal. | | Thesis | | General ecology | |
| Simões F, Pires AE, Borges C, Amorim do Rosário I, Teixeira T, Petrucci-Fonseca F, Matos J (2012) Paternal lineage diversity of modern wolves and dogs in Iberia. In: *Abstracts of the III Iberian Wolf Congress*, 34. Lugo, Spain. | | Conference Proceedings | | Genetics | |
| Simões G (2010) *Contributo para a delimitação de áreas prioritárias para a conservação de carnívoros na Herdade do Esporão.* MSc thesis, Faculty of Sciences of the University of Lisboa, Lisbon, Portugal. | | Thesis | | Conservation | |
| Simões Graça MA, Ferrand de Almeida F (1983) Contribuição para o conhecimento da lontra (Lutra lutra L.) num sector da bacia do rio Mondego. *Ciência Biológica. Ecology and Systematics* 5: 33–42. | | non-SCI Journals | | Population Status | |
| Simões L (2009) *Factores determinantes da diversidade e abundância de mamíferos num sistema agro-silvo-pastoril mediterrânico.* MSc thesis, Faculty of Sciences of the University of Lisbon, Lisbon, Portugal. | | Thesis | | General ecology | |
| Simões M, Barbosa AM, Vila-Viçosa MJ, Cortes H, Mira A, Padre L (2010) Parasitic diversity of wild carnivores and considerations on their conservation. In: *Symposium “Disease invasion: impacts on biodiversity and human health”, 2010*, (Poster). | | Conference Proceedings | | Health status | |
| Simões P (1977) Uma população de lontras no litoral português. *Boletim da LPN* 16: 17–19. | | non-SCI Journals | | Population Status | |
| Simões P (1986) Que futuro para as populações de lontras da costa alentejana? *Quercus* 5: 16. | | non-SCI Journals | | Conservation | |
| Simões R, Ferreira C, Goncalves J, Álvares F, Rio-Maior H, Roque S, Brandao R, da Costa PM (2012) Occurrence of virulence genes in multidrug-resistant Escherichia coli isolates from Iberian wolves (Canis lupus signatus) in Portugal. *European Journal of Wildlife Research* 58: 677–684. | | SCI Journals | | Health status | |
| Soares FF (2010) *Antropologia e conservação da natureza. O caso de uma possível reintrodução de espécies outrora emblemáticas no Parque Natural da Serra da Estrela (Portugal).* Master thesis, Faculty of Social Sciences and Humanities, Universidade Nova de Lisboa, Lisboa, Portugal. | | Thesis | | Conservation | |
| Soares M, Sarmento P, Cruz J, Eira C, Soares A (1998) Diet of the otter Lutra lutra (L.) in two different habitats in the centre of Portugal. In: Reig S (ed) *Abstracts of the Euro-American Mammal Congress*, Santiago Compostela, Spain. | | Conference Proceedings | | General ecology | |
| Soares N, Gomes P, Macedo L (1998) Influence of fox predation over the lagomorph’s population of Castro Laboreiro: preliminary results. In: Reig S (ed) *Abstracts of the Euro-American Mammal Congress*, Santiago Compostela, Spain. | | Conference Proceedings | | General ecology | |
| Soares NM (2000) *Conflito Homem-Raposa: o exemplo da região de Castro Laboreiro.* MSc thesis, University of Minho, Braga, Portugal. | | Thesis | | Conservation | |
| Soeiro V (2013) *Pseudorabies and tuberculosis: serologic evidence of infection in wild boar populations in southeast Portugal and associated risks for iberian lynx conservation.* MSc thesis, University of Évora, Évora, Portugal. | | Thesis | | Health status | |
| Sousa M (1995) *Ecologia e conservação da lontra (Lutra lutra Linnaeus, 1758) na área do Parque Natural da Serra da Estrela.* Faculty of Sciences of the University of Lisbon, Lisbon, Portugal. | | Thesis | | Conservation | |
| Sousa M (1997) *A lontra no Parque Natural de Sintra-Cascais: distribuição, factores de ameaça e proposta de medidas de conservação*. Instituto da Conservação da Natureza, Lisbon, Portugal. | | Technical report | | Conservation | |
| Sousa M, Machado M, Loureiro A, Bras A, Godinho R, Rio-Maior H, Alvarez F, Esteves PJ (2011) Occurrence and genotype characterisation of Giardia lamblia in wolves (Canis lupus L. 1758) from Portugal. *Clinical Microbiology and Infection* 17: S216. | | SCI Journals | | Genetics | |
| Sousa M, Trindade A, Santos-Reis M (1997) A situação da lontra no Planalto Superior da Serra da Estrela (altitude superior aos 1.500 m). In: *Resúmenes III Jornadas de la SECEM*, Castelló d’Empúries, Gerona, Spain. | | Conference Proceedings | | Population Status | |
| Stohr C, Coimbra E (2013) The Governance of the Wolf-Human Relationship in Europe. *Review of European Studies* 5: 1–18. | | non-SCI Journals | | Conservation | |
| Talegón J, Ribeiro S (2005) Artefactos tradicionales para prevenir daños de lobo en áreas fronterizas de España y Portugal. In: *Abstracts of the II Congresso Luso-Espanhol sobre o Lobo*, Castelo Branco, Portugal. | | Conference Proceedings | | Conservation | |
| Tavares Santos P, Melo P, Loureiro F, Nunes T, Santos N, Santos E (2012) Protocol for the Evaluation of Sanitary Status of Domestic and Wild Fauna That Share Pathologies and Potential Habitat with the Iberian Lynx - Project LIFE+ Enhancing Habitat for the Iberian Lynx and Black Vulture in the Southeast of Portugal. In: *Abstracts of the III European Congress of Conservation Biology*, Glasgow, Scotland. | | Conference Proceedings | | Health status | |
| Teixeira I (2014) *Alterações na dispersão de sementes por carnívoros causadas por alterações no habitat.* MSc thesis, Faculty of Sciences of the University of Lisbon, Lisbon, Portugal. | | Thesis | | Others | |
| Tinoco Torres R, Cruz T, Oliveria B, Carvalho J, Rocha RG, Santos J, Brotas G, Mendes L, Fonseca C (2012) Roe deer (Capreolus capreolus) reintroduction feasibility in central Portugal: Iberian wolf conservation. In: *Abstracts of the III Iberian Wolf Congress*, 46. Lugo, Spain. | | Conference Proceedings | | Conservation | |
| Tomasz S, Wierzbowska I, Santos MJ, Rosalino LM, Santos-Reis M, Eskreys-Wójcik M (2008) Is it worth doing detailed identification of invertebrate species in carnivores’ diet analyses? In: Ronkay MT (ed) *Abstracts of the 26th Mustelid Colloquium*, Budapest, Hungary. | | Conference Proceedings | | Others | |
| Tomé R, Catry P (2008) Atlas da Fauna do Vale do Côa. Município de Pinhel, Pinhel, Portugal. 180 pp. | | Book | | Population Status | |
| Torres J, Feliu C, Fernández-Morán J, Ruíz-Olmo J, Rosoux R, Santos-Reis M, Miquel J, Fons R (2004) Helminth parasites of the Eurasian otter Lutra lutra in southwest Europe. *Journal of Helminthology* 78: 353–359. | | SCI Journals | | Health status | |
| Torres J, Rosalino LM, Miquel J, Santos-Reis M (2000) Helminth coprological survey of the iberian badger (Meles meles marianensis) in Portugal. *Acta Parasitologica* 45: 165. | | SCI Journals | | Health status | |
| Torres J, Santos-Reis M (1997) Helmintos gastrointestinales de Herpestes ichneumon en Portugal *Resúmenes II Jornadas de la SECEM*, Castelló d’Empúries, Gerona, Spain. | | Conference Proceedings | | Health status | |
| Torres J, Santos-Reis M, Feliú C, Trindade A, Ruiz-Olmo J (1997) Espectro vermidiano de Lutra lutra en España y Portugal. In: *Resúmenes III Jornadas de la SECEM*, Castelló d’Empúries, Gerona, Spain. | | Conference Proceedings | | Health status | |
| Torres J, Segovia J-M, Miquel J, Feliu C, Llaneza L, Petrucci-Fonseca F (2000) Helmintofauna del lobo ibérico (Canis lupus signatus Cabrera, 1907). Aspectos potencialmente útiles en mastozoología. *Galemys* 12: 1–11. | | non-SCI Journals | | Health status | |
| Torres RT, Silva N, Brotas G, Fonseca C (2015) To Eat or Not To Eat? The Diet of the Endangered Iberian Wolf (Canis lupus signatus) in a Human-Dominated Landscape in Central Portugal. *PloS one* 10: e0129379. | | SCI Journals | | General Ecology | |
| Trigo MI (1994) *Predação por lontra (Lutra lutra Linnaeus, 1758) em pisciculturas do estuário do Mira.* Faculty of Sciences of the University of Lisbon, Lisbon, Portugal. | | Thesis | | General ecology | |
| Trindade A (1988) *A fauna de mamíferos do Parque Natural da Arrábida: contribuição para o seu conhecimento.* Lisbon. | | Book | | Population Status | |
| Trindade A (1990) Some Observations on the Otter Population in the Homem Catchment (NW Portugal). *IUCN Otter Specialist Group Bulletin* 5: 61–68. | | non-SCI Journals | | Population Status | |
| Trindade A (1991) Fish farming and Otters in Portugal. *IUCN Otter Specialist Group Bulletin* 6: 7–9. | | non-SCI Journals | | Conservation | |
| Trindade A (1991) Some research projects on otters in Portugal. In: *Proceedings of the V International Otter Colloquium*, 263–264. Hankensbuttel, Germany. | | Conference Proceedings | | Others | |
| Trindade A (1996) A lontra Lutra lutra na Reserva Natural do Estuário do Sado. Instituto da Conservação da Natureza, Lisbon. | | Book | | Population Status | |
| Trindade A (1996) *O uso do tempo e do espaço pela geneta (Genetta genetta L., 1758) no Paul do Boquilobo.* MSc thesis, Faculty of Sciences of the University of Lisboa, Lisbon, Portugal. | | Thesis | | General ecology | |
| Trindade A (1997) A lontra em Portugal - estratégia de conservação. In: *Resúmenes III Jornadas de la SECEM*, Castelló d’Empúries, Gerona, Spain. | | Conference Proceedings | | Conservation | |
| Trindade A, Chambel I, Farinha N, Florêncio E, Sousa M (1997) Conservação da Lontra em Portugal Continental: Distribuição e Estatuto de Lutra lutra em Nove Areas Protegidas de Portugal. In: *Resúmenes III Jornadas de la SECEM*, Castelló d’Empúries, Gerona, Spain. | | Conference Proceedings | | Conservation | |
| Trindade A, Farinha N (2002) Otter Distribution in Portugal. In: *Proceedings of the VIIth International Otter Colloquium*, 356–360. Trebon, Czech Republic. | | Conference Proceedings | | Population Status | |
| Trindade A, Farinha N, Florêncio E (1998) A Distribuição da Lontra Lutra lutra em Portugal - situação em 1995. Instituto da Conservação da Natureza, Lisbon. | | Book | | Population Status | |
| Trindade A, Farinha N, Florêncio E, Sousa M (1995) Otter conservation in Portugal: distribution and status of Lutra lutra in five protected areas of the country. *IUCN Otter Specialist Group Bulletin* 12: 15–22. | | non-SCI Journals | | Conservation | |
| Trindade G (2002) *A lontra (Lutra lutra Linnaeus, 1758) na bacia hidrográfica do Sado: distribuição e análise dos arrozais como fonte de recursos tróficos.* Final Degree thesis, Faculty of Sciences of the University of Lisbon Lisbon, Portugal. | | Thesis | | General ecology | |
| Trouwborst A (2014) The EU Habitats Directive and wolf conservation and management on the Iberian Peninsula: a legal perspective. *Galemys* 26: 15–30. | | non-SCI Journals | | Conservation | |
| Valla Pinto MM (1978) *A raposa (Vulpes vulpes silacea Miller, 1907) no Parque Nacional da Peneda-Gerez e na Serra da Cabreira: métodos de estudo; impacto nas populações-presa.* Final Degree thesis, Faculty of Sciences of the University of Lisbon Lisbon, Portugal. | | Thesis | | Conservation | |
| Van Asch B, Alves C, Santos L, Pinheiro R, Pereira F, Gusmão L, Amorim A (2010) Genetic profiles and sex identification of found-dead wolves determined by the use of an 11-loci PCR multiplex. *Forensic Science International: Genetics* 4: 68–72. | | SCI Journals | | Genetics | |
| Van Hafften JL (1982) *Bragança-Zamora wolf project. Report on research carried out in an area in Serra da Nogueira on the iberian wolf (Canis lupus signatus) from 1 March to 15 December 1982.* | | Technical report | | General ecology | |
| Van Heccke I (2000) *Research concerning the earthworm and beetle availability for the badger (Meles meles) in Grândola Mountain, Portugal*. Animal Management, Van Hall Instituut, Leeuwarden, Netherlands. | | Technical report | | General ecology | |
| Vandelli D (1797) *Florae, et Faunae Lusitanicae Specimen.* | | Book | | Populations Status | |
| Vasconcelos R (2003) *Influência de factores ambientais no padrão de actividade e comportamentos do texugo (Meles meles) na Serra de Grândola.* Final Degree thesis, Faculty of Sciences of the University of Lisbon Lisbon, Portugal. | | Thesis | | General ecology | |
| Verdade LM, Rosalino LM, Gheler-Costa C, Pedroso NM, Lyra-Jorge MC (2011) Adaptation of Mesocarnivores (Mammalia: Carnivora) to Agricultural Landscapes in Mediterranean Europe and Southeastern Brazil: a Trophic Perspective. In: Rosalino LM, Gheler-Costa C (eds) *Middle-Sized Carnivores in Agricultural Landscapes*, 1–38. Nova Science Publishers, Inc. | | Book | | General ecology | |
| Vergara M, Basto MP, Madeira MJ, Gómez-Moliner BJ, Santos-Reis M, Fernandes C, Ruiz-González A (2015) Inferring Population Genetic Structure in Widely and Continuously Distributed Carnivores: The Stone Marten (Martes foina) as a Case Study. *PloS one* 10: e0134257. | | SCI Journals | | Genetics | |
| Vicente M (2008) *Efeitos da dimensão e distância de fragmentos de habitats não-matriz para a diversidade de mamíferos do montado de sobro da Serra da Grândola.* MSc thesis, Faculty of Sciences of the University of Lisbon, Lisbon, Portugal. | | Thesis | | General ecology | |
| Vingada JV, Ferreira AJ, Keating AL, Sousa JP, Soares AM, Eira C et al. (1996) *Conservação do lobo (Canis lupus) em Portugal. Fomento e conservação das principais presas naturais do lobo.* Instituto Ambiente e Vida/Instituto da Conservação da Natureza, Coimbra, Portugal. | | Technical report | | Conservation | |
| Vingada JV., Eira C, Ferreira AJ, Carmo P, Cancela JH, Keating AL et al. (1997) Re-introduction of Roe Deer in the centre of Portugal in wolf ocurrence areas. Main goals, status and first results. In: *Resúmenes I Congreso Hispano-Luso. Situación y conservación de las poblaciones de lobo en la Península Ibérica*, Soria, Spain. | | Conference Proceedings | | Conservation | |
| Vingada JV., Eira C, Scheich S, Fonseca C, Soares M, Faria M, Ferreira S (1998) Is it possible to avoid wolf (Canis lupus signatus) extinction in the centre of Portugal? In: Reig S (ed) *Abstracts of the Euro-American Mammal Congress*, 272. Santiago Compostela, Spain. | | Conference Proceedings | | Conservation | |
| Vos J (2000) Food habits and livestock depredation of two Iberian wolf packs (Canis lupus signatus) in the north of Portugal. *Journal of Zoology* 251: 457–462. | | SCI Journals | | General ecology | |
| Yilmaz O, Coskun F, Ertugrul M (2015) Livestock damage by carnivores and use of livestock guardian dogs for its prevention in Europe - A review. *Journal of Livestock Science* 6: 23–35. | | SCI Journals | | Conservation | |
| Zuza (1982) A lontra. Estudos. *Zimbro* 2: 6–7. | | non-SCI Journals | | Population Status | |
